# Phylogenomics and classification of Cactaceae based on hundreds of nuclear genes

**DOI:** 10.1007/s00606-025-01948-z

**Published:** 2025-08-11

**Authors:** Jurriaan M. de Vos, Urs Eggli, Reto Nyffeler, Isabel Larridon, Catherine McGinnie, Niroshini Epitawalage, Olivier Maurin, Félix Forest, William J. Baker

**Affiliations:** 1 Department of Environmental Sciences - Botany, University of Basel, Schönbeinstrasse 6, 4056 Basel, Switzerland; 2 Sukkulenten-Sammlung Zürich, Grün Stadt Zürich, Zurich, Switzerland; 3 Department of Systematic and Evolutionary Botany, University of Zurich, Zurich, Switzerland; 4 Royal Botanic Gardens, Kew, Richmond, Surrey TW9 3AE UK; 5 The New York Botanical Garden, 2900 Southern Blvd, Bronx, NY 10458 USA; 6 Meise Botanic Garden, Nieuwelaan 38, 1860 Meise, Belgium; 7 Fédération Wallonie-Bruxelles - Service général de l’Enseignement supérieur et de la Recherche scientifique, Rue Lavallée 1, 1080 Brussels, Belgium; 8 Department of Biology, Aarhus University, Aarhus, Denmark

**Keywords:** Angiosperms353, Cactaceae, Multispecies coalescent, Paraphyly, Phylogenetic classification, Phylogenetic conflict, Phylogenomics, Rapid radiation

## Abstract

Phylogenetic classification based on evolutionary relationships is the standard approach in systematics, but Cactaceae has posed significant challenges due to the signature of its rapid radiation: low sequence divergence hindering phylogenetic resolution and enormous species diversity hindering attempts to adequately reflect phylogenetic diversity. Previous classifications mostly relied on joint assessment of multiple phylogenetic studies and/or intuition on morphological evolution, lacking comprehensive genomic analysis. Here, we propose a revised phylogenetic classification of Cactaceae, based on the Angiosperms353 set of phylogenomic markers, including 170 species, covering close to 90% of genera and common segregates. Coalescent-based gene tree–species tree reconciliation reveals a well-resolved phylogenetic backbone, mostly congruent with a previous plastid DNA-based summary phylogeny. Some unresolved areas surrounding the subfamilies Cactoideae and Pereskioideae remain however, where gene concordance analyses reveal complex evolutionary histories. We formally recognize the four traditional subfamilies Pereskioideae, Opuntioideae, Maihuenioideae, and Cactoideae, plus Blossfeldioideae and Leuenbergerioideae. Four subfamilies are monogeneric, while within Opuntioideae, we recognize three tribes (Opuntieae, Cylindropuntieae, and Pterocacteae) and within Cactoideae, we recognize eight tribes: Lymanbensonieae, Copiapoeae, Cacteae (incl. subtribes Echinocactinae, Ferocactinae, Cactinae), Phyllocacteae (incl. subtribes Corryocactinae, newly recognized Leptocereinae, Hylocereinae, Echinocereinae), Fraileae, Rhipsalideae, Notocacteae, and Cereeae (incl. subtribes Aylosterinae, Rebutiinae, Gymnocalyciinae, Cereinae, newly recognized Reicheocactinae, Trichocereinae). Our completely revised classification for all clades includes full generic synonymy accepting 155 genera. Overall, the phylogenetic structure of Cactaceae mirrors the angiosperm-wide pattern of enigmatic, species-poor lineages dispersed amongst clades that are orders of magnitude more species rich, revealing a mix of nested, 'explosive' radiations and orphan lineages.

**Supplementary Information:**

The online version contains supplementary material available at 10.1007/s00606-025-01948-z.

## Introduction

Large, species-rich clades resulting from rapid radiations have long remained a major challenge for phylogenetic classification, despite the molecular phylogenetic revolution based on Sanger DNA sequence data since the 1990s. Presently, a second molecular phylogenetic revolution is taking place (de Vos and Stöcklin 2023), as high-throughput sequencing techniques that do not require prior sequence information are becoming established as the new standard approach in phylogenetics. Typically based on selective DNA-library enrichment using short, universal, or clade-specific probes, hundreds of loci can now be cost-effectively sequenced, even from degraded DNA (Brewer et al. 2019; Kistler et al. 2020; Baker et al. 2021; Burbano and Gutaker 2023), allowing for multiple orders of magnitude higher data volumes, now routinely hundreds of thousands to millions of base pairs per taxon. These offer promises of significantly improving (i.e. stabilizing) classifications of traditionally “difficult” groups (e.g. Araceae, Haigh et al. 2023; Cyperaceae, Larridon et al. 2021; Cunoniaceae, Pillon et al. 2021; Rubiaceae, Thureborn et al. 2022; Connaraceae, de Vos et al. 2024).

**Fig. 1:**
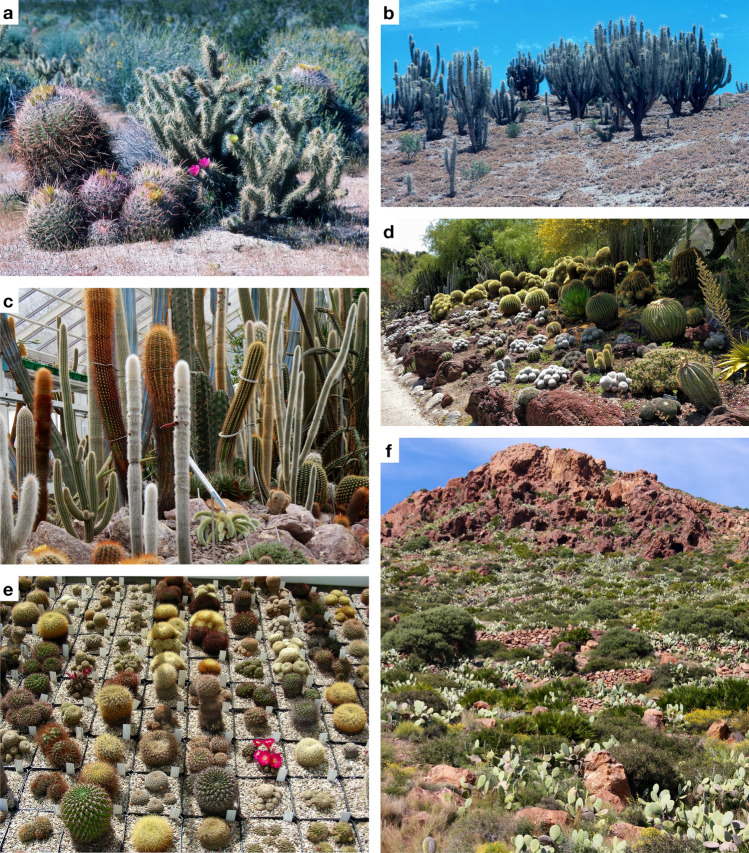
Cacti are attractive and sometimes landscape-dominating elements in both North (**a** Anza Borrego desert, USA, California, with *Cylindropuntia acanthocarpa* and *Ferocactus acanthodes*) and South America (**b** Chile, with *Eulychnia acida* and *Cumulopuntia sphaerica*), ubiquitous elements in Botanical Gardens in glasshouse (**c** Sukkulenten-Sammlung Zürich) and outdoor plantings (**d** Desert Garden at the Huntington Botanical Garden, San Marino, California, USA) around the world, but also fascinating collectibles and study objects for amateur collections (**e** a collection of *Weingartia* species), but also invasive neophytes in suitable frost-free climates worldwide (**f** *Opuntia ficus-indica* naturalized in Spain, Andalusia, Almería, Níjar, Las Negras). Credits: a-c, e Urs Eggli, d CC-BY Flickr cultivar413, f Felix Merklinger

A particularly striking example of such highly diverse, poorly resolved clades is the cactus family, Cactaceae (Fig. 1), in the Portulacineae suborder of Caryophyllales. As a natural group, Cactaceae are well characterized by the presence of distinct spine clusters (i.e. areoles) that are derived from contracted short shoots whose leaves are transformed into spines, covered by an indumentum of multicellular trichomes (Nyffeler and Eggli 2010a). Their floral morphology is also peculiar: The vast majority of species have an inferior ovary covered by stem tissue enclosing the carpels, covered with areoles and spines, and a more or less distinctive hypanthium with a graded series of perianth elements (Barthlott and Hunt 1993). The circumscription of the family as a natural group has therefore been very stable, with currently some 124–150 genera and 1438–1882 species recognized (Anderson 2005, Hunt et al. 2006; Korotkova et al. 2021). The family is best known for its iconic landscape-dominating tree-sized species such as *Carnegiea gigantea* in the Sonoran Desert or *Leucostele atacamensis* in the Andes, but also includes diminutive semi-geophytic globose forms such as *Blossfeldia liliputana* from the foot of the Eastern Andean slopes of Argentina and Bolivia and *Mammillaria saboae* from Mexico, or tropical rainforest epiphytes, such as *Selenicereus* spp. With the exception of *Rhipsalis baccifera* occurring in Africa, Madagascar, and Sri Lanka, the family has an entirely New-World distribution, from Southern Argentina to northern British Columbia, Canada, with centres of generic and species diversity in the southwestern United States, arid Mexico, the central Andes of Peru and Bolivia, and eastern Brazil (Nyffeler and Eggli 2010a; Barthlott et al. 2015), where they are increasingly under threat (Pillet et al. 2022). The family spans an enormous ecological breadth from tropical lowland rainforest to montane forests to semi-deserts (Sonoran Desert, Chihuahuan Desert, Andean mountains), deserts (fringes of the Atacama desert), and high-mountain cold-arid habitats. Accordingly, they display a range of specialized ecological adaptations, of which the most well-known include Crassulacean acid metabolism (CAM) photosynthesis, stem succulence, and a reduction of leaves. Remarkably, and contrary to early assumptions that Cactaceae represent an old phylogenetic lineage (e.g. Backeberg 1942), their extant species diversity originated relatively recently from the mid-Miocene onwards in association with continental-scale aridification (Arakaki et al. 2011), spurred by a combination of biotic and abiotic drivers (Hernández-Hernández et al. 2014; Thompson et al. 2024), jointly making them an important study group for plant radiations and the evolution of morphological and ecological diversity.

In stark contrast to the stability of the circumscription of Cactaceae as a family, its internal classification has always been rather unstable, both in the pre-DNA era (Anderson 2001, 2005; Metzing and Kiesling 2008) and in the molecular era (Nyffeler and Eggli 2010a; Guerrero et al. 2019; Korotkova et al. 2021; Romeiro-Brito et al. 2023b). This instability arose for two main reasons: First of all, Cactaceae display a great diversity of morphology that is often—intuitively—perceived as “trends”, e.g. towards increasingly extreme life forms, semi-formalized in a “law of the abbreviation of the vegetative phase” by Franz Buxbaum (1956). However, molecular studies continue to reveal a large amount of homoplasy in many characters previously used to circumscribe higher taxa, resulting in a scarcity of true phenotypic synapomorphies for clades (Hernández-Hernández et al. 2011). The disparity between vegetative morphology and DNA is evidence for a high level of morphological convergence and parallelism, confounding the understanding of species relationships and character evolution (Copetti et al. 2017). A case in point is *Astrophytum*, in which *A. caput-medusae* displays long thin tubercles, while the remaining ca. four species have ribs, or *Leuchtenbergia*, a monotypic genus with long trigonous tubercles nested within a clade composed of rib-bearing *Stenocactus* and *Ferocactus* species (Hernández-Hernández et al. 2011; Vázquez-Lobo et al. 2016). Strikingly, in turn, the many species traditionally assigned to *Ferocactus*—in a broad-sense morphological concept—are morphologically coherent but display comparatively substantial molecular divergence across several clades (Nyffeler and Eggli 2010a; Vázquez-Sánchez et al. 2013).

The second reason for the comparatively unstable infrafamilial classification results from the molecular signatures of the family’s very rapid radiation. Cactaceae have amongst the top five highest family-level diversification rates of any plant family, due to their comparatively young age and high species diversity (Hernández-Hernández and Wiens 2020) and contain multiple, nested radiations (Hernández-Hernández et al. 2014; Thompson et al. 2024). This rapid radiation resulted on the one hand in DNA sequence divergence across species generally so low that Sanger sequencing methods have had mixed success in resolving infrafamilial relations and especially in settling generic delimitation. These methods nevertheless have pointed out inconsistencies between morphological classifications and evolutionary history that may contradict the intuition of well-informed specialists (Nyffeler and Egli 2010a). On the other hand, phylogenomic approaches have revealed particularly high conflict across gene trees at several nodes (Moore et al. 2018, Wang et al. 2019; Acha and Majure 2022; Romeiro-Brito et al. 2022; Romeiro-Brito et al. 2023b), as expected in this clade with rapid species diversification and incidence of polyploidy (Yang et al. 2018; Castro et al. 2020). This conflict surrounds, for instance, the position of Cactaceae relative to Anacampserotaceae, Portulacaceae and Talinaceae within Caryophyllales (Moore et al. 2018), the position and delineation of its subfamilies (specifically, the appropriate concept for Pereskioideae and the position of Maihuenioideae), as well as resolving a suit of internal nodes. Importantly, the family’s enormous species diversity also hindered adequate taxon sampling, resulting in phylogenetic studies overall either poorly reflecting species diversity across the family (Acha and Majure 2022), or considering few genetic loci (Thompson et al. 2024).

Beyond classification, these challenges also hinder the understanding of evolutionary patterns and processes. For instance, the traits characterizing subfamilies Leuenbergerioideae and Pereskioideae (i.e. leaf-bearing, hardly succulent, shrubby or arborescent plants), the putatively consecutive sisters to the rest of Cactaceae, are usually extrapolated as ancestral for the family (e.g. Griffith 2008:40; Edwards et al. 2005). However, Cactaceae contain several diminutive, species-poor lineages that were thought to be highly derived but appear to be closer to the crown node of the family than previously expected (including *Maihuenia* and *Blossfeldia*). This brings into question the general notion that shrubby or arborescent growth is indeed ancestral for the family, in particular because the families most closely related to Cactaceae also consist mostly of small herbaceous plants, often with fleshy taproots (Nyffeler and Eggli 2010a, 2010b). Similarly, leaf evolution in Opuntioideae may be more homoplasious than is commonly assumed (Majure et al. 2023).

Clearly, an improved understanding of the family’s remarkable diversification history and its enigmatic morphological and ecological trait evolution would represent major progress towards improved understanding of the origins and drivers of the diversity of life. The first step towards this major overarching goal is the proposing of a phylogenetic classification for Cactaceae that is putatively stable by reflecting actual evolutionary history. This requires the inferring of a new, robust phylogenetic hypothesis for the entire family, rather than jointly interpreting a suit of studies based on partial taxon sampling, while leveraging a large number of nuclear loci for a broad set of well-identified species that cover all putative major clades. It also requires carefully interpreting genetic distinctness of putative, named higher taxa, while evaluating conflicting information arising from different parts of the genome: This may reveal that evolutionary histories are more complex than a perfectly dichotomizing species topology would suggest. Finally, we consider practicability by recognizing taxa that can be easily identified.

The goals of the current study are to evaluate the monophyly and genetic distinctness of all suprageneric taxa proposed by Nyffeler and Eggli (2010a) and propose a revised classification for the entire family, based on formal analysis of a new data set generated using targeted enrichment with the Angiosperms353 probe set (Johnson et al. 2019). Although these data were partially included in Zuntini et al. (2024), the low sequence divergence and high gene conflict in this part of the angiosperm tree of life required the present targeted and much expanded analyses. Based on over three hundred nuclear loci for 170 taxa, representing over 36 million base pairs, of which 95% was newly sequenced for this study, we present a classification that includes six subfamilies, 11 tribes, and 14 subtribes (including two new ones), each with a description, a discussion on its delineation, and a list of included genera, including full generic synonymy. The most striking features of this work, in which all previously “orphaned” genera are now placed, include the recognition of the subfamilies Leuenbergerioideae and Blossfeldioideae, and a clarified circumscription of the Cactoideae tribes Lymanbensonieae, Copiapoeae, and Fraileae. That these higher taxa are greatly divergent in species numbers is a consequence of the diversification history of this remarkable family.

## Material and methods

### Taxon sampling

Taxon sampling aimed to include one representative of all genera of Cactaceae, including the most important generic segregates with debated concepts. To define our targets for taxon sampling, we first made a list including all accepted genera in Nyffeler and Eggli (2010a) and Hernández-Ledesma et al. (2015), and then added occasionally recognized generic segregates. Next, we identified the type species for each, and attempted to obtain fresh tissue for it or, if not available, from a morphologically close species (211 taxa obtained). After attempting DNA extraction and sequencing, we obtained adequate, novel data for 163 Cactaceae taxa plus five outgroup taxa, to which we added high-quality, publicly available sequencing reads from eight additional samples. Because *Maihuenia* was included from public and our own data (see below), in total we included 176 samples representing 175 species, including de novo sequence data for 168 taxa (voucher and data availability statements are provided in Online Resource 1). Even though this represents less than 10% of the species diversity, it represents 87% of the genera in the latest checklist (Korotkova et al. 2021; the missing ones being *Brachycereus, Cochemiea, Consolea, Cumarinia, Epithelantha, Estevesia, Homalocephala, Jasminocereus, Kadenicarpus, Kimnachia, Kroenleinia, Leucostele, Lymanbensonia, Mila, Miqueliopuntia, Neoraimondia, Obregonia, Pediocactus, Quiabentia, Rauhocereus, Rimacactus, Strophocactus,* and *Uebelmannia*).

### Experimental design

Our approach was based on the premise that named higher taxa should in principle be represented by unambiguously well-supported, phylogenetic nodes and be genetically distinct from other such taxa. Accordingly, we employed a two-pronged approach, based on the same set of DNA sequence data, including a comprehensive phylogenomic analysis, plus an analysis based on multidimensional scaling (MDS) of genetic distances (Cox and Cox 2001). Because Cactaceae phylogenetics has suffered from low sequence divergence in traditionally used markers (e.g. Nyffeler and Eggli 2010a; Thompson et al. 2024), and phylogenomic approaches revealed profound conflict across gene trees (e.g. Wang et al. 2019), we conducted a comprehensive set of phylogenomic analyses, representing different resolutions of the trade-off between the total amount of DNA sequence data included and potential for noise. Amongst the available phylogenomic approaches, we chose to selectively amplify the 353 angiosperm nuclear loci (Johnson et al. 2019), which are known to perform comparatively well in Cactaceae (Acha and Majure 2022) and are the most broadly applied set of loci across angiosperms (Zuntini et al. 2024). Other probe sets (e.g. the Cactaceae591, Romeiro-Brito et al. 2022) are similarly useful within Cactaceae, but were not yet available at the start of our project and share fewer loci with the emerging angiosperm-wide datasets (Zuntini et al. 2024). We base our classification on one selected phylogenetic analysis, while making sure named nodes are also recovered in other analyses and are recovered as distinct clusters in a multidimensional scaling analysis of genetic distances.

### Molecular and bioinformatic methods

DNA extraction, library preparation, target enrichment, and DNA sequencing follow Baker et al. (2022). Briefly, using a modified CTAB protocol (Doyle and Doyle 1987), we extracted DNA which we then fragmented using sonication (Covaris M220 Focused-ultrasonicator with microTUBEs AFA Fiber Pre-Slit Snap-Cap; Woburn, MA, USA) when DNA fragment length exceeded 350 bp. We prepared Dual-indexed libraries for Illumina sequencing using the DNA NEBNext UltraTM II Library Prep Kit at half the recommended volume, with Dual Index Primers Set 1, NEBNext Multiplex Oligos for Illumina (New England BioLabs). After pooling 20–25 DNA libraries (equimolar for a total of 1 μg of DNA), we hybridized them using the Angiosperms353 v1 expert panel (Arbor Biosciences, Ann Arbor, MI, USA; Catalog #308,196; Johnson et al. 2019) at 65 °C for 28–32 h. After amplifying enriched products for 10 cycles and cleaning them, we quantified and multiplexed them and then sequenced them on an Illumina MiSeq (v3 reagents, 2 × 300-bp paired-end reads, Illumina, San Diego, CA, USA) at the Royal Botanic Gardens, Kew, or on an on Illumina HiSeq (2 × 150-bp paired-end reads) at Genewiz (Takeley, UK). Sequencing reads were made publicly available through the European Nucleotide Archive (bioproject PRJEB35285) and are included in the Kew Tree of Life explorer (Baker et al. 2022).

Bioinformatic processing and phylogenetic inference was performed on the sciCORE (http://scicore.unibas.ch/) scientific computing centre at the University of Basel. We processed raw sequencing reads by removing adapters and trailing low-quality bases using Trimmomatic v.0.39 (Bolger et al. 2014) with default settings. We assembled sequences using the HybPiper v.1.3.1 pipeline (Johnson et al. 2016). For each of the 353 target loci, it uses BWA (v.0.7.15, Li and Durbin 2009) to select relevant reads using a custom target file (created by selecting Caryophyllales sequences from the 'mega353' target file; McLay et al. 2021) and assembles them de novo using SPAdes (v.3.10.1, Bankevich et al. 2012). We carried the extracted exon sequences forward without attempting to include intron sequences, because trial runs revealed that extracting them made locus alignments problematic and did not yield considerable additional phylogenetic information.

### Phylogeny inference

Our phylogenomic analyses considered four DNA sequence data sets (Table 1). From the recovered set of gene sequences, we first created alignments after eliminating samples and loci for which sequence reconstruction was poor (dataset QC, for quality control), and a second set of alignments in which we additionally excluded potentially paralogous sequences (dataset QC-P, with P for paralogs). For dataset QC, to not bias our inference by poor data quality, we set relatively conservative thresholds to retain only those taxa for which we recovered at least 50% of the loci and the overall recovered sequence length was at least 30% of total target-gene length. We made one exception, by keeping our sample of *Maihuenia poeppigii* (136 loci recovered = 38.5%; 26′463 bp or 8.7% total length recovered) from a live accession (at Sukkulenten-Sammlung Zürich (ZSS), Switzerland), because it represents a monogeneric subfamily. To account for its lower sequence recovery, a second accession of this species was also included from publicly available data but without a verifiable voucher (NCBI biosample SAMN10132135). We also excluded 33 loci that did not meet one or both of the following sequence recovery criteria: recovered in at least 50% of the retained samples (8 loci) and total recovered length at least 40% of the target sequence length (28 loci; Online Resource 2). Two samples for which we could not exclude label switching were conservatively excluded. For Dataset QC-P we applied an additional quality criterion, by first removing potentially paralogous loci and individual paralogous sequences prior to aligning the sequences (Online Resource 2). To this end, we filtered reconstructed sequences using HybPhaser (v.2.0, Nauheimer et al. 2021). HybPhaser flags genes that display excessive heterozygosity (which can occur if multiple, somewhat divergent, gene copies are jointly assembled into a single sequence) by identifying single nucleotide polymorphisms (SNPs) through backmapping raw reads to the reconstructed sequences. We excluded the 17 loci of which the mean percentage of SNPs across all samples was particularly high (threshold at 3%) plus individual sequences from each species that were statistical outliers (0 to 27 sequences per sample, total sequences excluded 2753, mean 15.3 per sample; Online Resource 2). We aligned each locus in both datasets using MAFFT (v.7.490, “localpair” option, Katoh and Standley 2013) and computed its maximum likelihood gene tree using RAxML-NG (v.1.1.0, GTR + G substitution model; Kozlov et al. 2019) with support values based on 100 bootstrap replicates. Next, we created two subsets of dataset QC-P in which we additionally excluded loci that gave rise to poorly supported gene trees based on average bootstrap thresholds of 40 and 50 (datasets QC-P-BS40 and QC-P-BS50, respectively; BS for bootstrap support). Salient dataset properties are provided in Table 1 and details in Online Resource 2.

**Table 1:** Properties of the four phylogenetic datasets

Dataset properties						Quality indicators			
Name	Criterion	Number of tips	Number of gene trees	Locus-by- species matrix occupancy	Total aligned length	Mean BS across gene trees (RAxML)	Normalized quartet score (Astral)	Mean local posterior probability (Astral)	Mean gene concordance factor (Astral)
QC	Good quality sequences (see text)	177	320	95.4%	299′745 bp	29.3	0.681	88.0%	15.4
QC-P	QC, plus no indication of possible paralogy based on SNP	177	299	90.1%	273′475 bp	30.3	0.697	88.0%	16.6
QC-P-BS40	QC + P, plus mean bootstrap support at least 40	177	62	93.5%	87′152 bp	46.9	0.793	83.4%	26.9
QC-P-BS50	QC + P, plus mean bootstrap support at least 50	177	15	98.6%	30′189 bp	56.4	0.825	78.1%	32.7

The gene trees obtained from each of the four datasets were subjected to a gene tree–species tree reconciliation analysis based on ASTRAL-III (v. 5.7.7, Zhang et al. 2018). Astral deconstructs gene trees into individual quartets and puzzles these together, searching for the species tree that maximizes the number of quartets that are congruent with it. In doing so, it considers incomplete lineage sorting as the possible explanation for incongruent quartets; Astral is thus consistent with the multispecies coalescent. The normalized quartet score is the proportion of quartets that are in the species tree and gives a good indication of the overall amount of phylogenetic conflict in the data set. Astral was run with setting very short branches in the gene trees to zero as this eliminates topological information from putatively spurious branches and may thus reduce overall conflict and improve reconstructions (Zhang et al. 2018). Support for a branch in Astral is expressed as local posterior probability and is based on the fraction of quartets that support it. In addition, we expressed branch support by tallying the fraction of “decisive” gene trees that contained it. Hereto we employed the gCF scores (gene concordance factors) implemented in IQ-tree v.2.0 (Minh et al. 2020). In total, this resulted in four Astral species trees. To select our preferred analysis, we considered the total amount of input sequence data, mean bootstrap support of input gene trees, mean local posterior probability, and normalized quartet scores, but also compare topologies from across analyses to evaluate the monophyly of the higher taxa proposed in the classification of Nyffeler and Eggli (2010a).

### Genetic distinctness

In addition to these phylogenetic analyses, we investigated the major axes of overall sequence divergence using a multidimensional scaling approach (MDS; Cox and Cox 2001), implemented in the function cmdscale of the default stats package of the statistical software R v.4.2.1 (R Core Team 2022), based on Mardia’s (1978) algorithm with k = 8. This approach has the added value that genetic distinctness of clades recovered in the phylogenetic analysis can be more intuitively interpreted than from branch lengths generated by Astral, because the latter does not contain information on terminal branch lengths and are not in units of substitutions per site. However, MDS is distance-based and thus phenetic in nature and is therefore not used as a criterion to recognize taxa. Rather, it constitutes one of multiple lines of evidence to understand the differentiation amongst taxa, as it provides information on the structure of sequence variation upon which phylogenetic analyses are based. In turn, this may help interpreting the phylogenetic results upon which classification decisions are based. Genetic distances were based on a Kimura's 2-parameters distance model (Kimura 1980), implemented in the function dist.dna (with the option pairwise.deletion set to true) of the R-package ape (v. 5.4–1, Paradis and Schliep 2019), based on a concatenation of all QC alignments, in which sites with more than 50% missing data had been pruned. MDS projects the distance between objects in a low-dimensional space and is commonly employed in comparative genomics. A cluster of points projected close together represent species that are genetically similar relative to other such clusters, which in our context of classification would be expected if the represented species in a cluster are part of the same named higher taxon. Visualizations of the first two MDS axes illustrate the overall distinctness of individual clusters of taxa.

### Phylogenetic conflict

The phylogenetic relationships surrounding *Pereskia* warrant special attention, because they are widely recognized to be associated with profound phylogenetic conflict. Specifically, on morphological grounds, the species traditionally placed in *Pereskia* are rather similar and jointly so divergent from other Cactaceae that they should be excluded from any other subfamily. Therefore, they were united in Pereskioideae. However, phylogenomic studies generally found two distinct *Pereskia* lineages as subsequent sisters to the remainder of Cactaceae, rendering the traditional Pereskioideae paraphyletic, but consistently with low support (Moore et al. 2018, Walker et al. 2018; Yang et al. 2018; Wang et al. 2019, Acha and Majure 2022; but see Majure et al. 2023). Thus, the principle of recognizing monophyletic taxa requires us to investigate in more detail whether *Pereskia* in a broad circumscription is monophyletic or paraphyletic, where the latter would necessitate recognizing *Leuenbergeria* (either within its own subfamily Leuenbergerioideae (Mayta and Molinari 2015) or, less desirably, leaving them unplaced). Problematically however, depicting evolutionary relations as strictly bifurcating trees in the face of severe phylogenetic conflict is not necessarily appropriate, as branch support values in such cases may be misleadingly high (Steenwyk et al. 2023). For these reasons, we designed an additional analysis to obtain specific phylogenetic evidence for alternative classification decisions.

In particular, we quantified evidence for alternative phylogenetic resolutions using BUCKy v.1.4.4 (Larget et al. 2010), a fully Bayesian approach based on the posterior distribution of gene trees. BUCKy estimates the genome-wide concordance factor (CF) support for each relationship found across individual loci, identifying groups of genes supporting the same topology, while accounting for uncertainty in gene tree estimates. BUCKy thus alleviates the concern that methods used to reconcile gene trees, such as ASTRAL, may overestimate confidence in the species tree (Leaché and Rannala 2011), by highlighting genomic conflict.

Because BUCKy requires each taxon to be present in each gene tree, we selected from the QC-P-BS40 dataset a "prime" set of 29 loci, each pruned to a single accession for each tribe and subfamily, but including three accessions representing both *Pereskia* s.l. lineages (Online Resource 3). Loci were each subjected to a full MrBayes 3.2 analysis (Ronquist et al. 2012), using a GTR + G model of substitution, 10′000′000 mcmc generations, thinned to 2000 samples, of which 25% was discarded as burn-in. BUCKy processed them using default options and 1′000′000 generations. We then computed the primary concordance tree with CF support for all backbone nodes and selected alternative resolutions, including scenarios of para- and monophyly for *Pereskia* s.l.

## Results

### Differences between phylogenetic datasets

The four phylogenetic datasets represented different approaches to the trade-off between the amount of data included and the amount of noise generated (alignments and gene trees for each locus are available in Online Resource 4). This is evident from the sizes of the dataset and several quality indicators (Table 1). Filtering for paralogs reduced dataset QC containing 177 samples and 320 loci (299,745 bp aligned length; locus-by-species matrix containing 320 × 177 cells, 56,640 total, being occupied for 95.4%) to 299 loci, without losing taxa, and a 90.1% locus-by-species matrix occupancy at 273,475 bp aligned length. When additionally filtering for the average quality of the gene trees, QC-P was reduced to 62 gene trees for QC-P-BS40 (87,152 bp total aligned length) or 15 gene trees for QC-P-BS40 (30,189 bp total aligned length). In contrast, the quality of the datasets increased (Table 1), from average bootstrap support per gene tree of 29.3 in QC to 56.4 in QC-P-BS50. Interestingly, the species tree quality did not improve in all aspects from QC to QC-P-BS50 (Table 1): the normalized quartet score and the mean gene concordance factor (both reflecting the amount of congruence between gene trees) increased from 0.68 to 0.83 and from 15.4 to 32.7, respectively, but the average local posterior probability decreased from 0.88 to 0.78. While these moderate normalized quartet scores indicate that incomplete lineage sorting is modest, rather than excessive, the low number of gene trees meeting our bootstrap requirements and the rather low gene concordance factors reveal that there is nevertheless considerable conflict across gene trees, congruent with the expected low phylogenetic resolution in each gene tree in a rapid radiation. Nonetheless, the topologies of the four resulting Astral trees were highly congruent, mostly differing in nodes that were poorly supported in other analyses (compare tree figures in Online Resource 5–8; Table 1). Due to the comprehensive genus-level taxon sampling and phylogenetic congruence across analyses, our study presents a solid and representative basis of all clades for a revised classification.

### Phylogenetic relations

**Fig. 2:**
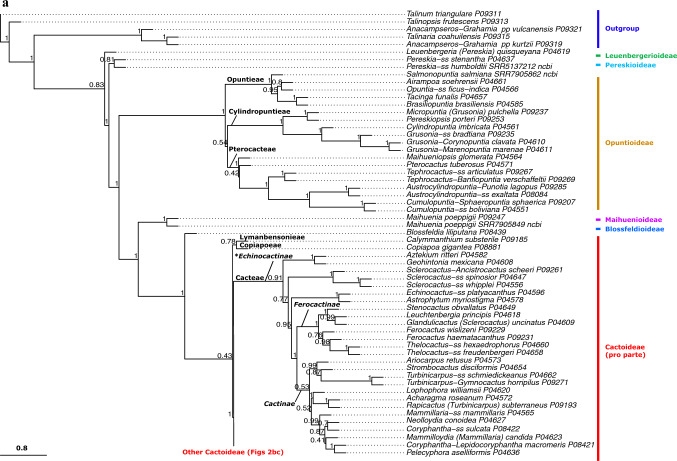

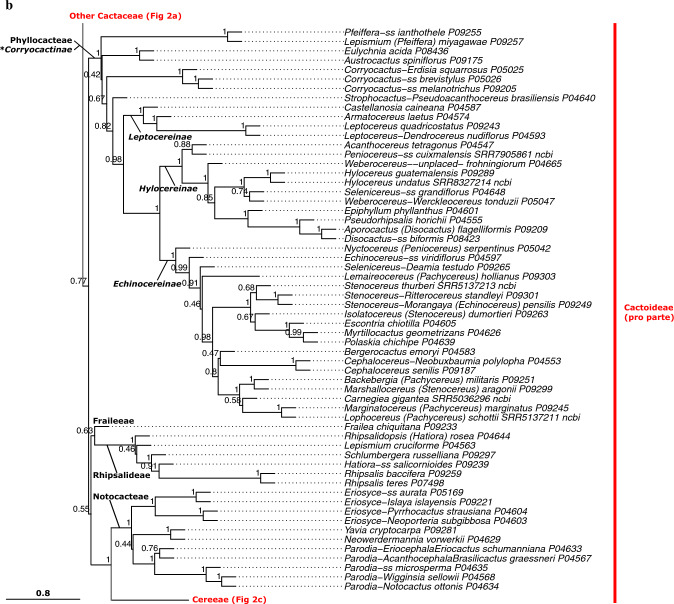

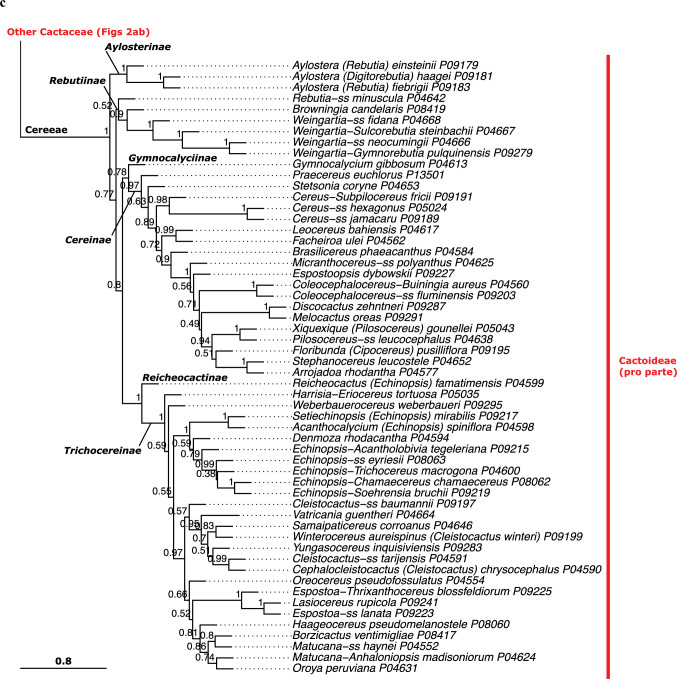
**a** ASTRAL tree (*pro parte*) based on the QC data set. Branch support is indicated above branches as local posterior probability. Suprageneric names are marked: subfamilies are colour-coded to the right, while tribes and subtribes are labelled at their corresponding stem lineage. Higher taxa for which monophyly could not be confirmed are marked with an asterisk (*). Tips are labelled according to their accepted genus, as listed in Table 3. Alternative genus names are provided after a dash for species that represent lineages commonly treated as separate genera, while "ss" is indicated if a species represents the type lineage of a genus that is commonly split. Genus names in brackets indicate taxa that were previously placed in a larger genus complex. P and SRR numbers correspond to newly obtained and public data, respectively, and are linked to Online Resource 1 for accession details. The scale bar represents coalescence units. **b** ASTRAL tree (*pro parte*) based on the QC data set. Annotation as in (**a**). **c** ASTRAL tree (*pro parte*) based on the QC data set. Annotation as in Fig. 2a

**Fig. 3:**
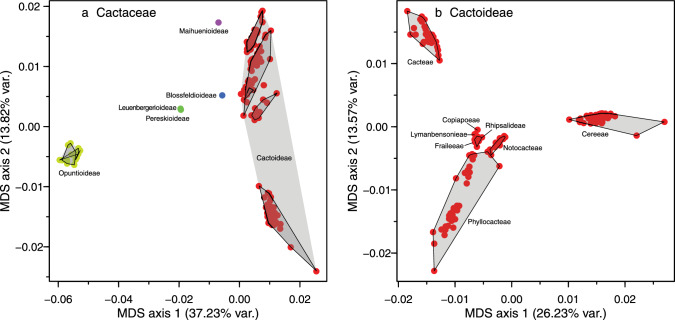
Multidimensional Scaling (MDS) analysis of genetic distinctness across (**a**) all Cactaceae with subfamilies indicated and **b** Cactoideae with tribes indicated, based on concatenated alignments of the QC dataset. Colours indicate subfamilies and polygons indicate non-monogeneric tribes

**Fig. 4:**
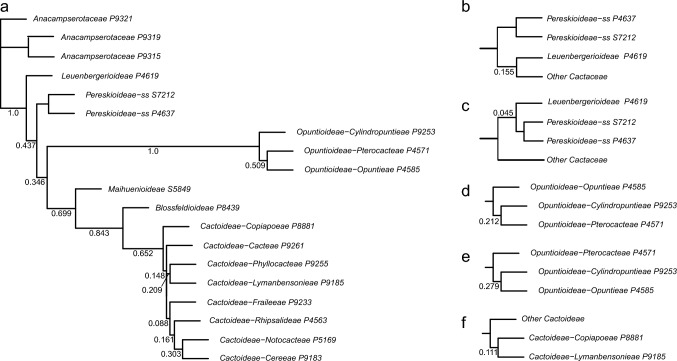
BUCKy analyses of main and alternative topologies. **a** Primary concordance tree with internal nodes proportional to coalescence units and sample gene concordance factors indicated. b-f) Subtrees with sample gene concordance factors indicated for five selected, alternative topologies that deviate from the primary concordance tree: **b**, **c** surrounding *Pereskia* (*where ss* indicates the sensu stricto lineage of Pereskioideae) **d**, **e** surrounding Opuntioideae, **f** surrounding Cactoideae. Tip names indicate higher taxa, and accession numbers indicate species listed in Online Resource 1

Given the strong similarities of the phylogenetic results across datasets (Online Resources 5–8), we base our interpretation primarily on the QC Astral tree (Fig. 2abc, Online Resource 5), because it based on the largest set of data and yielded high-quality indicator values (Table 1). Our phylogenetic analyses were mostly congruent with the classification of Nyffeler and Eggli (2010a) but provide more resolution in detail and result from a much broader sampling strategy. Here, we summarize the salient features of our results, while the classification section discusses all relations in great detail. We find the *Leuenbergeria* lineage of *Pereskia* s.l. (i.e. *L. quisqueyana*) sister to the rest of Cactaceae in most analyses (Figs. 2a, Online Resources 5–7, Astral local posterior probability, LPP 1.0), but separated with a very short branch from *Pereskia* sensu stricto, while the branch separating both lineages from the remainder of Cactaceae is much longer (Fig. 2a, Online Resources 5–7). Using dataset QC-P-BS50, the branching order of both Pereskioid lineages is reversed (Fig S4, LPP 0.92). These results jointly indicate that the crown of Cactaceae remains a tangle, with the two Pereskioid lineages both sharing comparatively little evolutionary divergence with the remainder of Cactaceae. This is also reflected in the MDS analysis (Fig. 3) that revealed virtually no difference between the two Pereskioid lineages and a shared, strong differentiation from the rest of Cactaceae. Despite the similarity of *Leuenbergeria* and *Pereskia* s.s., the BUCKy analysis supports a scenario of them being subsequent sisters to the remainder of Cactaceae, because all competing scenarios receive much lower support (Fig. 4). Thus, we chose to accept subfamily Leuenbergerioideae for the sake of obtaining monophyly for all early-diverging clades, because the two clades are corroborated by a number of morphological and anatomical characters (see Classification chapter for details).

Opuntioideae is very well supported (LPP 1.0 in all analyses, high gene concordance factors, Figs. 2a, Online Resources 5–8) and falls into three clades that we can recognize at the tribal level (Opuntieae, Cylindropuntieae with LPP 1.0 in all analyses; support for Pterocacteae decays from LPP 0.95–1.0 to LPP 0.49 in QC-P-BS50). The subfamily is mostly very well-resolved internally, with the exception of the position of *Maihueniopsis* within Pterocacteae (Fig. 2a) and the relative branching order of the three tribes, where the Astral tree (Fig. 2a) depicts Pterocacteae as sister to Cylindropuntieae and the BUCKy result (Fig. 4) prefers a sister relation to Opuntieae.

The next consecutive branch, *Maihuenia,* is maximally supported as sister to the remainder of Cactaceae (i.e. Cactoideae in its traditional broad circumscription, consisting of *Blossfeldia* plus Cactoideae s.s.), confirming its isolated position. Strikingly, the enigmatic and morphologically distinct genus *Blossfeldia* is the sister to Cactoideae s.s. in all analyses and importantly rather isolated (note that the length of the stem lineage of *Blossfeldia* + Cactoideae s.s. is of similar length as the stem lineage of Cactoideae s.s.; Fig. 2a), although moderate support for Cactoideae s.s. indicates phylogenetic conflict across gene trees (Online Resources 5–8). Nevertheless, its isolation is reinforced by the MDS analysis, where the genetic divergence of *Blossfeldia* from Cactoideae s.s. is similar to that from *Maihuenia* (Fig. 3), and corroborated by high concordance factors in the BUCKy analysis (Fig. 4). Thus, its phylogenetic isolation, genetic distinctness, and aberrant morphology (e.g. absence of cortical bundles, an unusual epidermis, poikilohydry, and miniature stature) justify accepting Blossfeldioideae.

Within Cactoideae s.s., the genera *Copiapoa* and *Calymmanthium* are recovered as joint sister to the remainder of the subfamily in most analyses, but the relations around the base of Cactoideae s.s. are not well supported in any analyses, congruent with a putative ancient rapid radiation. The seven lineages of Cactoideae s.s. that are consistently recovered are *Calymmanthium*, *Copiapoa*, Cacteae, Phyllocacteae, *Frailea*, Rhipsalideae, and Notocacteae plus Cereeae, but their relative branching differs across analyses (Online Resources 5–8). In our preferred analysis, the three lineages *Calymmanthium* plus *Copiapoa,* Cacteae and Phyllocacteae are recovered as subsequent sisters to a clade of *Frailea* plus Rhipsalideae plus Notocacteae plus Cereeae (Fig. 2). Intriguingly, both Cacteae and Phyllocacteae contain a poorly resolved basal grade that can be morphologically characterized supporting recognition of paraphyletic subtribes (see Classification part, where we consistently mark paraphyletic taxa with a * and provide detailed justification; *Echinocactinae and *Corryocactinae, respectively), followed by two (Ferocactinae and Cactinae) or three (Leptocereinae, Hylocereinae, Echinocereinae) clades, respectively, subtended by mostly well-supported branches in all analyses. A sister relation of the genus *Frailea* with a well-supported Rhipsalideae is suggested in the QC and QC-P analyses. Notocacteae is maximally supported as sister to Cereeae in all analyses. Cereeae is separated by a long, well-supported branch in all analyses (Fig. 2c, Online Resources 5–8) and genetically remarkably distinct from all other Cactoideae (Fig. 3). The base of Cereeae, however, again consists of short branches with medium support, congruent with an ancient nested rapid radiation. The four main consistently recovered lineages within Cereeae are *Aylostera* (formerly placed in *Rebutia*; i.e. Aylosterinae), a clade of *Rebutia* s.s., *Browningia* and *Weingartia* (i.e. Rebutiinae), *Gymnocalycium* plus Cereinae, and *Reicheocactus* plus Trichocereinae. Only in the smallest, most stringently filtered dataset (QC-P-BS50) is the clade of *Rebutia* s.s., *Browningia*, and *Weingartia* no longer recovered (Online Resource 8). All analyses reveal strong evidence for the polyphyly of several larger genera in their mostly outdated but widely used circumscriptions, including *Grusonia* in Cylindropuntieae; *Sclerocactus*, *Turbinicarpus*, *Mammillaria,* and *Coryphantha* in Cacteae; *Weberocereus*, *Echinocereus*, *Stenocereus*, and *Pachycereus* in Phyllocacteae; and *Rebutia*, *Echinopsis*, *Cleistocactus*, and *Espostoa* in Cereeae (details in the Classification part).

### Genetic distinctness

The MDS analysis revealed multiple clearly distinct clusters of species, which provides information on overall genetic differentiation between groups of species, which is helpful because ASTRAL branch lengths are in coalescence units (rather than DNA substitutions per site) and undefined for terminal branches. The first two axes captured 37.2% and 13.82% of variation, respectively (Fig. 3). The analysis across Cactaceae revealed six clusters of species: three of the four traditionally recognized subfamilies (i.e. Pereskioideae incl. Leuenbergerioideae, Maihuenioideae, and Opuntioideae), while Cactoideae in its traditional circumscription fell in three distinct groups, where *Blossfeldia* and Cereeae were clearly distinct from the rest of Cactoideae (Fig. 3a). Strikingly, the genetic differentiation between *Blossfeldia* and Cactoideae in the major axis of overall genetic differentiation exceeds that between *Leuenbergeria* and *Pereskia* s.s. (Fig. 3a). All tribes within Cactoideae were also recovered as distinct, non-overlapping clusters in the analysis focussing on Cactoideae (Fig. 3b), with the diverse and species-rich tribes Phyllocacteae, Cereeae, and Cacteae showing considerable genetic heterogeneity.

### Phylogenetic conflict

BUCKy concordance factors across a subset of 28 well-supported gene trees resolved a primary concordance tree with a topology similar to that of the QC analysis (Fig. 4), even though BUCKy accounted for phylogenetic uncertainty within each gene tree. Importantly, the sister relation of *Leuenbergeria* and *Pereskia* s.s. plus the remainder of Cactaceae received medium support (CF 0.437), but support for monophyly was considerably lower (CF 0.155). In addition, *Blossfeldia* was strongly supported as sister to and rather divergent from core Cactoideae (CF 0.652, note the rather long branch separating them), while relations within core Cactoideae remained poorly supported (at most CF 0.303). The primary concordance tree deviated in minor detail from the QC analysis in poorly supported parts of the tree. For instance, Cylindropuntieae as sister to other Opuntioideae received more support (CF 0.509) than alternatives (CF 0.212–0.279), and Copiapoeae received marginally more support as sister to Cactoideae (excluding *Blossfeldia*; CF 0.148) than Copiapoeae plus Lymanbensonieae as sister (0.111). Opuntioideae or Maihuenioideae as sister to Cactoideae received no dependable support (CF 0.019 and 0.003, respectively).

## Discussion

### Phylogenetic resolution

Phylogenomic approaches based on hundreds of loci have in recent years greatly improved understanding of evolutionary relationships amongst Cactaceae and related lineages, for instance based on transcriptome analysis (Wang et al. 2019), whole-chloroplast genomes (Arakaki et al. 2011), multi-copy genes (Moore et al. 2018), custom probe sets (Acha and Majure 2022; Romeiro-Brito et al. 2022, 2023b), or the Angiosperms353 probe set (this study; Acha and Majure 2022). Nevertheless, an overarching conclusion from these phylogenomic studies is that the low amount of sequence divergence and conflicting results found in many earlier Sanger sequencing-based studies (Nyffeler and Eggli 2010a) and in recent studies with very dense taxon sampling but low gene sampling (Thompson et al. 2024) did not disappear with the massive increases in genetic data volume; indeed, Cactaceae phylogenetic reconstruction remains challenging as expected for such a large, young, and species-rich clade. Instead, a phylogenetic pattern of a series of nested, putatively rapid radiations is evident: at the base of Cactaceae (resulting in Cactaceae and Anacampserotaceae plus Portulacaceae; Moore et al. 2018), nested within Cactaceae at the base of Opuntioideae (resulting in three lineages) and at the base of Cactoideae s.s. (seven lineages), and finally within the latter at the base of Cereeae (five lineages; Fig. 2). Importantly, around these nodes, gene concordance factors were generally rather low and resulted in different, typically weakly supported topologies at these positions (Fig. 4, Online Resources 5–8), in line with results based on other phylogenomic probe sets (Acha and Majure 2022), pervasive phylogenetic conflict found in transcriptomic data (Wang et al. 2019), and poor topological support in studies with dense taxon sampling (Thompson et al. 2024). Jointly, these results imply that the orders of magnitude increase in DNA sequencing data could not completely resolve the phylogeny of Cactaceae, suggesting that even more genetic data or species sampling will not result in a completely supported, bifurcating topology either. Rather, our results illustrate the complexity of phylogenetic signals in genomic-scale data.

The idea that the phylogenetic conflict and remaining lack of resolution have biological rather than methodological grounds finds further support in our finding that our four phylogenetic data sets, which differed greatly in size and quality (Table 1) and our genomic concordance analysis all yielded very similar results (Fig. 4, Online Resources 5–8). Specifically, stringent filtering for paralogs (Dataset QC-P) resulted in about 10% less data, but did not affect our results strongly; a minor difference being the position of *Calymmanthium* (Online Resource 6). Moreover, reducing the number of gene trees to only those with reasonable average bootstrap support values resulted in major loss of data (from 299 loci, 273,475 bp aligned length in dataset QC-P, Fig. 2, Online Resource 5; to 15 loci and 30,189 bp aligned length in dataset QC-P-BS50), but with similar overall topological features (Online Resources 6–8). That the normalized quartet score increased from 0.69 to 0.82 with removing poorly supported gene trees reveals that the low amount of sequence divergence in poor-quality loci introduces some phylogenetic noise, but this is not reflected in increased topological support for the ASTRAL species tree (which decreased rather than increased, from 0.88 to 0.78 mean local posterior probability; Table 1). Jointly, analytical details and filtering thresholds did not bring about an overriding improvement in the recovered phylogeny (with some minor exceptions, see results), although we note that overly stringent filtering in fact erodes the phylogenetic signal. Our results thus reflect the usefulness of the Angiosperms353 loci for Cactaceae phylogenetics, confirming the conclusion of other work (Acha and Majure 2022), and forming a sound basis of our classification.

### Topology of early-diverging lineages

The deepest relationships within Cactaceae have long remained contentious. Butterworth and Edwards (2008), Moore et al. (2018), Walker et al. (2018), Wang et al. (2019), Acha and Majure (2022) and our data favour a placement of *Leuenbergeria and Pereskia* s.s. as subsequent sisters to all other cacti, though often with limited support and with a very short branch separating *Leuenbergeria* from *Pereskia* s.s. Alternative topologies (i.e. Opuntioideae or Maihuenioideae as sister to all other cacti) were also sometimes recovered or statistically not significantly less supported (Butterworth and Edwards 2008; Griffith and Porter 2009), though rejected by our data. Generally, studies that include a large number of nuclear gene trees and formal gene tree–species tree reconciliation arrive at Maihuenioideae as sister to Cactoideae s.l. (e.g. Moore et al. 2018, Wang et al. 2019 and Acha and Majure 2022), while others found Maihuenioideae as sister to Opuntioideae plus Cactoideae s.l. (Majure et al. 2023 based on a plastome dataset; Thompson et al. 2024 based on a supermatrix approach including 1063 Cactaceae). Thus, our study is in line with the general trend across phylogenomic studies on the backbone topology of Cactaceae.

It is notable that the early-diverging genera *Maihuenia* (Maihuenioideae), *Maihueniopsis, Pterocactus* (Opuntioideae) and *Blossfeldia* (Blossfeldioideae, sister to Cactoideae) are all small-bodied cushion-forming plants with fleshy taproots and highly mucilaginous cortical and medullary succulent tissue, and all have a similar and partly overlapping geographical range in southern South America along the eastern Andean slopes. In addition, although a great diversity of woodiness exists, Caryophyllales are considered a basically herbaceous group (Kubitzki 1994:318). Therefore, the general notion that the shrubby, highly woody *Pereskia* s.l. represent the ancestral growth form of Cactaceae needs discussion, especially also in the light of the phylogeny of the Portulacineae clade (Nyffeler 2007; Nyffeler and Eggli 2010b; Moore et al. 2018), which identified Anacampserotaceae, Portulacaceae s.s. and Talinaceae as immediate sisters to Cactaceae (with Anacampserotaceae plus Portulacaceae s.s. most likely sister to Cactaceae, though surrounded with phylogenetic conflict, e.g. Moore et al., 2018; Wang et al. 2019). These are all mostly herbaceous, and several with fleshy taproots or root tubers. Jointly, these topological relations suggest the hypothesis that the ancestors of cacti were diminutive, succulent, possibly geophytic plants, while *Leuenbergeria* and *Pereskia* would then have secondarily gained arborescent growth and strongly expressed woodiness, while losing pronounced stem succulence (Griffith 2008:42–43), although Edwards and Donoghue (2006) did not find any indication of secondarily derived woodiness and leafiness. Notably, some species of *Pereskia* s.l. do produce tuberous roots (Leuenberger 1986). The stability of the backbone topology of Cactaceae that our results have added to now should enable more rigorous future work on the ancestral growth form and evolution of woodiness of Cactaceae.

### Implications for classification

A major aim of this work was to present a revised, complete suprageneric phylogenetic classification of Cactaceae based on a novel and informative dataset. Even though we could not include several important lineages due to challenges with molecular work (most importantly, we omitted *Kroenleinia*, *Uebelmannia*, and *Lymanbensonia*; other important omitted lineages are *Miqueliopuntia*, *Strophocactus wittii*, *Rimacactus*, *Leucostele*, *Brachycereus*, *Jasminocereus*, and *Rauhocereus*), our taxon sampling is sufficient for a comprehensive suprageneric reevaluation, covering the vast majority of genera accepted by Korotkova et al. 2021 (close to 90%), plus many putative segregates.

Overall, it is reassuring that the previous comprehensive classification based on interpretation of a wide range of molecular phylogenetic studies, published in the Sanger sequencing era (Nyffeler and Eggli 2010a), is largely congruent with our present phylogenetic results, which are nevertheless much more fine-grained allowing us to place all "incertae sedis" genera in appropriate tribes and/or subtribes.

Importantly, a recurrent topological pattern is one in which a morphologically homogeneous group of species-poor lineages form a grade basal to one or more species-rich clades, for instance the two Pereskioid lineages vs. the rest of Cactaceae (Fig. 2a), the base of Cacteae (Fig. 2b) and the base of Phyllocacteae (Fig. 2c). For classification, this leaves the option of splitting these groups into many morphologically poorly delineated suprageneric taxa, or to accept them as paraphyletic taxa, which is advocated by some (e.g. Hörandl and Stuessy 2010) but firmly rejected by others (e.g. Schmidt-Lebuhn 2012). In general, we aim to only accept monophyletic taxa, but the phylogenetic evidence at the base of Phyllocacteae and Cacteae prompted us to take a pragmatic approach and accept two paraphyletic subtribes (*Echinocactinae and *Corryocactinae), because they are surrounded by phylogenetic conflict, they can be morphologically characterized, and alternative classification scenarios do not find clear evidence and/or would entail not accepting established subtribes. We consistently mark paraphyletic taxa with a * and provide detailed justification in the taxonomic treatment.

Here, we first highlight the most important features of this new classification at each hierarchical level, followed by a detailed taxon-by-taxon discussion in the classification part. Finally, we provide complete genus-level synonymy and assign each to higher taxa (Tables 2–3).

**Table 2:** Accepted higher taxa and their genera presented in a phylogenetic sequence. Paraphyletic taxa are marked with a *.

ID	Subfamily	Tribe	Subtribe	Genera*
*1*	**Leuenbergerioideae**			*Leuenbergeria* Lodé
*2*	**Pereskioideae**			*Pereskia* Mill.
*31*	**Opuntioideae**	**Opuntieae**		*Airampoa* Frič; *Brasiliopuntia* (K.Schum.) A.Berger; *Consolea* Lem.; *Miqueliopuntia* Frič; *Opuntia* Mill.; *Salmonopuntia* P.V.Heath; *Tacinga* Britton & Rose
*32*		**Cylindropuntieae**		*Cylindropuntia* (Engelm.) F.M.Knuth; *Grusonia* F.Rchb. ex Britton & Rose; *Micropuntia* Daston; *Pereskiopsis* Britton & Rose; *Quiabentia* Britton & Rose
*33*		**Pterocacteae**		*Austrocylindropuntia* Backeb.; *Cumulopuntia* F.Ritter; *Maihueniopsis* Speg.; *Pterocactus* K.Schum.; *Tephrocactus* Lem.
*40*	**Maihuenioideae**			*Maihuenia* (Phil. ex F.A.C.Weber) K.Schum.
*50*	**Blossfeldioideae**			*Blossfeldia* Werderm.
*610*	**Cactoideae**	**Lymanbensonieae**		*Calymmanthium* F.Ritter; *Lymanbensonia* Kimnach
*620*		**Copiapoeae**		*Copiapoa* Britton & Rose
*631*		**Cacteae**	*Echinocactinae	*Astrophytum* Lem.; *Aztekium* Boed.; *Echinocactus* Link & Otto; *Geohintonia* Glass & W.A.Fitz Maur.; *Sclerocactus* Britton & Rose
*632*			Ferocactinae	*Ferocactus* Britton & Rose; *Glandulicactus* Backeb.; *Kroenleinia* Lodé; *Leuchtenbergia* Hook.; *Stenocactus* (K.Schum.) A.Berger; *Thelocactus* (K.Schum.) Britton & Rose
*634*			Cactinae	*Acharagma* (N.P.Taylor) Glass; *Ariocarpus* Scheidw.; *Cochemiea* (K.Brandegee) Walton; *Coryphantha* (Engelm.) Lem.; *Cumarinia* (F.M.Knuth) Buxb.; *Epithelantha* F.A.C.Weber ex Britton & Rose; *Escobaria* Britton & Rose; *Kadenicarpus* Doweld; *Lophophora* J.M.Coult.; *Mammillaria* Haw.; *Mammilloydia* Buxb.; *Neolloydia* Britton & Rose; *Obregonia* Frič; *Oehmea* Buxb.; *Ortegocactus* Alexander; *Pediocactus* Britton & Rose; *Pelecyphora* C.Ehrenb.; *Rapicactus* Buxb. & Oehme; *Strombocactus* Britton & Rose; *Turbinicarpus* Buxb. & Backeb.
*641*		**Phyllocacteae**	*Corryocactinae	*Austrocactus* Britton & Rose; *Brachycereus* Britton & Rose; *Corryocactus* Britton & Rose; *Eulychnia* Phil.; *Jasminocereus* Britton & Rose; *Neoraimondia* Britton & Rose; *Pfeiffera* Salm-Dyck; *Strophocactus* Britton & Rose
*642*			Leptocereinae	*Armatocereus* Backeb.; *Castellanosia* Cárdenas; *Leptocereus* (A.Berger) Britton & Rose
*643*			Hylocereinae	*Acanthocereus* (Engelm. ex A.Berger) Britton & Rose; *Aporocactus* Lem.; *Disocactus* Lindl.; *Epiphyllum* Haw.; *Hylocereus* (A.Berger) Britton & Rose; *Kimnachia* S.Arias & N.Korotkova; *Pseudorhipsalis* Britton & Rose; *Selenicereus* (A.Berger) Britton & Rose; *Weberocereus* Britton & Rose
*644*			Echinocereinae	*Backebergia* Bravo; *Bergerocactus* Britton & Rose; *Carnegiea* Britton & Rose; *Cephalocereus* Pfeiff.; *Echinocereus* Engelm.; *Escontria* Rose; *Isolatocereus* (Backeb.) Backeb.; *Lemaireocereus* Britton & Rose; *Lophocereus* (A.Berger) Britton & Rose; *Marginatocereus* Backeb.; *Marshallocereus* Backeb.; *Myrtillocactus* Console; *Nyctocereus* (A.Berger) Britton & Rose; *Pachycereus* (A.Berger) Britton & Rose; *Peniocereus* (A.Berger) Britton & Rose; *Polaskia* Backeb.; *Pterocereus* T.MacDoug. & Miranda; *Stenocereus* (A.Berger) Riccob.
*650*		**Fraileeae**		*Frailea* Britton & Rose
*660*		**Rhipsalideae**		*Hatiora* Britton & Rose; *Lepismium* Pfeiff.; *Rhipsalidopsis* Britton & Rose; *Rhipsalis* Gaertn.; *Schlumbergera* Lem.
*670*		**Notocacteae**		*Eriosyce* Phil.; *Neowerdermannia* Frič; *Parodia* Speg.; *Yavia* R.Kiesling & Piltz
*681*		**Cereeae**	Uebelmanniinae	*Uebelmannia* Buining
*682*			Aylosterinae	*Aylostera* Speg.
*683*			Rebutiinae	*Browningia* Britton & Rose; *Rebutia* K.Schum.; *Weingartia* Werderm.
*684*			Gymnocalyciinae	*Gymnocalycium* Pfeiff. ex Mittler
*685*			Cereinae	*Arrojadoa* Britton & Rose; *Brasilicereus* Backeb.; *Cereus* Mill.; *Cipocereus* F.Ritter; *Coleocephalocereus* Backeb.; *Discocactus* Pfeiff.; *Espostoopsis* Buxb.; *Facheiroa* Britton & Rose; *Floribunda* F.Ritter; *Lagenosocereus* Doweld; *Leocereus* Britton & Rose; *Melocactus* Link & Otto; *Micranthocereus* Backeb.; *Pierrebraunia* Esteves; *Pilosocereus* Byles & G.D.Rowley; *Praecereus* Buxb.; *Siccobaccatus* P.J.Braun & Esteves; *Stephanocereus* A.Berger; *Stetsonia* Britton & Rose; *Xiquexique* Lavor, Calvente & Versieux
*686*			Reicheocactinae	*Reicheocactus* Backeb.
*687*			Trichocereinae	*Acanthocalycium* Backeb.; *Arthrocereus* A.Berger; *Borzicactus* Riccob.; *Cephalocleistocactus* F.Ritter; *Cleistocactus* Lem.; *Cremnocereus* M.Lowry, Winberg & Gut.Romero; *Denmoza* Britton & Rose; *Echinopsis* Zucc.; *Espostoa* Britton & Rose; *Haageocereus* Backeb.; *Harrisia* Britton; *Lasiocereus* F.Ritter; *Leucostele* Backeb.; *Matucana* Britton & Rose; *Mila* Britton & Rose; *Oreocereus* (A.Berger) Riccob.; *Oroya* Britton & Rose; *Pygmaeocereus* H.Johnson & Backeb.; *Rauhocereus* Backeb.; *Samaipaticereus* Cárdenas; *Setiechinopsis* (Backeb.) de Haas; *Vatricania* Backeb.; *Weberbauerocereus* Backeb.; *Winterocereus* Backeb.; *Yungasocereus* F.Ritter

*The following genera are accepted by Korotkova et al. (2021) but not by us for reasons detailed in the Taxonomic treatment: *Chamaecereus, Deamia, Estevesia, Homalocephala, Lobivia, Morangaya, Punotia, Rimacactus, Soehrensia, Trichocereus*. The following genera are accepted by us but not by Korotkova et al. (2021): *Backebergia, Cephalocleistocactus, Floribunda, Glandulicactus, Hylocereus, Lagenosocereus, Mammilloydia, Marginatocereus, Neolloydia, Oehmea, Ortegocactus, Pierrebraunia, Pterocereus, Siccobaccatus, Winterocereus*, though in part updated online on Caryophyllales.org since

**Table 3:**
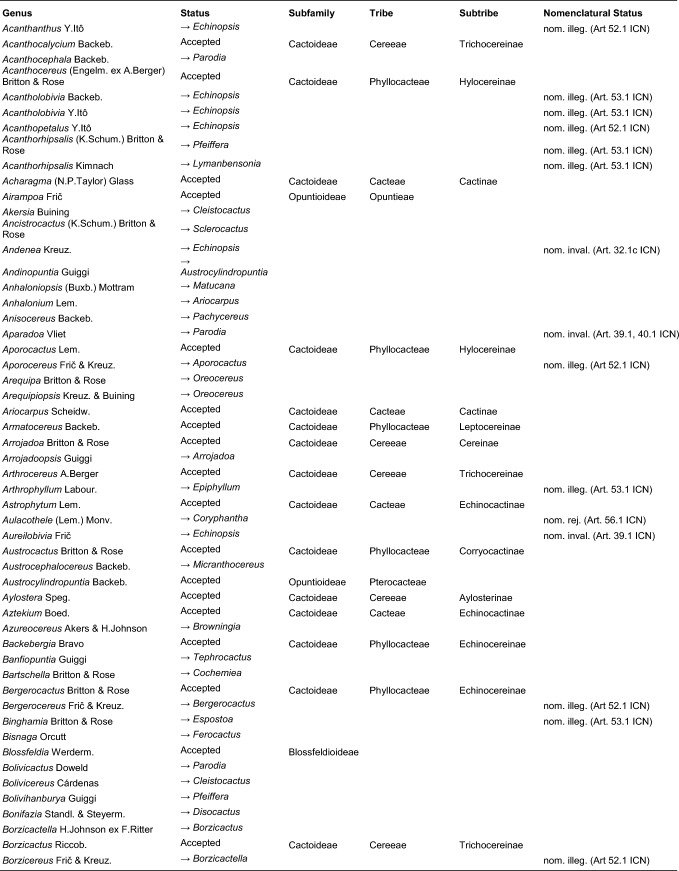

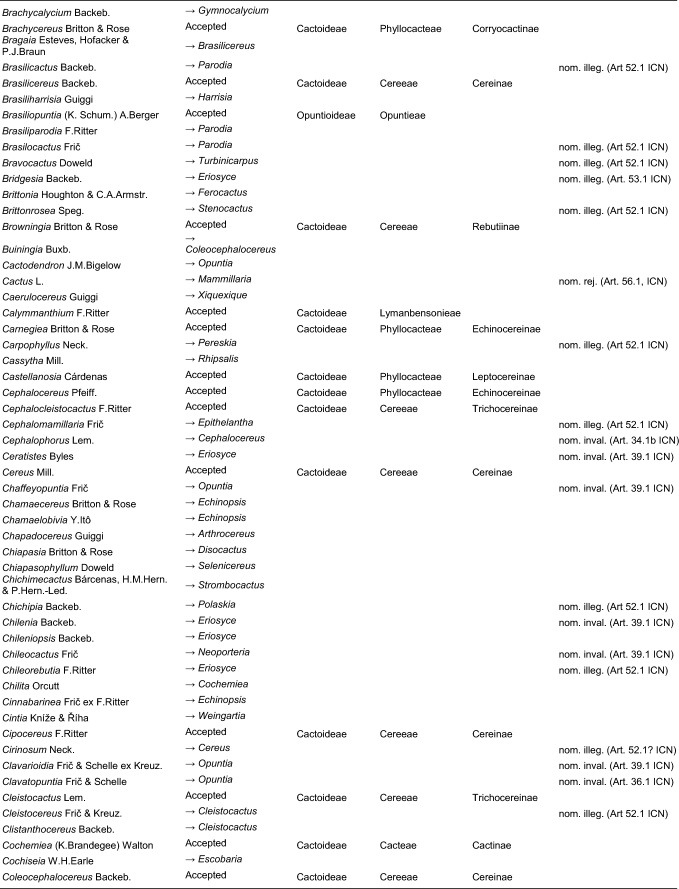

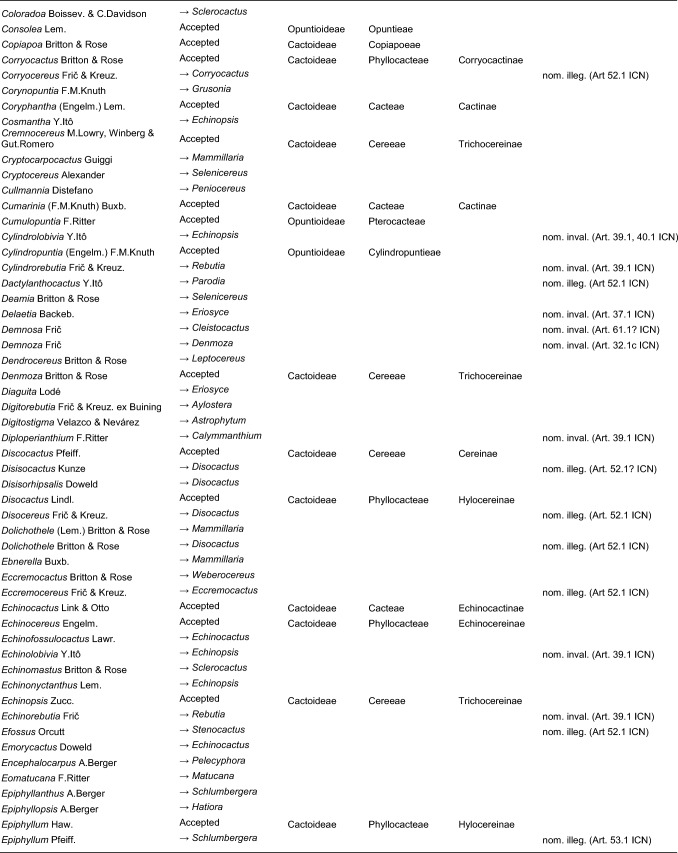

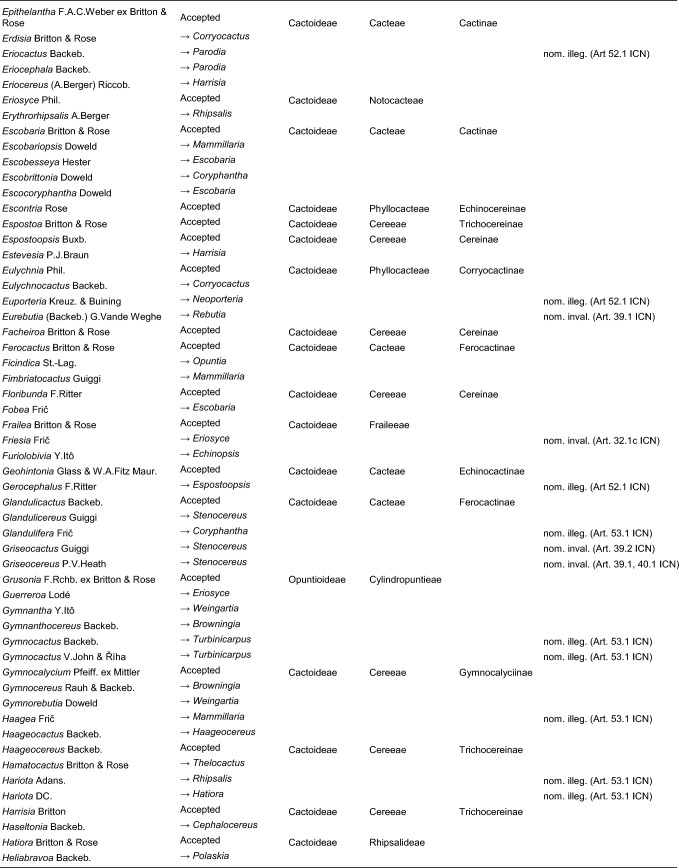

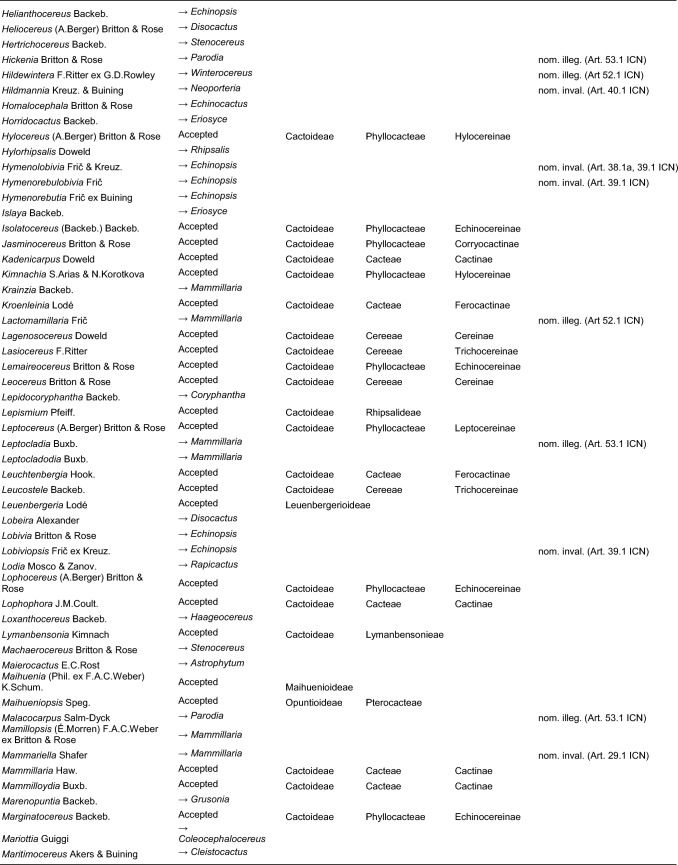

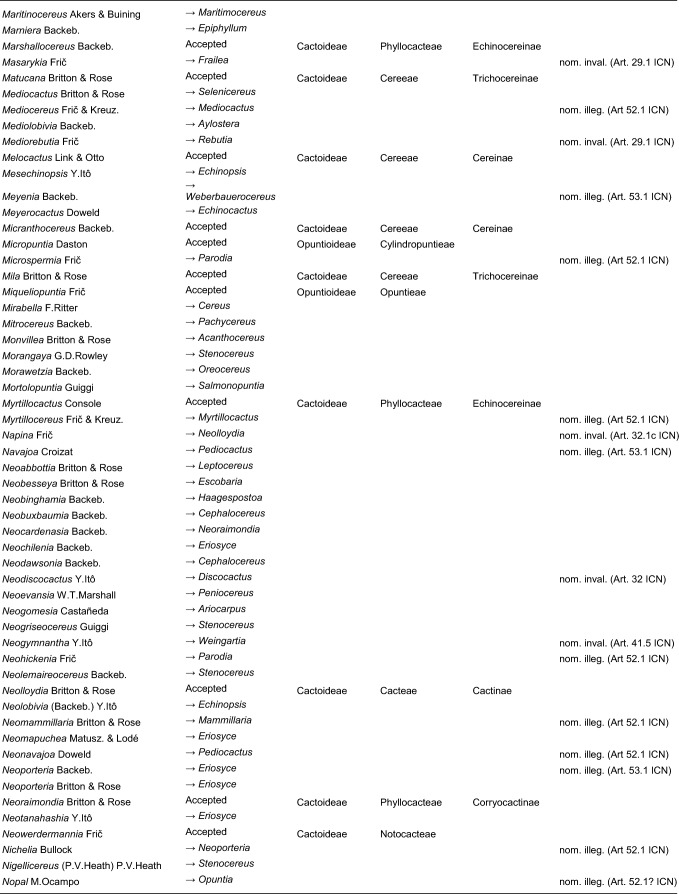

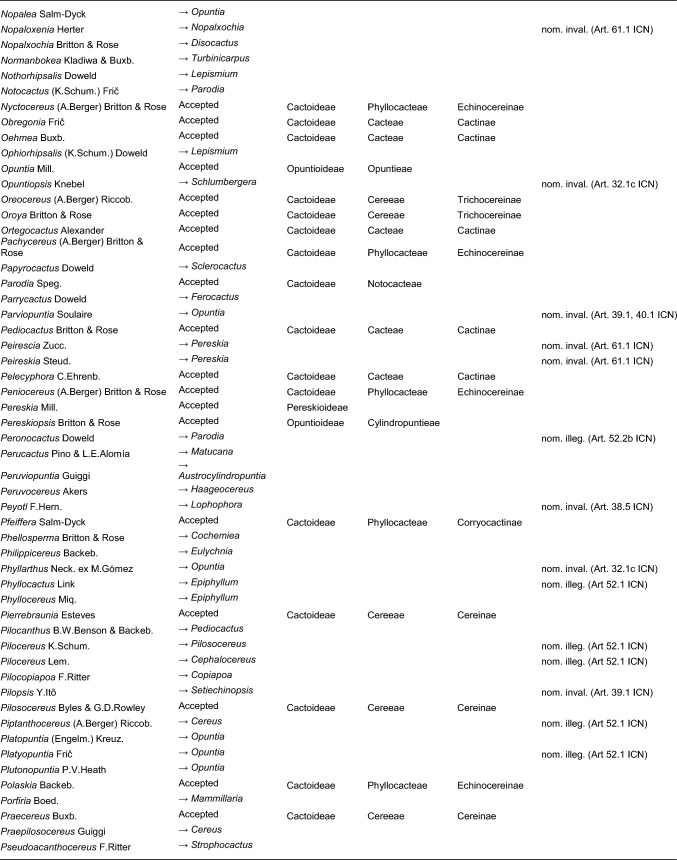

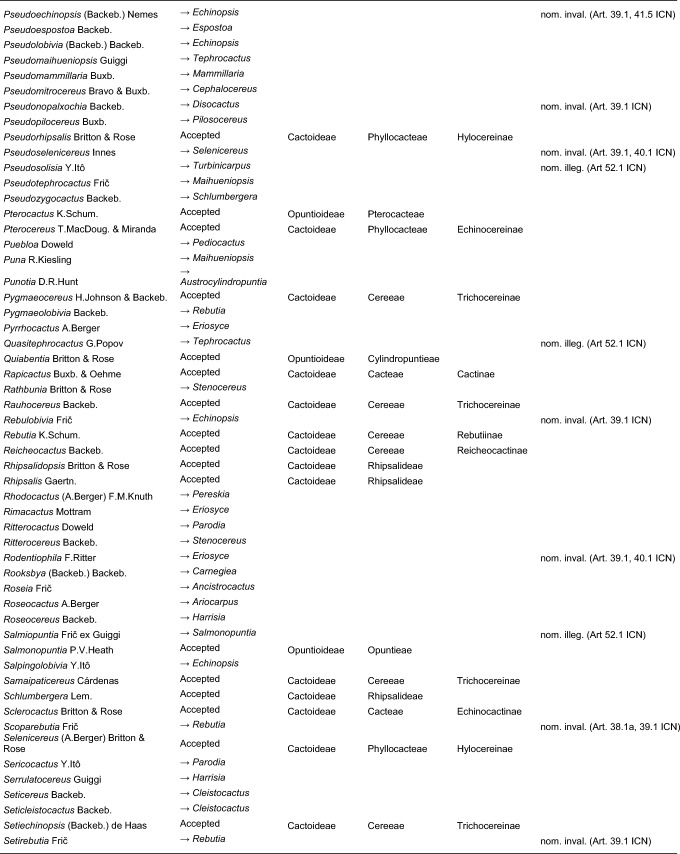

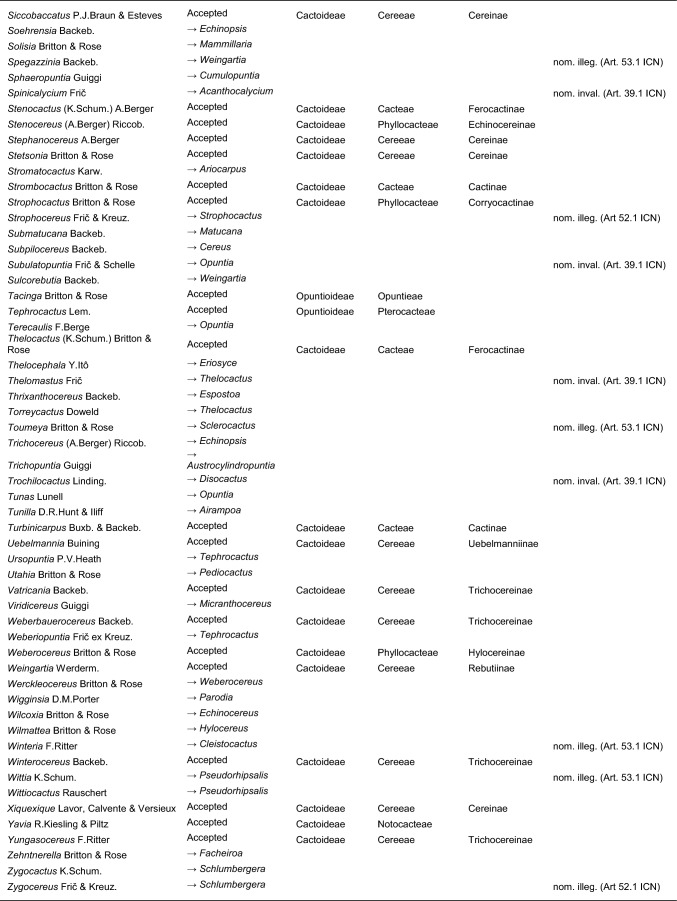
Complete generic synonymy and classification (omitting names for nothogenera, graft chimaerae, and the following obscure, untraceable names listed on IPNI: *Chimerophora* Y.Itô; *Cladoblasia* M.Ocampo; *Dracocactus* Y.Itô; *Tribularia* M.Ocampo)

### Subfamilies

Our results support the recognition of six subfamilies: Pereskioideae in a narrow sense, Leuenbergerioideae, Opuntioideae and Maihuenioideae in their typical circumscriptions, and Cactoideae without *Blossfeldia*, which we instead recognize as a monogeneric subfamily Blossfeldioideae. We propose to split *Pereskioideae in it its traditional wide sense into Pereskioideae in a strict sense and Leuenbergerioideae. Three of our four datasets agree with recent studies based on hundreds of loci that generally recovered *Leuenbergeria* as the sister to all other cacti including *Pereskia* s.s. (e.g. Moore et al. 2018; Walker et al. 2018; Yang et al. 2018; Wang et al. 2019; Acha and Majure 2022). The possible non-monophyly of *Pereskia* s.l. relative to all remaining cacti was first noted by Nyffeler (2002) and confirmed by Butterworth and Wallace (2005) and Edwards et al. (2005). The clade later segregated from *Pereskia* s.s. as *Leuenbergeria* (and treated as monogeneric subfamily Leuenbergerioideae by Mayta and Molinari-Novoa 2015, predominantly Caribbean in occurrence) is primarily characterized by stems with early formation of bark but without stomata, while *Pereskia* s.s. (South America, incl. Andean valleys) has stomata on the stems and the epidermis remains green for a longer time (Leuenberger 1986; Edwards and Donoghue 2006; Ogburn and Edwards 2009; Ocampo and Columbus 2010; Wang et al. 2019). Nevertheless, the species are morphologically homogeneous otherwise, as they can be characterized as terrestrial shrubs to trees with no or very little-pronounced stem succulence with spiniferous areoles representing condensed brachyblasts from the axils of the primary leaves, with conspicuous deciduous sometimes somewhat succulent pinnately veined leaves. Despite the strong molecular affinities of Leuenbergerioideae and Pereskioideae compared to their shared differentiation from other subfamilies based on molecular distance (Fig. 3a), we propose to separate it at subfamily level to draw attention to the likely rapid evolutionary sequence of cladogenesis at the root of the family. This decision is further supported by absence of substantiated evidence for alternative scenarios (Fig. 4). We are confident that this choice best reflects the actual biological relations amongst the species.

A second important decision is splitting Blossfeldioideae from Cactoideae. *Blossfeldia* has first been recognized as representing the sister of the whole remaining diversity of the “true” cacti (Cactoideae) by Nyffeler (2002), rather than being associated with *Parodia* in the highly derived clade Notocacteae, or being the most highly derived of all cacti (Buxbaum 1964; Gorelick 2002). “Nyffeler’s puzzle” was subsequently corroborated by Butterworth (2007), Bárcenas et al. (2011) and Hernández-Hernández et al. (2011), and this isolated position was supported by all subsequent studies. The plants are highly specialized (Barthlott and Porembski 1996): All xeromorphic traits usually associated with cacti are absent, their stems are extremely mucilaginous and “soft” and “gummy” after prolonged dry periods, and stomata are very few and hidden in the areolar crypts. *Blossfeldia* further differs from all Cactoideae by the presence of the rpoCl intron (absent from all Cactoideae; Wallace and Cota 1996, Butterworth 2007), by the absence of cortical bundles and in having an epidermis that is early converted to bark, both characters shared with *Maihuenia* (Mauseth 1999, 2006). Barthlott and Porembski (1996) have suggested that pronounced poikilohydry is present. Placing this monotypic genus in its own monogeneric subfamily is warranted by the unique combination of diagnostic characters relative to all other cactoid cacti and underlines the isolated position of this ancient lineage. Moreover, its phylogenetic position and molecular distinctness are well supported in all our analyses (Fig. 2a, 3a, 4).

### Tribes

While four of the six subfamilies are monogeneric, we follow previous authors in recognizing a series of three and eight morphologically well-characterized tribes for the species-rich subfamilies Opuntioideae and Cactoideae, respectively. Within Opuntioideae, we recognize Pterocacteae, which has nomenclatural priority over Tephrocacteae, which name is otherwise used widespread in recent phylogenetic papers. In our circumscription it includes tribes Austrocylindropuntieae and Tephrocacteae (Doweld 1999; Wallace and Dickie 2002, Anderson 2005), corresponding to Tephrocacteae by Hunt (2011). *Maihueniopsis* is recovered as sister to the remaining genera of the tribe in our preferred analyses (as first proposed by Griffith and Porter 2009; Figs. 2a, Online Resources 5–6), or sister to *Pterocactus* (Online Resources 7–8). Support for these two nodes is low and controversial, but exactly mirrors the results of the more densely sampled analysis of Ritz et al. (2012) as well as the plastome-based analyses of Köhler et al. (2020) and Majure et al. (2023), while Walker et al. (2018) recovered Cylindropuntieae as sister to the rest. Both *Pterocactus* and *Maihueniopsis* were treated as “orphans” by Nyffeler and Eggli (2010a), since the earlier proposal of a monogeneric Pterocacteae and the placement of *Maihueniopsis* (including the monogeneric *Puna* as subgenus) plus *Tephrocactus* in Tephrocacteae suggested by Wallace and Dickie (2002) was based on a poorly resolved phylogeny. Opuntioideae otherwise includes Opuntieae and Cylindropuntieae in well-established circumscriptions.

Within Cactoideae, we recognize eight tribes, but their interrelations remain unclear, as in other studies (e.g. Romeiro-Brito et al. 2022). However, we can now place the three previously orphaned genera of Nyffeler and Eggli (2010a): First, *Calymmanthium*, with its completely unique flower architecture, where the flower proper is completely immersed into a short shoot (pericarpel) with spiniferous areoles, that breaks open at the tip shortly before the expansion of the perianth (Buxbaum 1968, 1969), was shown to be closely related to *Lymanbensonia* (previously assigned to Corryocactinae; Korotkova et al. 2010). They are now jointly placed in Lymanbensonieae. Secondly, the phylogenetic placement of *Copiapoa*, an enigmatic genus with some 30 species, all endemic to the western fringe of the Atacama and the coastline and coastal cordillera from central to northern Chile, was for long discussed controversially. It is shown as sister to all other Cactoideae in some analyses, or as sister to Cactoideae excluding Cacteae in others (e.g. Wang et al. 2019). We here place it in its own monogeneric tribe to underline its isolated but not entirely resolved position as an early-branching lineage in Cactoideae. Thirdly, *Frailea*, some 18 species of terrestrial globose dwarf cacti from low-elevation rocky to sandy grassland habitats in E South America, has long been considered to be an isolated lineage of unclear phylogenetic affinity. Traditionally, the species were thought to be part of Notocacteae, but multiple phylogenetic studies rejected this. In the QC and QC-P data sets (Figs Online Resources 5–6), there is a slight tendency of the genus to be enigmatically associated with Rhipsalideae (as in Romeiro-Brito et al. 2022), but overall it represents one of the seven main phylogenetic lineages of Cactoideae. We therefore place it in its own tribe, Fraileeae.

### Subtribes

To do justice to the immense species and generic diversity of the Cactoideae tribes Cacteae, Phyllocacteae and Cereeae, and their genetic heterogeneity (Fig. 3b), we recognize a total of 14 subtribes in these three tribes. Strikingly, in all three cases a series of species-poor lineages forms a grade to a few more species-rich clades, resembling Angiosperm-wide patterns (e.g. the ANA-grade versus mesangiosperms; basal eudicots versus Pentapetalae, etc.), and the situation at the base of the Cactaceae phylogeny (i.e. Pereskioideae s.l. versus other Cactaceae). In Cacteae, the first three lineages are jointly assigned to *Echinocactinae*,* a paraphyletic grade of three small subclades and sister to the two remaining subtribes. There is unanimous agreement by all studies about the subclade formed by *Geohintonia* + *Aztekium* being sister to all other groups, followed by subclades consisting of on the one hand, *Echinocactus* + *Astrophytum* (incl. *Digitostigma*, Vázquez-Sánchez et al. 2013, Vázquez-Lobo et al. 2016, Vargas-Luna et al. 2018) and on the other hand *Sclerocactus* as sister to a *Ferocactus* clade named Ferocactinae and the large “mammilloid clade” named Cactinae.

In Phyllocacteae, a grade of four small lineages are jointly assigned to a paraphyletic grade *Corryocactinae. These terrestrial or epiphytic diminutive shrubs to tree-like species have stems that are usually segmented, usually have flowers with spiniferous pericarpels, but are ecologically extremely diverse. Compared to the circumscription in Nyffeler and Eggli (2010a), we propose to segregate the clade of *Armatocereus*, *Castellanosia*, *Leptocereus* (incl. *Dendrocereus*) as Leptocereinae subtribe nov. Subtribe Leptocereinae received full support in all analyses, even though it is both morphologically and geographically heterogeneous: While the pericarpels of *Leptocereus* and *Armatocereus* are usually densely spiny because of numerous areoles, those of *Leptocereus* formerly placed in “*Dendrocereus”* are spiny but have rather laxly placed areoles, and those of *Castellanosia* are completely unarmed. Nevertheless, they share the growth form of terrestrial columnar shrubs to trees with richly branched crowns, with segmented ribbed stems and nocturnal flowers. Leptocereinae is strongly supported as sister of the large and heterogeneous sister clades recognized as Hylocereinae and Echinocereinae. Hylocereinae is a clade of terrestrial shrubs with scandent to climbing ribbed stems, or (semi-) epiphytes with scandent, prostrate or pendent stems, predominantly from tropical semi-humid habitats in Mesoamerica, the Caribbean and N South America, and many with very large, nocturnal, sphingophilous flowers. Echinocereinae, although unambiguously monophyletic, contains many nodes that are insufficiently resolved. However again, a basal grade of species-poor lineages as sister to three clades emerges as the branching pattern in Echinocereinae.

Cereeae was recently subjected to a phylogenomic study based on a different set of nuclear markers, the customized Cactaceae591 probe set (Romeiro-Brito et al. 2023b) which includes 13 orthologs from the Angiosperms353 set that we used. In ours as well as their study, support for the early nodes within the tribe is incomplete, but considering all evidence suggests that the evolutionary history is best represented by accepting a grade of monogeneric or genus-poor clades at the rank of subtribes. This contrasts the paraphyletic grade recognized as *Rebutiinae by Nyffeler and Eggli (2010a). In contrast to Romeiro-Brito et al. (2023b), we additionally accept Reicheocactinae. The mono- or ditypic genus *Reicheocactus* from the eastern Andean slopes of La Rioja and San Juan Provinces in central Argentina was for long classified either as *Lobivia* or *Echinopsis*, more recently also as *Rebutia* (Šída 1997). Rather unexpectedly, it was found as sister to all genera of subtribe Trichocereinae by Schlumpberger and Renner (2012) with good support, and our study corroborates this result, again with good support. We suggest placing this enigmatic and likely relictual genus in its own subtribe, to point to its isolated position. Other striking results corroborate those of Romeiro-Brito et al. (2023b), including the recognition of Aylosterinae, Gymnocalyciinae, and a narrower circumscription of Rebutiinae. Both Cereinae and Trichocereinae remain otherwise mostly well-circumscribed with some minor exceptions (see classification part).

## Taxonomic treatment

We present a comprehensive revised classification of the family at subfamilial, tribal and subtribal ranks. Furthermore, we introduce a few informal clade names within several subtribes. In principle, we attempt to accept only monophyletic taxa, except in the two cases where paraphyletic grades are morphologically coherent and genetically homogeneous based on our MDS analyses and there is no clearly defensible alternative; an asterisk (*) is preceding those named paraphyletic grades. For each higher taxon, we attempt to list all included genera (in alphabetical order) and note those that were not part of our sampling (see "Taxon sampling" in the Material and Methods section). We also include major synonyms of accepted genus names, and emphasize changes suggested by our results in comparison to the most recent, previous classifications such as Anderson (2001), Anderson (2005), Hunt et al. (2006), Nyffeler and Eggli (2010a) and the checklist of Hernández-Ledesma et al. (2015). We also incorporate additional insight from recently published molecular studies of minor groups based on comparatively few genes. Where our and other studies remain inconclusive, we are conservative and do not change existing traditional concepts and circumscriptions to conserve stability of names. For published infrafamilial, tribal and subtribal names and their synonyms, we consulted Reveal (2011 +), and for the sake of completeness, we list synonymous infrafamilial names in the appropriate place in chronological order. Botanical authors of genus names are not given in the main text to improve legibility; all data can be found in Tables 2 and 3.

**Leuenbergerioideae** Mayta & Molinari 2015

*Included genus*:

- *Leuenbergeria*

Terrestrial shrubs to trees with no or very little-pronounced stem succulence with spiniferous areoles representing condensed brachyblasts from the axils of the primary leaves, stems without stomata and with early periderm formation and primary epidermis rapidly disappearing, primary leaves conspicuous, deciduous, sometimes somewhat succulent, pinnately veined.

The species here classified were traditionally included in *Pereskia*, and *Leuenbergeria* was only described in 2012 to mirror the results of several molecular phylogenetic studies: The possible non-monophyly of *Pereskia* s.l. relative to all remaining cacti was first noted by Nyffeler (2002) and confirmed by Butterworth and Wallace (2005), Edwards et al. (2005) and Ocampo and Columbus (2010), in phylogenetic analyses based on few phylogenetic loci. Importantly, more recent studies based on hundreds of loci generally recovered the same topology (e.g. Moore et al. 2018; Walker et al. 2018; Yang et al. 2018; Wang et al. 2019; Acha and Majure 2022). The only exception is Majure et al. (2023) which arrived at a monophyletic *Pereskia* s.l., though with very limited statistical support. Typically, all these studies revealed pronounced gene tree-species tree incongruence at these nodes.

*Leuenbergeria* differs from *Pereskia* s.s. in its rapid formation of a periderm that replaces the epidermis and thus stem stomata are absent. Anatomically, most of its species are characterized by isodiametric sclereids in the form of stone cells (except *L. bleo* and *L. lychnidiflora, L. marcanoi* not investigated) (Leuenberger 1986). *Leuenbergeria* is primarily a Caribbean taxon, with the exception of *L. aureiflora* from Brazil.

**Pereskioideae** Engelm. 1876 (as ‘Peirescieae’)

*Included genus*:

- *Pereskia* (incl. *Rhodocactus* [not sampled], excl. *Leuenbergeria*)

Terrestrial shrubs to trees with no or very little-pronounced stem succulence with spiniferous areoles representing condensed brachyblasts from the axils of the primary leaves, stems with stomata and with delayed periderm formation and primary epidermis remaining functional for some time (except *P. aculeata*), primary leaves conspicuous, deciduous, sometimes somewhat succulent, pinnately veined.

In contrast to the clade later segregated as *Leuenbergeria* (and here treated as monogeneric subfamily Leuenbergerioideae, predominantly Caribbean in occurrence) *Pereskia* s.s. is characterized by stems with delayed formation of bark (except *P. aculeata*) and with stomata present (Leuenberger 1986; Edwards and Donoghue 2006; Ogburn and Edwards 2009; Ocampo and Columbus 2010; Wang et al. 2019). *Pereskia* is an entirely South American taxon with marginal overlap in distribution with *Leuenbergeria aureiflora* (Taylor and Zappi 2004).

Interestingly, the data of Edwards et al. (2005) and Butterworth and Wallace (2005) as well as the tree topology in Thompson et al. (2024, supplemental data, though lacking support values) show two separate subclades (SSA clade, Andean clade) in *Pereskia* s.s., each with good support, but the branch combining the two has distinctly lower support. The two clades of *Pereskia* s.s. are congruent with several morphological and anatomical characters (Leuenberger 1986): *Pereskia* s.s., i.e. the Andean clade, have abundant stem stomata (few in *P. aculeata,* which is also geographically separate), do not form brachyblast leaves, and have simple fusiform sclereids and predominantly 3- or 6–9-colpate pollen. Asai and Myata (2015) proposed to recognize the SSA clade as segregate genus *Rhodocactus* that have few stem stomata, produce brachyblast leaves, and have aggregated fusiform sclereids and 12–15-colpate pollen, but given the sparse taxon sampling we tentatively refrain from recognizing this segregate genus.

**Opuntioideae** Burnett 1835 (as ‘Opuntidae’)

Incl. Pereskiopsisoideae Lakomski 2003 (nom. inval.).

Terrestrial dwarf to large shrubs to trees, sometimes geophytic with short-lived shoots; stems segmented, terete or flattened (cladodes), with spiniferous areoles representing condensed brachyblasts in the axils of the primary leaves, primary leaves reduced to terete short-lived rudiments on new growth (flattened parallel-veined slightly to somewhat succulent and long-lived in *Quiabentia* and *Pereskiopsis*); presence of glochids as diagnostic character; fruits umbonate, dry perianth falling off as a whole; seeds comparatively large and with a hard bony cover (except *Pterocactus*).

Our data show Opuntioideae unambiguously as monophyletic clade as did Griffith and Porter (2009), Ritz et al. (2012), Majure et al. (2012), etc. We find good support for three monophyletic subclades that we propose to recognize as tribes, although their interrelations are not entirely clear (compare Figs. 2a and 4ade). This departs from the classification suggested by Wallace and Dickie (2002), which recognized 5 tribes. Nyffeler and Eggli (2010a) accepted only two tribes, but several genera as “incertae sedis”. In comparison with Nyffeler and Eggli (2010a), we re-circumscribe Cylindropuntieae in a narrower sense, and recognize the majority of the South American genera (including all “incertae sedis” of Nyffeler and Eggli (2010a)) as tribe Pterocacteae (this name has nomenclatural priority over Tephrocacteae, which is used in all recent studies)*.* All tribes are almost completely resolved internally, but the grade of early-diverging genera within Pterocacteae reveals some uncertainties. Similar uncertainties concerning the topology of the three tribes were previously found. While the plastome data of Acha and Majure (2022) and Majure et al. (2023) found Opuntieae + (Cylindropuntieae + Pterocacteae), as in our study (Fig. 2a, Online Resources 5–8), the transcriptome data of Acha and Majure (2022) and Walker et al. (2018) recovered Cylindropuntieae + (Opuntieae + Pterocacteae), echoing earlier results (Köhler et al. 2020). In line with this pervasive conflict, the gene concordance analysis using BUCKy (based on sparser taxon and locus representation) revealed that none of these alternatives was particularly strongly supported (CF support ranging 0.21–0.51, Fig. 4ade).

**Opuntieae** DC. 1828

*Included genera*:

- *Brasiliopuntia*
- *Consolea* (not sampled)
- *Opuntia* s.s. (incl. *Nopalea* [not sampled])
- *Miqueliopuntia* (not sampled)
- *Salmonopuntia* (= *Salmiopuntia,* incl. *Mortolopuntia* [not sampled])
- *Tacinga*
- *Airampoa* (= *Tunilla*; see Kiesling et al. (2025), who ascertain that the name *Airampoa* was validly published, and that *Tunilla* has to be treated as synonym)

Terrestrial dwarf shrubs to trees, rarely with a clear definition in trunk and crown, stems segmented, terete or more often flattened cladodes; leaves reduced to short-lived rudiments on new growth only.

The traditionally monotypic South American terete-stemmed genus *Salmonopuntia* resolves as sister to the remaining genera, which fall in two subclades in our data: The first subclade is formed by *Airampoa* (southern American Andes) and *Opuntia* (widespread and spanning almost the entire range of the family from southern Canada to central Argentina), both with flattened stems (cladodes); the second subclade is confined to eastern South America (mainly Brazil) and is composed of *Tacinga* and *Brasiliopuntia*. *Brasiliopuntia* exhibits a highly specialized growth architecture: The plants have a tree-like architecture and produce a terete unsegmented main stem with first- and second-order branches segmented and varying from terete to somewhat flattened, and higher-order branches segmented and consisting of flat and often rather thin cladodes. The type of *Tacinga* (*T. funalis*) has completely terete stems, *T. braunii* is intermediate, and all other species have cladodes. The flowers of both genera possess hairs at the base of the filaments. *Tacinga* has an enigmatic disjunct occurrence in the caatinga of eastern Brazil and in the Caribbean, mirroring similar disjunctions in *Leuenbergeria* (L*. aureiflora* vs. L*. guamacho*) and *Pseudoacanthocereus* s.s. (now *Strophocactus*, see tribe Phyllocacteae subtribe Corryocactinae). A complete picture of this tribe is still to be gained since *Miqueliopuntia* (monotypic, Chile, all stems terete) and *Consolea* (adults with a terete main trunk and branches segmented with cladodes) were not included in our study. *Consolea* has been included in a broad concept of *Opuntia*, e.g. by Nyffeler and Eggli (2010a), but this is clearly erroneous, and the genus is well separated from *Opuntia* s.s. (Majure et al. 2012)—the entirely Caribbean genus *Consolea* consists only of polyploid species, and plastid data firmly place it as sister to the *Tacinga*-*Brasiliopuntia*-*Opuntia* clade (Majure and Puente 2014; Majure et al. 2021, 2023). *Nopalea*, traditionally segregated because of the ornithophilous flower syndrome, has been found deeply nested within *Opuntia* s.s. by Griffith and Porter (2009) and Majure et al. (2012). The placement of *Miqueliopuntia* in Opuntieae was first suggested by Wallace and Dickie (2002), and was corroborated by Griffith and Porter (2009), Hernández-Hernández et al. (2011), Majure et al. (2012), Majure and Puente (2014) and Majure et al. (2023), which all show it as sister to *Tunilla* (which enigmatically clustered with *Maihueniopsis ovata* in the study of Majure et al. (2012)). Köhler et al. (2021) found slightly different topologies of the clades just discussed, and most notably found that the enigmatic *Opuntia schickendantzii* belongs with the hitherto monotypic *Salmonopuntia.* Majure et al. (2012) had included the species in *Brasiliopuntia,* but these authors used cultivated material of unknown origin, and not representing *O. schickendantzii*; the species was recently also segregated as monotypic *Mortolopuntia*.

**Cylindropuntieae** Doweld 1999

Incl. Pereskiopsideae Doweld 1999.

*Included genera*:

- *Cylindropuntia*
- *Grusonia* (incl. *Corynopuntia*, incl. *Marenopuntia*, excl. *Micropuntia*)
- *Micropuntia*
- *Pereskiopsis*
- *Quiabentia* (not sampled)

Terrestrial dwarf to large shrubs, rarely with underground tubers; stems segmented or rarely unsegmented, terete, leaves reduced to terete short-lived rudiments on new growth, or flattened parallel-veined slightly to somewhat succulent and long-lived.

On the basis of our data, the South American genera (excluding the unsampled *Quiabentia*) formerly placed in *Cylindropuntieae* by Nyffeler and Eggli (2010a) are excluded and recognized as separate tribe *Pterocacteae* (q.v.). With this circumscription, *Cylindropuntieae* is an almost completely North American clade with segmented terete stems. The wide concept of *Grusonia* suggested by Wallace and Dickie (2002) and used by, for example, Anderson (2005) is not supported, and the monotypic *Micropuntia* (limited to the Mojave Desert in the southwestern USA) should be separated, as previously suggested by Griffith (2003), Griffith and Porter (2009), Bárcenas (2016) and Majure et al. (2019) and recently confirmed by Majure et al. (2023), while the inclusion of both *Corynopuntia* and *Marenopuntia* in *Grusonia* is supported by most of these studies as well as our data. Enigmatically, *Micropuntia* is found to form a clade with *Pereskiopsis* (Mexico, Guatemala) sister to the remaining genera of the subtribe in our data (corroborating Bárcenas et al. 2011 and Bárcenas 2016). The more deeply sampled studies of Majure et al. (2019) and Majure et al. (2023) confirm this placement and identify *Quiabentia* (South America), *Pereskiopsis* (Mexico, Guatemala) and *Micropuntia* (USA) as consecutive sisters to the remaining Cylindropuntieae*, Grusonia* s.l. + *Cylindropuntia.* The close relationship of these two genera is corroborated by the occurrence of a natural hybrid (× *Cylindronia*, Baker et al. 2023). *Quiabentia* and *Pereskiopsis* are characterized by unsegmented main stems and flattened parallel-veined succulent leaves. It should be noted that these leaves are not homologous to the leaves of *Pereskia* s.l.—while *Pereskia* leaves have the typical architecture with a main rib as midvein and obliquely departing lateral veins, the leaves of *Quiabentia* and *Pereskopsis* show several parallel main veins extending from base to tip, and these leaves are best interpreted as secondarily flattened and enlarged leaf rudiments, as otherwise present in the subfamily. Interestingly, the overall geographical range of *Quiabentia* is very similar to that of *Brasiliopuntia* (tribe Opuntieae), pointing to a likely common biogeographic evolutionary scenario. The position of *Quiabentia* as sister to other Cylindropuntieae was also shown in several earlier studies (Griffith and Porter 2009; Hernández-Hernández et al. 2011; Bárcenas 2016; Majure et al. 2019).

**Pterocacteae** Kuntze 1903

I﻿ncl. Tephrocacteae Doweld 1999; incl. Austrocylindropuntieae R.S.Wallace and S.L.Dickie 2002.

*Included genera*:

- *Austrocylindropuntia* (incl. *Andinopuntia* [not sampled], incl. *Punotia*)
- *Cumulopuntia* (incl. *Sphaeropuntia*)
- *Maihueniopsis* (incl. *Puna* [not sampled])
- *Pterocactus*
- *Tephrocactus* (incl. *Banfiopuntia,* incl. *Pseudotephrocactus* [not sampled])

Terrestrial dwarf shrublets, often geophytic with thickened taproot or succulent root tubers, often forming compact cushions, sometimes stems deciduous, more rarely medium-sized shrubs, stems segmented and terete; leaves reduced to short-live rudiments present on new growth only.

Pterocacteae (this name has nomenclatural priority over Tephrocacteae, which is otherwise used widespread in recent phylogenetic papers) in our circumscription includes tribes Austrocylindropuntieae and Tephrocacteae (Doweld 1999; Wallace and Dickie 2002; Anderson 2005) and was tentatively accepted as Tephrocacteae by Hunt (2011:5). *Maihueniopsis* is shown as sister to the remaining genera of the tribe (as first proposed by Griffith and Porter 2009), followed by *Pterocactus* plus a strongly supported clade embracing all other genera; support for these two nodes is low and controversial, but exactly mirrors the results of the more densely sampled analysis of Ritz et al. (2012) as well as the plastome-based analyses of Köhler et al. (2020) and Majure et al. (2023). Both *Pterocactus* and *Maihueniopsis* were treated as “orphans” by Nyffeler and Eggli (2010a), since the earlier proposal of a monogeneric Pterocacteae and the placement of *Maihueniopsis* (including the monogeneric *Puna* as subgenus) plus *Tephrocactus* in Tephrocacteae suggested by Wallace and Dickie (2002) was based on a poorly resolved phylogeny. *Maihueniopsis ovata* was enigmatically found as sister of a paraphyletic *Airampoa* (= *Tunilla*; subtribe Opuntieae) by Majure et al. (2012), most likely due to a misidentification. A similar problem is the placement of *Cumulopuntia* (analysed species *C. tortispina*) as part of the *Maihueniopsis* clade by Griffith and Porter (2009), since all other cited studies concur with our data and place *Cumulopuntia* as sister to *Austrocylindropuntia*.

The recently segregated monotypic genus *Punotia* is shown as sister to *Austrocylindropuntia* with very good support in our data. This differs from the topology obtained by Ritz et al. (2012), where *Punotia* is shown as sister to *Austrocylindropuntia* + *Cumulopuntia,* but that result was poorly supported and based on limited data (no support from the nuclear phyC locus, bootstrap support 66 and posterior probability 0.91 from the chloroplast matK-trnK locus, no ITS sequence included). Moreover, the close relationship with *Austrocylindropuntia* is in line with the existence of the natural hybrid × *Austronotia* (Sherrah 2020). The monotypic *Banfiopuntia* (only *B. verschaffeltii*, formerly also classified as *Austrocylindropuntia*, e.g. Anderson 2001, 2005) is shown as part of the early-diverging grade of *Tephrocactus* by Ritz et al. (2012) and Las Peñas et al. (2019), and the species separated as *Sphaeropuntia* are part of one of two clades of *Cumulopuntia*.

The former segregate genus *Puna* (type *P. clavarioides*) is sister to the seven further *Maihueniopsis* species analysed by Majure et al. (2023), and recognizing it as a monotypic genus would be defendable in view of its special morphology.

**Maihuenioideae** P.Fearn 1996

*Included genus*:

- *Maihuenia*

Terrestrial dwarf shrubs with fleshy taproot, forming large flat cushions, with strongly mucilaginous succulent somewhat segmented terete stems, with spiniferous areoles representing condensed brachyblasts in the axils of the primary leaves, with conspicuous long-lived terete leaf rudiments.

This subfamily embraces just the two species of *Maihuenia*, confined to southern Argentina and Chile. The tuberous roots are similar to those of *Pereskia* (s.s.) *humboldtii*, the stem epidermis has few stomata and is rapidly converted to bark, and stems and leaves contain substantial mucilage reservoirs, hence interpreted as a “derived southern pereskioid phylad” (Gibson 1977).

**Blossfeldioideae** Crozier 2004

*Included genus*:

- *Blossfeldia*

Terrestrial low-growing solitary flattened small bodies, or offsetting and forming dense low cushions; visible leaves none and primordia reduced to microscopic vestiges, areoles with at most some short inconspicuous felty hairs.

The monotypic genus *Blossfeldia* has first been recognized as representing the sister of the whole remaining diversity of the “true” cacti (Cactoideae) by Nyffeler (2002), rather than being associated with *Parodia* in the highly derived clade Notocacteae or being the evolutionarily most derived of all cacti (Buxbaum 1964; Gorelick 2002). This isolated phylogenetic position was corroborated by Butterworth (2007), Bárcenas et al. 2011, Hernández-Hernández et al. (2011) and all subsequent studies. Placing this monotypic genus in its own monogeneric subfamily is warranted by the unique combination of diagnostic characters relative to all other cactoid cacti, as detailed in the subfamilies chapter above. Notably, the genetic and morphological distinctiveness of Blossfeldioideae is much more pronounced than that of Leuenbergerioideae, the other non-traditional subfamily we propose to accept.

**Cactoideae** Eaton 1836

Incl. Rhipsalidoideae Burnett 1835 (as ‘Rhipsalidae’); incl. Cereoideae Drude 1890, incl. Calymmanthioideae Lakomski 2003 (nom. inval.).

Terrestrial or epiphytic diminutive to dwarf to large shrubs to tree-like with solitary unbranched columns to densely branched crowns, more rarely semi-geophytic with large tuberous main roots, or (hemi-) epiphytes in seasonally humid forested areas; photosynthetic leaves absent, leaf primordia reduced to microscopical vestiges; with spiniferous areoles representing condensed brachyblasts from the axils of the primary leaves; spines various, from hair- or silk-like to heavily subulate, sometimes hooked, rarely completely absent; flowers small to large or sometimes very large (to 40 cm diam.), diurnal or nocturnal, usually with a graded series of numerous perianth elements from sepaloid scales to sepaloid-petaloid intermediates and colourful large petaloid elements; stamens usually many to very many.

These are the "true" cacti, including all globose to columnar taxa lacking conspicuous leaf rudiments. This clade contains the overwhelming majority of genera and species, and includes the entire diversity of cactus growth forms, from tiny unbranched globose stems to iconic trees, and epiphytes. The circumscription of subfamily Cactoideae (traditionally long known under the nomenclaturally incorrect name Cereoideae) has remained uncontested, apart from the traditional inclusion of *Blossfeldia*. On molecular grounds, Cactoideae were first circumscribed on the basis of a unique intron loss in the chloroplast gene rpoCl (Wallace and Cota (1996), *Blossfeldia* not studied, but intron present, see Butterworth (2007)).

The phylogenetic position of the two early-diverging, enigmatic, mono- and bigeneric clades (now formally recognized as tribes Lymanbensonieae and Copiapoeae) has long remained obscure and they were treated as “orphans” by Nyffeler and Eggli (2010a).

**Lymanbensonieae** N.Korotkova & Barthlott 2010

Incl. Calymmantheae ‘R.S.Wallace 2001’, never validly described, but used in several publications such as Anderson (2001), Anderson (2005) or Arias et al. (2005).

*Included genera*:

- *Calymmanthium*
- *Lymanbensonia* (not sampled; incl. *Acanthorhipsalis* p.p.)

Terrestrial straggly shrubs to small unorderly trees to 8 m tall, or epiphytes and then spreading to become pendent; stems flattened or with 3—4 thin wing-like crenate ribs with distantly placed areoles with pungent spination; flowers nocturnal or diurnal, of conventional architecture with rotate perianth in *Lymanbensonia*, but with a completely unique flower architecture in *Calymmanthium*, where the flower proper is completely immersed into a short shoot (pericarpel) with spiniferous areoles, that breaks open at the tip shortly before the expansion of the perianth (Buxbaum 1968, 1969).

Buxbaum (1969) interpreted *Calymmanthium* as one of the most “primitive” genera in the family, and this was corroborated in the species- and gene-poor molecular study of Wallace (2002), who found the genus as sister to all other Cactoideae studied, and this position was also reported by Hernández-Hernández et al. (2011). In contrast, the discoverer of *Calymmanthium* saw it as a specialized, advanced lineage (Ritter 1981). *Lymanbensonia* (previously assigned to Corryocactinae) was included in a study of *Pfeiffera* s.l. (Korotkova et al. 2010) which also investigated species of *Lepismium* and *Acanthorhipsalis*. Korotkova et al. (2010) recovered four species formerly variously treated as *Acanthorhipsalis* or *Pfeiffera* as sister of the monotypic *Calymmanthium*. Hence, they should be placed in the segregate genus *Lymanbensonia*, in line with our results.

**Copiapoeae** Doweld 2001

*Included genus*:

- *Copiapoa* (incl. *Pilocopiapoa* [not sampled])

Terrestrial globose to shortly columnar solitary to cushion-forming shrubs, or some species geophytes with large underground tubers and small above-ground bodies that can be shed under herbivory pressure, stems not segmented; flowers diurnal, in shades of yellow or rarely pinkish, pericarpel scales naked; fruits dry at maturity and opening at the top.

The phylogenetic placement of this enigmatic genus with some 30 species, all endemic to the western fringe of the Atacama and the coastline and coastal Cordillera from central to northern Chile, was for long discussed controversially. Presumably based on sharing the general growth form and a predominance of yellow flowers close to the apex, *Copiapoa* was traditionally placed in Notocacteae (Buxbaum in Endler and Buxbaum 1958; Buxbaum in Endler and Buxbaum 1974; Barthlott and Hunt 1993; Anderson 2001, 2005; Hunt et al. 2006), but all molecular studies (Nyffeler 2002; Bárcenas et al. 2011; Hernández-Hernández et al. 2011, 2014; Wang et al. 2019) agree that it is misplaced in that clade. It is shown as sister to all other Cactoideae in some analyses, or as sister to Cactoideae excluding Cacteae in others (e.g. Wang et al. 2019). Nyffeler and Eggli (2010a) treated it as an unplaced orphan. We here place it in its own monogeneric tribe to underline its isolated but still incompletely resolved position in Cactoideae.

**Cacteae** Rchb. 1832

Incl. Echinocacteae Salm-Dyck 1840 (as *‘*Echinocactoideae’); incl. Mammillarieae K.Schum. 1894.

Terrestrial, globose to shortly columnar solitary to cushion-forming bodies, stems not segmented, with tubercles or more rarely ribs, rarely (semi-) geophytes with enlarged basal underground stem parts or fleshy to tuberous taproots.

This is an almost purely North American (chiefly Mexico and southwestern USA) clade, with only a very few outliers in Central America and northern South America (species of *Mammillaria* such as *M. mammillaris* or *M. columbiana*; *Escobaria cubensis*)—the vast diversity of North American globular cacti is thus the result of single radiation of this clade*.* The position as ancestral lineage in Cactoideae is well-supported also by Walker et al. (2018). The tribe falls into a paraphyletic grade *Echinocactinae as sister to two clades Ferocactinae and Cactinae (together recognized as “core Cacteae” by Vázquez-Sánchez et al. (2013)) in our analysis, but support for part of the topology is low. This pronounced uncertainty as to the topology of this group and the overall very short branch lengths mirror the results of numerous studies of the clade (Butterworth et al. 2002; Butterworth and Wallace 2004; Bárcenas et al. 2011; Hernández-Hernández et al. 2011; Vázquez-Sánchez et al. 2013, 2019; Breslin et al. 2021; Sánchez et al. 2022; Chincoya et al. 2023).

*Echinocactinae Britton & Rose 1922 (as ‘Echinocactanae’)

Incl. Astrophytinae Doweld 2000; incl. Sclerocactinae Doweld 1998.

*Included genera*:

- *Astrophytum* (incl. *Digitostigma* [not sampled])
- *Aztekium*
- *Echinocactus* (incl. *Homalocephala* [not sampled]; excl. *Kroenleinia* [not sampled, see Ferocactinae])
- *Geohintonia*
- *Sclerocactus* (incl. *Ancistrocactus*, incl. *Echinomastus* [not sampled], incl. *Papyrocactus* [not sampled]; excl. *Glandulicactus* [see Ferocactinae])

*Echinocactinae is a paraphyletic grade of three small subclades and sister to the 2 remaining subtribes of tribe Cacteae. Putative options for splitting *Echinocactinae are complicated due to the non-monophyly of several genera, and therefore we refrain from defining named lineages within the grade. There is unanimous agreement by all studies about the clade formed by *Geohintonia* + *Aztekium* being sister to all other groups, followed by clades consisting of on the one hand, *Echinocactus* + *Astrophytum* (incl. *Digitostigma*, Vázquez-Sánchez et al. 2013; Vázquez-Lobo et al. 2016; Vargas-Luna et al. 2018) and on the other hand *Sclerocactus* (incl. *Echinomastus*, not sampled) as sister to a *Ferocactus* clade and the “mammilloid clade”. *Echinocactus* is not monophyletic, and the well-known “Golden Barrel”, *Echinocactus grusonii* (not sampled), is consistently separated from *Echinocactus* s.s.: It clusters with the *Ferocactus* clade (Butterworth et al. 2002; Vázquez-Sánchez et al. 2013; Vargas-Luna et al. 2018) (which is congruent with seed size) and was segregated as monotypic genus *Kroenleinia*. In addition, Vargas-Luna et al. (2018) suggest to segregate also *Homalocephala* since some of their analyses place it as sister to *Astrophytum* + *Echinocactus* s.s. *Sclerocactus* s.l. is also polyphyletic—*Sclerocactus* s.s. (incl. *Ancistrocactus* and *Echinomastus*) is part of our ancestral grade, while *Glandulicactus* is part of the *Ferocactus* clade, as first shown by Hernández-Hernández et al. (2011).

**Ferocactinae** Buxb. 1958

Incl. Thelocactinae Buxb. 1958.

*Included genera*:

- *Ferocactus* (incl. *Bisnaga* [not sampled]; monophyletic only with *Thelocactus* included, but support < 100%)
- *Glandulicactus*
- *Kroenleinia* (not sampled)
- *Leuchtenbergia*
- *Stenocactus*
- *Thelocactus* (incl. *Hamatocactus* [not sampled], incl. *Torreycactus* [not sampled])

This clade is largely characterized by plant bodies with distinct longitudinal ribs (except *Leuchtenbergia* with one-of-a-kind long trigonous tubercles). Our limited sampling shows the two species of *Ferocactus* analysed as grade to *Thelocactus*. The phylogeny presented by Vázquez-Sánchez et al. (2013) is much more detailed and shows rather intricate and in part poorly resolved relationships. In that study, *Ferocactus* was found to be vastly paraphyletic (corroborating Prado et al. 2010; Bárcenas et al. 2011 and Hernández-Hernández et al. 2011) and includes the genera *Glandulicactus*, *Hamatocactus*, *Leuchtenbergia*, *Stenocactus* and *Thelocactus*. The close relationships of these genera are witnessed by several intergeneric hybrids obtained in cultivation, and which have been formally named, e.g. × *Ferobergia*, × *Thelobergia*, × *Leuchtenfera*, × *Ferenocactus*; the clade could thus conveniently be termed the "*Ferocactus* comparium", and uniting all of these genera as *Ferocactus* s.l. should be considered, rather than splitting *Ferocactus* as currently circumscribed into several independent genera.

**Cactinae** Britton & Rose 1920 (as ‘Cactanae’)

Incl. Coryphanthinae Brittton and Rose 1923 (as *‘*Coryphanthanae’), incl. Bravocactinae Doweld 1998, incl. Pediocactinae Doweld 1999, incl. Turbinicarpinae Doweld 1999), incl. Cochemieinae Doweld 2000, incl. Epithelanthinae Doweld 2000, incl. Escobariinae Doweld 2000, incl. Pelecyphorinae Doweld 2000, incl. Mammillariinae Lakomski 2003 (nom. illeg.).

*Included genera*:

- *Acharagma*
- *Ariocarpus* (incl. *Neogomesia*, incl. *Roseocactus* [both not sampled])
- *Cochemiea* (not sampled, incl. *Bartschella* [not sampled], incl. *Chilita* [not sampled], incl. *Phellosperma* [not sampled])
- *Coryphantha* (incl. *Escobrittonia* [not sampled], incl. *Lepidocoryphantha*)
- *Cumarinia* (not sampled)
- *Epithelantha* (not sampled)
- *Escobaria* (not sampled)
- *Kadenicarpus* (not sampled, excluded from *Turbinicarpus* s.s.)
- *Lophophora*
- *Mammillaria* (incl. *Dolichothele* [not sampled], incl. *Escobariopsis* [not sampled], incl. *Leptocladodia* [not sampled], incl. *Mamillopsis* [not sampled], excl. *Mammilloydia,* excl. *Oehmea* [not sampled]; excl. *Cochemiea* [not sampled]). NB: The remaining further segregates of *Mammillaria* have not been sampled as to type by us or by the studies discussed below and we tentatively continue to include them in *Mammillaria*: *Cryptocarpocactus*, *Fimbriatocactus*, *Krainzia*, *Porfiria*, *Pseudomammillaria*, *Solisia*.
- *Mammilloydia*
- *Neolloydia*
- *Obregonia* (not sampled)
- *Oehmea*
- *Ortegocactus* (not sampled)
- *Pediocactus* (not sampled, incl. *Navajoa*, incl. *Puebloa*, incl. *Utahia*)
- *Peleyphora* (incl. *Encephalocarpus* [not sampled])
- *Rapicactus* (incl. *Lodia* [not sampled])
- *Strombocactus* (incl. *Chichimecactus* [not sampled])
- *Turbinicarpus* (incl. *Gymnocactus*, incl. *Normanbokea* [not sampled], excl. *Kadenicarpus* [not sampled], excl. *Rapicactus*).

This is the "mammilloid clade" (a term first used by Butterworth et al. 2002), which includes the majority of the North American radiation of the tribe, characterized by plant bodies with distinct tubercles rather than longitudinal ribs. Our sampling approach does not provide a full picture of the diversity of the clade, but clearly indicates that the genera *Turbinicarpus* s.l., *Coryphantha* and *Mammillaria* s.l., as currently circumscribed, for example, by Anderson (2005) or Hunt et al. (2006) are not monophyletic. This is confirmed by all recent studies of the group, as is the basic division into 3 subclades (Bárcenas et al. 2011; Vázquez-Sánchez et al. 2013; Breslin et al. 2021; Sánchez et al. 2022; Chincoya et al. 2023).

The position of *Epithelantha* differs between the studies: It is placed as sister to the whole “mammilloid clade” by Bárcenas et al. (2011), Vázquez-Sánchez et al. (2013) and Sánchez et al. (2022), or in a near-ancestral position within the clade that includes *Turbinicarpus* s.s. by Vázquez-Sánchez et al. (2019), or as sister to *Turbinicarpus* s.s. by Aquino et al. (2019). The placement of *Pediocactus* (not sampled by us, and so far only a single species (*P. simpsonii*) was ever analysed of its c. 9 species in the concept of Anderson (2005)) is shown as sister to the whole “mammilloid clade” by Vázquez-Sánchez et al. (2019) and Sánchez et al. (2022), but in the light of the vastly differing circumscriptions of *Pediocactus* in the past, this should be regarded as tentative as long as not more species are analysed.

The first subclade sister to the remainder of the “mammilloid clade” is formed by *Ariocarpus*, *Strombocactus* and *Turbinicarpus* s.s. in our phylogeny. According to Vázquez-Sánchez et al. (2013) and confirmed by Vázquez-Sánchez et al. (2019), some species of *Turbinicarpus* should be segregated as *Kadenicarpus* (shown as sister to the other genera of the subclade). According to Bárcenas et al. (2021), the species *Strombocactus corregidorae* should be segregated as monotypic *Chichimecactus*, but the exact topology of the *Ariocarpus–Strombocactus–Turbinicarpus* subclade remains insufficiently supported in this study and the studies of Bárcenas et al. (2021) and Chincoya et al. (2023).

The second subclade is formed by the genera *Lophophora* and *Acharagma* + *Rapicactus*. According to Hernández-Hernández et al. (2011) and Vázquez-Sánchez et al. (2013), *Obregonia* (not sampled by us) also belongs here, as sister of *Lophophora*. The segregation of *Rapicactus* from *Turbinicarpus* s.l. was first supported on molecular grounds by Vázquez-Sánchez et al. (2013) and is proposed on the base of stem anatomical characters (Rosa-Tilapa et al. 2019) as well as different habitat preferences (Donati et al. 2016).

The third subclade includes all remaining genera of the “mammilloid clade”, most notably the large genera *Mammillaria* s.l. and *Coryphantha*, with their dimorphic areoles (i.e. areoles where spine formation and the origin of the flower is spatially separated with or without a connecting groove, in this clade also present in *Escobaria*, *Neolloydia*, *Ortegocactus* and *Pelecyphora* [Butterworth et al. 2002: Fig. 1], as well as in *Ariocarpus* of the first-diverging subclade of Cactinae, and in *Astrophytum* [*Digitostigma*] *caput-medusae* of *Echinocactinae). The non-monophyly of *Mammillaria* s.l. was already found in the early molecular analysis of Butterworth et al. (2002) and was elaborated by Butterworth and Wallace (2004): Most notably, the species of *Mammillaria* subgen. *Cochemiea* clustered with those of *Mammillaria* sect. *Ancistracanthae* and *Mammillaria* sect. *Phellosperma* plus a couple of further species of *Mammillaria* s.s., but also with *Neolloydia* and *Ortegocactus*. This separation of *Cochemiea* s.l. (as sister of a clade that includes *Coryphantha*) from *Mammillaria* s.s. appears well-founded and was confirmed in all subsequent studies (Bárcenas et al. 2011; Hernández-Hernández et al. 2011; Vázquez-Sánchez et al. 2013; Breslin et al. 2021; Sánchez et al. 2022; Chincoya et al. 2023), although the relative positions of *Neolloydia* and *Ortegocactus* varied. *Cumarinia* (a monotypic segregate from *Coryphantha* s.l.) was also placed together with (and usually in ancestral position near the origin of) the clade that includes *Mammillaria* s.s., *Coryphantha* s.l. and *Cochemiea* s.l. (Bárcenas et al. 2011; Vázquez-Sánchez et al. 2013; Breslin et al. 2021; Sánchez et al. 2022; Chincoya et al. 2023; note that these last-mentioned authors wrongly apply the name *Phellosperma* to their lineage 4, but the type of *Phellosperma* is included in their lineage 7). In the most recent approach towards untangling the diversity of the “mammilloid clade”, Sánchez et al. (2022) and Chincoya et al. (2023) found *Neolloydia* and *Ortegocactus* as consecutive sisters of *Cochemiea* s.l., which itself is sister to a clade of *Coryphantha* s.s. + *Escobaria*. *Coryphantha* is only monophyletic when the former mono- or bitypic *Lepidocoryphantha* is excluded. *Escobaria* is only monophyletic when *Lepidocoryphantha* and the morphologically completely different *Pelecyphora* are included (Sánchez et al. 2022; Chincoya et al. 2023; the close relationships between *Lepidocoryphantha* and *Pelecyphora* are also evident in our data), and for reasons of priority, Sánchez et al. (2022) re-circumscribed *Pelecyphora* to include these two elements. Many of the clades in these recent phylogenies are only partially supported, however, and not all type species of the genera of the subtribe have been included in the analyses (most notably, the type of *Mammillaria*, *M. mammillaris*, was never studied prior to our analysis). Uncertainties in the topology are also present in our data, and more species need to be included in molecular phylogenies towards a better understanding of the relative relationships. The overall situation of traditionally recognized genera being vastly polyphyletic, and traditional diagnostic characters appearing to have evolved repeatedly in parallel, approaches the situation in Trichocereinae and the genus *Echinopsis* s.l. (see below). Surprises are likely, as witnessed by the disparate position of *Mammillaria sphacelata* (type of series *Sphacelatae* of *Mammillaria* subgen. *Mammillaria* sect. *Cylindricothelae* according to Lüthy (1995)), not before considered to represent an isolated lineage, but already shown as part of “Clade B” near the base of *Mammillaria* s.s. by Butterworth et al. (2004), while in the phylogeny of Breslin et al. (2021) it is shown as sister to *Coryphantha* s.l. + *Cochemiea* s.l., and as clade together with *Mammillaria* (subgen. *Oehmea*) *beneckei* rather than as part of *Mammillaria* s.s. in the phylogenies of Sánchez et al. (2022) and Chincoya et al. (2023). In the latter phylogeny, *Mammilloydia* + *Mammillaria albiflora*, and *Oehmea* + *Mammillaria sphacelata* are consecutive sisters to *Mammillaria* s.s. Similarly, the inclusion of *Coryphantha macromeris* (the type of the segregate genus *Lepidocoryphantha*) in the study of Sánchez et al. (2022) resulted in a significantly different topology compared to the phylogeny of Breslin et al. (2021). A further enigmatic clade is “Lineage 4” of Chincoya et al. (2023), comprising *Mammillaria theresae*, *M. barbata* and *M. wrightii*, which emerged as sister to *Neolloydia*, *Ortegocactus* and *Cochemiea* s.l.; Breslin et al. (2021) also found *M. wrightii* separate from *Mammillaria*, as sister of *Neolloydia*. In view of these surprising placements, and the often low support values, we regard the drastic approaches of Breslin et al. (2021) (to include *Escobaria* in *Coryphantha* s.l. and to include *Neolloydia* and *Ortegocactus* in *Cochemiea*) and Sánchez et al. (2022) (to include *Escobaria* in *Pelecyphora*) as premature, and propose a classification (see above) that more closely represents the inferred sequence of cladogenetic events in the group.

**Phyllocacteae** Salm-Dyck 1845

Incl. Echinocereeae Buxb. 1958 (as *‘*Echinocereae’), incl. Hylocereeae Buxb. 1958 (as *‘*Hylocereae’), incl. Leptocereeae Buxb. 1958 (as *‘*Leptocereae’), incl. Pachycereeae Buxb. 1958 (as *‘*Pachycereae’), incl. Monvilleeae F.Ritter 1979, incl. Peniocereeae Doweld 2002.

This clade was for long known under the name Hylocereeae Buxb. (1958) (e.g. Anderson 2001, 2005), but Salm-Dyck’s name has priority by far. Although support for some of the early nodes is poor, we accept four separate subtribes to underline the complex relationships in this very diverse group, which is especially notable for numerous independent innovations of epiphytism amongst its species, as well as for high plasticity in growth architecture, ranging from "conventional” ribbed spiny erect stems to pendent leaf-like cladodes, sometimes even amongst species of a single genus (e.g. *Disocactus*, *Pfeiffera*).

**Corryocactinae** Buxb. 1964

Incl. Pfeifferinae Volgin 1986, incl. Eulychniinae Lakomski 2003 (nom. inval.)

*Included genera*:

- *Austrocactus*
- *Brachycereus* (not sampled)
- *Corryocactus* (incl. *Erdisia,* incl*. Eulychnocactus* [not sampled])
- *Eulychnia*
- *Jasminocereus* (not sampled)
- *Neoraimondia* (not sampled, incl. *Neocardenasia* [not sampled])
- *Pfeiffera* (incl. *Acanthorhipsalis* s.s.; incl. *Bolivihanburya* [not sampled], excl. *Lepismium* p.p. → see tribe Rhipsalideae; excl. *Lymanbensonia* [see tribe Lymanbensonieae])
- *Strophocactus* (not sampled, formerly included in *Selenicereus* from subtribe Hylocereinae*, incl. Pseudoacanthocereus* [formerly subtribe Echinocereinae])

Terrestrial or epiphytic diminutive shrubs to tree like, and from some of the most arid Cactaceae habitats along the Chilean coast and in the Patagonian steppes to the perhumid Amazonian forest, stems usually segmented, ribbed and spiny to flat rather thin cladodes, flowers very variable, diurnal to nocturnal, usually with spiniferous pericarpels.

*Pfeiffera*, shown as sister to all other *Phyllocacteae* in our tree, had a convoluted taxonomic history: Traditionally, *Pfeiffera* was restricted to epiphytic or lithophytic *P. ianthothele* and some similar taxa with short ribbed spiny stems. Barthlott and Taylor (1995) included it into a widely circumscribed *Lepismium* (see tribe *Rhipsalideae*), and this concept was followed by Anderson (2001, 2005). Already Nyffeler (2000) showed that this is untenable, and *Pfeiffera* was accepted as genus by Hunt et al. (2006). Korotkova et al. (2010) showed that the genus in this circumscription is still polyphyletic, and that part of its species should be separated as *Lymanbensonia* (see above, tribe Lymanbensonieae), some as *Lepismium*. *Pfeiffera* as now defined is another genus whose species span the morphological continuum from ribbed spiny cereoid stems to flattened cladodia.

The former genus *Pseudoacanthocereus* (2 species, *P. brasiliensis* from northeastern Brazil, and *P. sicariguensis* from northern Venezuela) is notable for its disjunct occurrence. Previously placed in *Acanthocereus*, the two species here classified differ especially in their large pale brown seeds. While *P. brasiliensis* is a ribbed columnar cactus, *P. sicariguensis* often produces flattened cladode-like stems. The seeds of both species are similar, extremely large (to 5 × 3.5 mm), pale brown and very soft—a unique combination of seed characters in the whole tribe. The placement of *Pseudoacanthocereus* as part of the ancestral grade comprising subtribe *Corryocactinae rather than in subtribe Echinocereinae (e.g. Nyffeler and Eggli 2010a) became evident first in the molecular tree of Korotkova et al. (2017), where *Pseudoacanthocereus* is found as sister to *Neoraimondia* (not sampled by us) and the genera of our subtribe Leptocereinae. Enigmatically, Korotkova et al. (2017) found that the type of *Strophocactus* (*S. wittii*) is embedded in *Pseudoacanthocereus*. *Strophocactus* was treated as synonym of *Selenicereus* e.g. by Anderson (2001) and Anderson (2005) but accepted as a genus of three species (*S. wittii*, former *Deamia testudo*, former *Nyctocereus chontalensis*) by Hunt et al. (2006). It provides an excellent example of parallel developments resulting in superficial similarities, since *D. testudo* and *N. chontalensis* are now recognized as bitypic genus *Deamia* of subtribe Echinocereinae. Placing former *Pseudoacanthocereus* in *Strophocactus* (which has nomenclatural priority) appears to be the preferable solution, and *S. wittii* shares flattened cladode-like stems with *P. sicariguensis*. Moreover, *S. wittii* also has rather large (4 × 2 mm) but dark brown seeds (Barthlott et al. 1997).

The placement of the remaining genera in paraphyletic *Corryocactinae follows Nyffeler and Eggli (2010a). The sister-group relationship of *Eulychnia* with *Austrocactus* is consistent with earlier studies (e.g. Nyffeler 2002; Larridon et al. 2018), but only the Chilean *A. spiniflorus* sampled), and Merklinger et al. (2021), analysing an additional five species from Argentina, corroborate this topology, and identified the Chilean narrowly endemic *A. spiniflorus* as sister to all other *Austrocactus* species.

The two species of *Neoraimondia* are probably not congeneric: *N. arequipensis* (*Neoraimondia* s.s.) from the western slopes of the Andes in Peru was included in the study by Nyffeler (2002), where it was shown as part of the Leptocereinae, while Hernández-Hernández et al. (2011) and Korotkova et al. (2017) show *N.* [*Neocardenasia*] *herzogiana* from the eastern slopes of the Andes in Bolivia as sister to *Pseudoacanthocereus*. The two species share the unusual character of many-flowered areoles, and Mauseth and Kiesling (1997) only found minor anatomical differences between them (presence/absence of secondary sclereids and hypodermal crystals).

The placement of *Brachycereus* and *Jasminocereus* here is completely tentative, and they have to our knowledge never been sampled for a molecular study. Barthlott and Hunt (1993) placed both in Trichocereeae, Buxbaum in Endler and Buxbaum (1958) classified *Brachycereus* in Echinocereeae but later (Buxbaum in Endler and Buxbaum 1974) as Hylocereeae, and *Jasminocereus* in Cereeae.

**Leptocereinae** Eggli, Nyffeler & J.M.de Vos, **subtrib**. **nov**.— Type: *Leptocereus* (A.Berger) Britton & Rose.

*Included genera*:

- *Armatocereus*
- *Castellanosia*
- *Leptocereus* (incl. *Dendrocereus*, incl. *Neoabbottia* [not sampled])

*Diagnosis*: Terrestrial, columnar mostly richly branched shrubs to trees; stems segmented, ribbed; flowers nocturnal, pericarpel laxly to densely spiny (but spineless in *Castellanosia* and occasionally seemingly spineless in *Leptocereus* [esp. former *Dendrocereus*]).

*Nomenclatural note*: The name was first used by Lakomski, Swiat Kakt. 38(1–2):68, 2003, but remained invalidly published (ICN Art. 39.2).

This clade, first vaguely appearing in the cladograms of Nyffeler (2002) and Hernández-Hernández et al. (2011) with low support, now emerges from our molecular data with high support. It in part overlaps the unranked “Sippe Leptocerei “ Berger (1929) (including *Leptocereus*, *Acanthocereus* and *Peniocereus*) and tribe Leptocereeae Buxbaum (1958) (a completely artificial assemblage according to present knowledge, including *Armatocereus*, *Corryocactus*, *Eulychnia*, *Facheiroa*, *Leocereus*, *Neoraimondia* and *Samaipaticereus*). Subtribe Leptocereinae is both morphologically and geographically heterogeneous: While the pericarpels of *Leptocereus* and *Armatocereus* are usually densely spiny because of numerous areoles, those of the former segregate *Dendrocereus* are spiny but have rather laxly placed areoles, and those of *Castellanosia* are completely unarmed. Geographically, the occurrence of the three genera is non-contiguous: The monotypic *Castellanosia* (formerly included in *Browningia*, e.g. by Anderson (2001) and Anderson (2005), but accepted by Hunt et al. (2006) on the base of Nyffeler (2002)) inhabits the lowlands of Bolivia, the species of *Armatocereus* hail from northwestern South America (Colombia, Ecuador, Peru), and *Leptocereus* is an inhabitant of the Caribbean. *Dendrocereus* was shown to be embedded in *Leptocereus* by Barrios et al. (2020).

**Hylocereinae** Britton & Rose 1920 (as ‘Hylocereanae’)

Incl. Epiphyllinae Britton and Rose 1923, incl. Disocactinae Buxb. 1958, incl. Heliocereinae Bravo 1962 (nom. inval.), incl. Weberocereinae Doweld 2002, incl. Acanthocereinae Lakomski 2003 (nom. inval.).

*Included genera*:

- *Acanthocereus* (incl. *Monvillea* [not sampled], incl. *Peniocereus* subgen. *Pseudoacanthocereus*)
- *Aporocactus*
- *Disocactus* (excl. *Aporocactus*, incl. *Bonifazia* [not sampled], incl. *Chiapasia* [not sampled], incl. *Nopalxochia* [not sampled])
- *Epiphyllum* (incl. *Marniera* [not sampled])
- *Hylocereus* (incl. *Wilmattea* [not sampled])
- *Kimnachia* (?, not sampled)
- *Pseudorhipsalis* (not sampled, incl. *Wittiocactus* = *Wittia* [not sampled])
- *Selenicereus* (incl. *Cryptocereus* [not sampled], incl. *Werckleocereus* [not sampled], excl. *Deamia* [see subtribe *Echinocereinae*], excl. *Strophocactus* [see subtribe *Corryocactinae])
- *Weberocereus* (incl. *Eccremocactus* [not sampled], excl. *Werckleocereus* [a synonym of *Selenicereus*])

Terrestrial shrubs with scandent to climbing ribbed stems, or (semi-) epiphytes with scandent, prostrate or pendent stems, stems segmented or not, ribbed and spiny or flattened cladodes with or without spines, predominantly from tropical semi-humid habitats in Mesoamerica, the Caribbean and N South America, and many with nocturnal often very large flowers, usually sphingophilous.

Growth form diversity in this subtribe is stupendous, and there are all transitions from “conventional” many-ribbed cereoid growth (e.g. *Acanthocereus* spp., *Aporocactus* ssp., *Selenicereus* spp.) to few-ribbed cereoid shrubs and scandent lianas (e.g. *Hylocereus*) to flat-stemmed shrubs (e.g. *Weberocereus frohningiorum*, *Epiphyllum* spp., *Disocactus* p.p.). The subtribe is also renowned for the ease of intergeneric hybridization in cultivation (resulting in the "epicacti" of the trade, involving crosses with parents from three and more genera), and the Hylocereinae (except *Acanthocereus*) could be termed the "*Epiphyllum* comparium", with several dozen names published for various hybrid combinations of the main genera and the numerous segregates.

The topology in our data is not entirely resolved, especially involving the subclade that includes *Hylocereus* and *Selenicereus* (the “hylocereoid clade” of Korotkova et al. (2017)). These two genera were traditionally separated by pericarpel and fruit characters—the pericarpels and fruits of *Hylocereus* are scaly and devoid of spines, and those of *Selenicereus* are without scales and more or less distinctly spiny. The very limited sampling of Hernández-Hernández et al. (2011) shows *Hylocereus* and *Selenicereus* as distinct monophyletic sister groups, and Cruz et al. (2016) found them in an unresolved polytomy, while the more extensively sampled studies of Plume et al. (2013) and especially the almost complete sampling of Korotkova et al. (2017) identifies *Selenicereus* (incl. the species of *Weberocereus* formerly segregated as *Werckleocereus*, but excluding *Deamia*) as paraphyletic grade relative to a terminal clade of *Hylocereus* species, and they propose to unite all of them in a widely circumscribed *Selenicereus* s.l. (but excluding *Deamia*), a solution which we feel is premature in the light of the available sequence data. The exclusion of *Deamia* is, however, also supported by our data, showing it unequivocally as part of the basal grade of Echinocereinae.

*Weberocereus* s.s. (excl. *Werckleocereus* as detailed above) is a rather well-supported monophyletic clade in the analysis of Korotkova et al. (2017), which also includes *Weberocereus frohningiorum*, which we included in our study.

*Pseudorhipsalis* was placed as sister to *Epiphyllum* s.s. by Cruz et al. (2016), while Korotkova et al. (2017) found *Pseudorhipsalis* to be polyphyletic—the majority of species is placed as sister to *Epiphyllum* + *Disocactus*, while *P. ramulosa* (segregated as new monotypic genus *Kimnachia*, not sampled by us) is shown in a polytomy with *Epiphyllum* + *Disocactus*.

*Disocactus* is one of long-standing “problem areas” in the classification of the epiphytic cacti: The broad circumscription used by, for example, by Anderson (2001, 2005) and Hunt et al. (2006) goes back to Barthlott (1991), who included several genera formerly recognized as segregates (i.e. *Aporocactus*, *Heliocereus*, *Nopalxochia*) usually on the base of a combination of stem and flower characters. In this wide circumscription, the genus embraced the whole continuum from cereoid many-ribbed spiny stems to flattened cladodes with or without spines, all usually growing as epiphytes. Cruz et al. (2016), Korotkova et al. (2017) and Rosas-Reinhold et al. (2022) recently questioned the monophyly of this widely circumscribed *Disocactus* s.l.: On the one hand, they found the former *Aporocactus* as separate clade, either as sister to a *Hylocereus*-*Selenicereus*-*Weberocereus* clade (Cruz et al. 2016; Rosas-Reinhold et al. 2022) or in an unresolved position in a polytomy with *Acanthocereus* and the remaining *Hylocereinae* (Korotkova et al. 2017). Both Cruz et al. (2016) and Korotkova et al. (2017) also found that some species of *Epiphyllum* (e.g. the well-known *E. crenatum*) cluster with *Disocactus* (excl. *Aporocactus*), further complicating diagnostic morphology-based circumscriptions of the genera of Hylocereinae. However, morphoanatomical stem characters such as the presence or absence of crystal druses or cuticle thickness alone or in combination present synapomorphies for all of these genera except *Selenicereus* s.s. and *Weberocereus* (Martínez-Quezada et al. 2020). Subtribe Hylocereinae is notable for the parallel evolution of incised stems reminiscent of fern fronds in *Epiphyllum chrysocardium*, *Weberocereus imitans*, *Disocactus* (formerly *Epiphyllum*) *anguliger* and *Selenicereus anthonyanus* (see Fig. 3 in Korotkova et al. 2017).

*Acanthocereus* was formerly placed in *Corryocactinae*, but our phylogeny shows it as sister to the remaining Hylocereinae with good support, corroborating the results of Korotkova et al. (2017). *Peniocereus* has been shown to be polyphyletic by Arias et al. (2005), and subgen. *Pseuodacanthocereus* (represented by *P. cuixmalensis* in our analysis) is part of a clade that corresponds to *Acanthocereus*, and this is supported by our data.

Our results are largely congruent with the findings of the above-cited individual studies, with the exception that *Epiphyllum* and *Pseudorhipsalis* s.s. (represented by *P. horichii*) are shown as consecutive sisters to *Disocactus* + *Aporocactus* with maximum support.

**Echinocereinae** Britton and Rose 1922 (as ‘Echinocereanae’)

Incl. Nyctocereinae Buxb. 1958, incl. Cephalocereinae Buxb. 1961, incl. Myrtillocactinae Buxb. 1961, incl. Pachycereinae Buxb. 1961, incl. Pterocereinae Buxb. 1961, incl. Stenocereinae Buxb. 1961, incl. Selenicereinae Lakomski 2003 (nom. inval.).

Terrestrial many-ribbed dwarf to medium-sized shrubs to iconic candelabriform branched trees and solitary columns, or rarely (semi-) epiphytes with few-ribbed scandent stems rooting along their length (*Deamia*), stems usually unsegmented, many with large nocturnal flowers, often chiropterophilous.

This is an almost completely North and Central American group of columnar cacti, with a centre of diversity in Mexico. Plant size ranges from diminutive *Echinocereus* spp. to massive arborescent iconic trees of *Cephalocereus*, *Pachycereus* and *Carnegiea*, etc. *Pachycereus* s.l., *Peniocereus* s.l. and *Stenocereus* s.l. are all clearly polyphyletic in our tree, sometimes spanning more than one clade (see below for details). Overall, our topology differs quite substantially from that in the most recent study by Franco-Estrada et al. (2021), esp. with regard to the position of *Nyctocereus*, but many of the subclades are similar. Our understanding of this subtribe remains sketchy and largely parallels the situation of subtribe Cactinae in tribe Cacteae: Echinocereinae is unambiguously monophyletic, but many nodes in the internal topology are insufficiently resolved, and our knowledge of the diversification history of the clade remains incomplete, inter alia because the sampling of the individual studies overlaps only partially. On the base of our data, a grade of species-poor lineages as sister to three clades emerges, but several nodes are insufficiently supported, and we therefore prefer to leave these entities without formal names:

- Grade of early-diverging lineages: *Included genera*:
  - *Deamia* (formerly *Selenicereus* of subtribe Hylocereinae)
  - *Echinocereus* s.s. (excl. *Morangaya* [see Clade 1], incl. *Wilcoxia* [not sampled])
  - *Lemaireocereus*
  - *Nyctocereus* (formerly *Peniocereus*)
  - *Peniocereus* s.s. (not sampled*; incl. *Cullmannia* [not sampled], incl. *Neoevansia* [not sampled]; excl. *Nyctocereus*, excl. subgen. *Pseudoacanthocereus*, whose species are now included in *Acanthocereus* of the Hylocereinae).

*: We sampled *Peniocereus* (subgen. *Pseudoacanthocereus*) *cuixmalensis*, which is now placed in *Acanthocereus*, and *Peniocereus* (*Nyctocereus*) *serpentinus*, which is now accepted as monotypic *Nyctocereus*. *Deamia*: The genus was treated as synonym of *Selenicereus* by Anderson (2001, 2005) and as synonym of *Strophocactus* by Hunt et al. (2006) (see Hylocereinae above for these genera), but clearly resolves as sister to the remaining three clades in our data, corroborating Korotkova et al. (2017) and Franco-Estrada et al. (2021).

*Echinocereus*: The genus is monophyletic only when *E. pensilis* is excluded (see below, Clade 2), while *Wilcoxia* is clearly embedded (Hernández-Hernández et al. 2011; Sánchez et al. 2014, 2018). *Echinocereus* is notable for the flower buds that burst through the epidermis in almost all species (see Sánchez et al. (2015) for details).

*Peniocereus* s.s. (incl. *Cullmannia* and *Neoevansia*, none included in our study) is placed here on account of the phylogeny of Franco-Estrada et al. (2021), where it is shown as sister to *Nyctocereus* plus their “*Pachycereus* group” (equals roughly our Clade 3 below). In our analysis, *Nyctocereus* resolves as sister to all other *Echinocereinae*. The polyphyletic nature of *Peniocereus* s.l. was first noted by Bárcenas et al. (2011).

*Lemaireocereus* was formerly included in *Pachycereus* s.l. The isolated position of this entity as sister to the remaining North American columnar cacti was first noted by Arias et al. (2003) and Arias and Terrazas (2006), and this is corroborated by our data and that of Franco-Estrada et al. (2021).

- Clade 1: *Included genera:*
  - *Escontria*
  - *Isolatocereus*
  - *Myrtillocactus*
  - *Polaskia* (incl. *Heliabravoa* [not sampled])
  - *Stenocereus* (not sampled; incl. *Griseocereus* [not sampled], incl. *Hertrichocereus* [not sampled], incl. *Machaerocereus* [not sampled], excl. *Marshallocereus* (see Clade 2), incl. *Morangaya* (formerly included in *Echinocereus*), incl. *Rathbunia* [not sampled], incl. *Ritterocereus*).

*Morangaya*: The correct placement of the former *Echinocereus pensilis* with presumably ornithophilous flowers from Baja California (Mexico) is still a matter of discussion: It is nested within a clade that includes *Stenocereus*, *Escontria*, *Myrtillocactus* and *Polaskia* (the “*Stenocereus* group” of Sánchez et al. 2014) in Prado et al. (2010), Bárcenas et al. (2011) and our data, while Sánchez et al. (2014) and Franco-Estrada et al. (2021) found it as sister to this group. Sánchez et al. (2018) show *Morangaya* as sister to *Stenocereus* (*Rathbunia*) *alamosensis*, with which it shares flowers with the ornithophilous syndrome and fruit characteristics (Lange 2016). In our phylogeny, accepting *Morangaya* would make *Stenocereus* paraphyletic, or *Ritterocereus* would have to be accepted as a further segregate. A new combination is needed to formalize the position of *Echinocereus* (*Morangaya*) *pensilis* in our phylogeny by placing it in *Stenocereus*.

*Stenocereus pensilis* (K.Brandegee) Eggli, Nyffeler and J.M.de Vos, **comb. nov.** Basionym: *Cereus pensilis* K.Brandegee, Zoe 5(10):192, 1904. Other names based on the same basionym: *Echinocereus pensilis* (K.Brandegee) J.A.Purpus, Monatsschr. Kakteenk. 18:5, 1908; *Morangaya pensilis* (K.Brandegee) G.D.Rowley, Ashingtonia 1(4):44, 1974.

For *Polaskia*, we included its type, *P. chichipe*, in our study, and this was placed as sister to *Myrtillocactus* with good support, mirroring the results of Franco-Estrada et al. (2021). The second species of the genus, *P. chende* (not sampled, also treated as segregate genus *Heliabravoa*), is shown as sister to *Escontria* by Franco-Estrada et al. (2021). The close relationship of *Escontria* and *Polaskia* is also mirrored by the existence of the natural hybrid × *Polascontria* (Cruz-Zamora et al. 2017).

*Stenocereus* in its wide circumscription as employed by Anderson (2001, 2005) or Hunt et al. (2006) is a Gordian knot since long, with a multitude of segregates described over time. Unfortunately, the type, *S. stellatus*, was not included in our analysis, but it and *S. thurberi*, which we analysed, are both shown as part of a para- and polyphyletic *Stenocereus* by Arias et al. (2005). The disparate placements of the segregates of *Stenocereus* s.l. we analysed (*Isolatocereus*, *Marshallocereus*, *Ritterocereus*) make it clear that the concept of *Stenocereus* s.l. (as circumscribed by Anderson (2001, 2005) or Hernández-Ledesma et al. (2015)) is vastly polyphyletic and thus untenable, and *Marshallocereus* is not even part of this clade but belongs to Clade 2, as also found by Franco-Estrada et al. (2021), and *S. yunckeri* likewise does belong there (Majure et al. 2023). A solution is not yet in sight, and our list above of genera to recognize for the clade is tentative.

- Clade 2: *Included genera:*
  - *Backebergia* (formerly *Pachycereus*)
  - *Bergerocactus*
  - *Carnegiea*
  - *Cephalocereus* (incl. *Neobuxbaumia,* incl. *Neodawsonia* [not sampled], incl*. Pseudomitrocereus* [not sampled])
  - *Lophocereus* (formerly *Pachycereus*)
  - *Marginatocereus* (formerly *Pachycereus*)
  - *Marshallocereus* (formerly *Stenocereus* of Clade 1)
  - *Pachycereus* (*Pachycereus* s.s. not sampled; excl. *Lemaireocereus* [part of the grade of early-diverging lineages], excl. *Lophocereus*, excl. *Marginatocereus*, excl. *Marshallocereus*, excl. *Pterocereus*)
  - *Pterocereus* [not sampled]

*Neobuxbaumia* (incl. *Pseudomitrocereus* [as *Mitrocereus fulviceps*], Hernández-Hernández et al. (2011)) is shown as sister of *Cephalocereus* in our tree, and this is congruent with earlier studies: Arias et al. (2003), Arias et al. (2005), Arias and Terrazas (2006), Bárcenas et al. (2011), Hernández-Hernández et al. (2011), Tapia et al. (2017) and Franco-Estrada et al. (2021) all found species of *Cephalocereus* and *Neobuxbaumia* mixed in a monophyletic clade, and accordingly, *Neobuxbaumia* should therefore be included into *Cephalocereus* (Tapia et al. 2017). The placement of *Cephalocereus* s.l. (central Mexico) as sister to *Bergerocactus* (southwestern-most California and northwestern Baja California) in our tree is biogeographically enigmatic, but was also found by Franco-Estrada et al. (2021).

*Pachycereus* s.l.: This subclade is largely composed of segregates from *Pachycereus* s.l. plus *Marshallocereus* (formerly *Stenocereus*) plus the monotypic *Carnegiea*, and our topology is congruent with the more deeply sampled analyses of Arias et al. (2003), Arias et al. (2005), Arias and Terrazas (2009), and Franco-Estrada et al. (2021). A conservative approach on the basis of this evidence would be to recognize *Pachycereus* in a wide sense, including the monotypic genus *Carnegiea*, which has nomenclatural priority. However, recognizing several segregates (*Backebergia* as sister to *Pterocereus* + *Lophocereus*, and *Carnegiea* as sister to *Pachycereus* s.s. according to the molecular tree of Franco-Estrada et al. 2021) would more cogently describe the evolutionary diversification of this terminal clade, especially the occurrence of cephalia or cephalium-like structures in *Backebergia* and *Lophocereus*. Recognizing these segregates at generic level was also proposed by Arias and Terrazas (2009) and Franco-Estrada et al. (2021).

**Fraileeae** B.P.R.Chéron 2016

*Included genus*:

- *Frailea*

Terrestrial globose dwarf plants from low-elevation rocky to sandy grassland habitats in E South America (S Brazil, Uruguay, Paraguay, Bolivia, N Argentina), bodies offsetting or not, usually tuberculate, unsegmented; flowers from near the apex, in shades of pale yellow, pericarpel scale axils with wool and soft bristles.

The isolated position of *Frailea* has been known for some time, and it was treated as an orphan by Nyffeler and Eggli (2010a). Its position in our phylogeny as sister to tribe Rhipsalideae is most enigmatic, and support for this topology is limited. In fact, the branch that unites Fraileeae with Rhipsalideae is very short and poorly supported.

**Rhipsalideae** DC. 1828

*Included genera*:

- *Hatiora* (excl. *Rhipsalidopsis*, see below)
- *Lepismium* s.s. (excl. *Acanthorhipsalis* p.p. [see tribe Lymanbensonieae], excl. *Pfeiffera* [see tribe Phyllocacteae, subtribe Corryocactinae)]
- *Rhipsalidopsis*
- *Rhipsalis* (incl. *Erythrorhipsalis* [not sampled])
- *Schlumbergera* (incl. *Pseudozygocactus* [not sampled], incl. *Zygocactus* [not sampled])

Epiphytic or rarely lithophytic dwarf shrubs with spreading to pendent segmented stems, stems terete or flat cladodes, areoles sometimes combined into a group of several apical areoles, or absent; spination weak and sometimes reduced to insignificant wool, flowers diurnal.

A neatly circumscribed monophyletic clade (Calvente et al. 2011a, 2011b; Korotkova et al. 2011) for five epiphytic (or rarely lithophytic) genera mainly from South America, with only *Rhipsalis* extending to the North, and also to Africa, Madagascar and Sri Lanka (*R. baccifera*, the only cactus naturally occurring in the Old World). *Hatiora* s.l. (as circumscribed by Barthlott and Taylor (1995) and, for example, used by Anderson (2001) or Anderson (2005)) is not monophyletic in our analysis, as previously found by Calvente et al. (2011a) and Korotkova et al. (2011), and *Rhipsalidopsis* ("Easter Cactus") should be recognized as distinct for species with flat cladodes and red to pink flowers, while *Hatiora* embraces species with terete and often club-shaped stems and yellow or pink flowers. *Hatiora epiphylloides*, also at some time segregated as *Pseudozygocactus*, was found embedded in *Schlumbergera* by Calvente et al. (2011a) and Korotkova et al. (2011). Stem shape is, however, labile throughout the tribe, and Calvente et al. (2011a) found several transitions between terete and flattened stems.

Our phylogeny is at variance with the topologies of the just-cited studies, although the support for the early divergent nodes within the tribe is weak: We found *Rhipsalidopsis* and *Lepismium* as consecutive sisters to *Schlumbergera* + (*Hatiora* + *Rhipsalis*), while Calvente et al. (2011a) show *Rhipsalidopsis* as sister of *Schlumbergera*, and Korotkova et al. (2011) found *Schlumbergera* (incl. *H. epiphylloides*) and *Hatiora* as consecutive sisters to *Rhipsalidopsis* + (*Lepismium* + *Rhipsalis*). *Lepismium* in the revised circumscription discussed above and *Rhipsalis* are shown as monophyletic in all recent studies (Calvente et al. 2011a, 2011b; Korotkova et al. 2011). They are together characterized by pale translucent flowers (vs. brightly coloured in the other genera of the tribe).

**Notocacteae** Buxb. 1958

Incl. Parodieae Mottram 1990 (as `Parodiae’).

*Included genera*:

- *Eriosyce* (incl. *Diaguita* [not sampled], incl. *Guerreroa* [not sampled], incl. *Horridocactus*, incl. *Islaya*, incl. *Neoporteria*, incl. *Neotanahashia* [not sampled], incl. *Pyrrhocactus*, ?incl. *Rimacactus* [not sampled], incl. *Thelocephala* [not sampled])
- *Neowerdermannia*
- *Parodia* (incl. *Acanthocephala*, incl. *Brasiliparodia* [not sampled], incl. *Eriocephala*, incl. *Notocactus*, incl. *Wigginsia*)
- *Yavia*

Terrestrial globose dwarf to medium-sized plants distinctly confined to South America, spanning the highlands of Santa Catarina in S Brazil to S-C Argentina and to the high Andes and the arid coasts of C Chile to C Peru, with unsegmented stems and diurnal flowers of various constructions.

Another neatly circumscribed clade, consisting of four genera with small to medium-sized globose plants from South America. Our results show *Eriosyce* s.l. to consist of two clades, *Eriosyce* s.s. (incl. *Islaya*) and *Neoporteria* (incl. *Horridocactus* and *Pyrrhocactus*; *Thelocephala* not sampled), congruent with the topology of the general phylogenies of Bárcenas et al. (2011) and Hernández-Hernández et al. (2011) and the deeply sampled study of *Eriosyce* s.l. by Guerrero et al. (2019). *Eriosyce* likely has a convoluted biogeographic history with repeated West–East Andean disjunctions, witnessed by the Argentinian *E. umadeave* shown as sister to *Eriosyce* s.s. by Guerrero et al. (2019), and the remaining Argentinian taxa (i.e. the former segregate *Pyrrhocactus*, represented by *E. strausiana* in our study) are shown embedded amongst the remaining clades of *Eriosyce* s.l. Enigmatically, Guerrero et al. (2019) show *E. laui* (not sampled) from northern Chile as part of the clade formed by the other three genera of the tribe, as sister of *Yavia* and *Neowerdermannia*, lending support to its segregation as monotypic *Rimacactus*. *Yavia* (1 species, Argentina) and *Neowerdermannia* (2 species, Argentina, Bolivia, Peru, northern Chile) have distinct high-Andean distributions, while *Rimacactus* is a narrow endemic on the western Andean slopes in northern Chile.

*Parodia* s.l. (incl. *Notocactus*, *Wigginsia*, etc.) is confirmed to form a monophyletic entity, but the internal topology at the root involving the narrowly distributed eastern South American *Acanthocephala* (with nomenclatural priority over the widely used name *Brasilicactus*), restricted to southern Brazil (Rio Grande do Sul, Santa Catarina) and *Eriocephala* (with nomenclatural priority over the widely used name *Eriocactus*), restricted to southern Brazil, Paraguay, and northeastern Argentina, is ill-supported. The unsampled segregate *Brasiliparodia* has a similarly restricted occurrence in southern Brazil. All these taxa have very narrow distribution ranges and are confined to azonal rocky habitats (often sheer rock faces) in rather humid climates, and all are likely isolated relicts cladistically ancestral to the more species-rich subclades of *Notocactus, Wigginsia,* and *Parodia* s.s.

**Cereeae** Salm-Dyck 1840 (as ‘Cereastrae’)

Incl. Melocacteae Salm-Dyck 1840, incl. Harrisieae Buxb. (as *‘*Harrisiae’) 1958, incl. Trichocereeae Buxb. 1958 (as *‘*Trichocereae’), incl. Browningieae Buxb. 1966, incl. Echinopsideae H.Friedrich and G.D.Rowley 1976, incl. Acanthocalycieae Lakomski 2003 (nom. inval.).

Terrestrial or very rarely epiphytic (*Echinopsis arboricola*) plants, from diminutive globose solitary to clustering bodies to iconic solitary columns and candelabriform trees, often with distinctly modified reproductive stem parts (cephalia), stems segmented or not, with vastly variable flowers.

Tribe *Cereeae* is a well-supported monophyletic clade almost completely confined to South America, with only a handful of species of *Cereus*, *Harrisia*, *Melocactus* and *Pilosocereus* extending to Central America, Mexico and the Caribbean and the southeastern USA (Florida). Support for the early nodes within the tribe is incomplete, but recent studies taken together suggest that the evolutionary history is best represented by accepting a grade of monogeneric or genus-poor clades at the rank of subtribes. This contrasts the paraphyletic grade recognized as *Rebutiinae by Nyffeler and Eggli (2010a).

The majority of species diversity falls into the large subtribes Cereinae and Trichocereinae, which are both morphologically exceedingly diverse—both span the continuum from dwarf solitary globose taxa to iconic arborescent forms. Specially modified reproductive stem parts (cephalia) are notable in several clades of both these large subtribes: Terminal and ring-like cephalia only occur in Brazilian members of Cereinae, but lateral cephalia are present in several clades of both Cereinae and Trichocereinae. Lateral cephalia have repeatedly been used as morphological “key” character to broadly circumscribe *Micranthocereus* or *Espostoa*, but they have without doubts evolved repeatedly in parallel and make the broad concepts of these two genera untenable.

In our present data, the first-formed branch in tribe *Cereeae* conforms to a strongly supported monophyletic genus *Aylostera*, formally recognized as subtribe Aylosterinae by Romeiro-Brito et al. (2023b). The next-branching clade (subtribe Rebutiinae) is enigmatic and suffers from pronounced uncertainties regarding its topology—and probably associated with the extreme growth form diversity (columnar to branched shrubs in *Browningia*, small globose plants in *Rebutia* and *Weingartia*).

The placement of *Uebelmannia* (not sampled by us, comprising 4 microendemic species with patchy occurrence in campo rupestre vegetation in northeastern Brazil, cf. Silva et al. (2020) and Zappi et al. 2024) as a further monogeneric clade amongst the early-branching nodes of Cereeae is stimulated by the results of several papers: Bárcenas et al. (2011) place *Uebelmannia* in a vast polytomy embracing all sampled Cereeae. Fantinati et al. (2021) found the single analysed species of *Uebelmannia* embedded in Cereeae, as sister to the remaining genera of Cereinae, but support for most nodes in their phylogeny is moderate. In contrast, Ritz et al. (2007) and Hernández-Hernández et al. (2011) show *Uebelmannia* as sister to all other genera of the tribe, and this position is corroborated by Romeiro-Brito et al. (2022, 2 species studied) and Romeiro-Brito et al. (2023b, 3 species studied). At this time, the exact position of *Uebelmannia*, whose type species (*U. gummifera*) is characterized by a peculiar and unique stem anatomy with conspicuous gum ducts, remains incompletely resolved. Historically, the genus was placed in Notocacteae by Barthlott and Hunt (1993) and Buxbaum in Endler and Buxbaum (1974), and in Trichocereeae by Taylor and Zappi (2004).

The remaining species-rich subtribes Cereinae and Trichocereinae in their traditional circumscriptions share the pattern of a monogeneric earliest branch as sister to the rest of the diversity (*Gymnocalycium* in Cereinae, *Reicheocactus* in Trichocereinae), and we suggest to accept these two genera as additional monogeneric subtribes Gymnocalyciinae and Reicheocactinae (see below).

**Uebelmanniinae** N.P.Taylor 2023

*Included genus*:

- *Uebelmannia*

The type species *Uebelmannia gummifera* was originally described as *Parodia*, based on the superficially similar globose bodies and yellow flowers. The genus currently embraces 4 species and is confined to central Minas Gerais (Brazil) (Taylor and Zappi 2004; Zappi et al. 2024). Its circumscription was never challenged so far.

**Aylosterinae** N.P.Taylor 2023

*Included genus*:

- *Aylostera* (incl. *Digitorebutia*, incl. *Mediolobivia*)

The polyphyly of *Rebutia* s.l. in the sense of Anderson (2001) or Anderson (2005) (including *Aylostera*, *Digitorebutia* and *Mediolobivia*) or Hunt et al. (2006) (including in addition also *Cintia* and *Weingartia*) was already evident in the study of Ritz et al. (2007) and was corroborated by Mosti et al. (2011) and Ritz et al. (2016). Recognizing *Aylostera* and *Rebutia* as separate genera is congruent with differences in pollen morphology (6- to 8-colpate in *Aylostera*, 3-colpate in *Rebutia* s.s.) (Garralla et al. 2008).

*Aylostera* was studied in detail by Ritz et al. (2016) and was found to be divisible into three clades which conform to *Aylostera* s.s. and the former segregates *Digitorebutia* and *Mediolobivia* (of which we sampled *A. einsteinii*, regarded as synonym of the type species *A. aureiflora* by Ritz et al. 2016). They form a fully supported and resolved clade in our analysis. The relative order of the three terminals of *Aylostera* s.l. we analysed, *Mediolobivia* (*A. einsteinii*) + (*Aylostera* s.s. + *Digitorebutia*), is similar to that seen in Ritz et al. (2007), but differs from that of the more densely sampled phylogeny of Ritz et al. (2016), which found (*Mediolobivia* + *Aylostera*) + *Digitorebutia*.

**Rebutiinae **Donald 1955

Incl. Browningiinae Lakomski 2003 (nom.inval.).

*Included genera*:

- *Browningia* (excl. *Castellanosia* [see tribe Phyllocacteae subtribe Leptocereinae], but incl. *Azureocereus* [not sampled], incl. *Gymnanthocereus* [not sampled], incl. *Gymnocereus* [not sampled])
- *Rebutia* s.s.
- *Weingartia* (incl. *Cintia* [not sampled], incl. *Gymnorebutia*, incl. *Sulcorebutia*).

The clade formed by *Rebutia*, *Browningia* s.s., and *Weingartia* is one of the most enigmatic relationships found in the whole family, first evident in provisional data of Lendel et al. (2006) and Ritz et al. (2007), but also present in the phylogeny presented by Schlumpberger and Renner (2012)—the dissimilarities between the often very dwarf and partially geophytic globose bodies of *Rebutia* and *Weingartia* and the iconic branched trees of *Browningia* could not be more pronounced and the only readily identifiable character that unites the genera are the naked axils of the pericarpel scales. The low support for any particular topology (*Rebutia* + (*Browningia* + *Weingartia*) in our data, or a paraphyletic grade of *Browningia* including *Rebutia* s.s. as sister to *Weingartia* in Ritz et al. (2007)) is likely due to a long-isolated development of *Browningia* relative to *Rebutia* and *Weingartia*. Including additional species into analyses, ideally representing the proposed segregates *Gymnanthocereus*, *Azureocereus* and *Gymnocereus*, will hopefully allow to gain a better picture in the future. Further, it should be noted that Schlumpberger and Renner (2012) show *Lasiocereus fulvus* as sister to the *Browningia*–*Rebutia*–*Weingartia*-clade, while our study identified the type of the genus, *L. rupestris*, as belonging to *Trichocereinae*.

The monophyly of *Weingartia* s.l. is well supported in our data, in contrast to the more densely sampled studies of Ritz et al. (2007) and Mosti et al. (2011).

**Gymnocalyciinae** N.P.Taylor 2023

*Included genus*:

- *Gymnocalycium* (incl. *Brachycalycium* [not sampled])

*Gymnocalycium* is confined to the plains and Andean slopes of eastern South America from southern Brazil and Uruguay to Paraguay, Bolivia, and Argentina (in the South well into Patagonia). The broadly sampled recent studies concur that *Gymnocalycium* is monophyletic (Ritz et al. 2007; Meregalli et al. 2010; Demaio et al. 2011; Mosti et al. 2011) but arrive at different internal topologies for the genus. The placement of the genus within Cereeae remains controversial to some extent. Ritz et al. (2007) found *Gymnocalycium* as sister to Trichocereinae, though with low support, rather than as sister to Cereinae, as in our phylogeny and by Romeiro-Brito et al. (2022) and Romeiro-Brito et al. (2023b). The genus is, without doubt, very isolated within the Cereeae, and we therefore propose to accept the monogeneric subtribe Gymnocalyciinae for it.

**Cereinae** Britton & Rose 1920 (as ‘Cereanae’)

Incl. Discocactinae Buxb. 1958, incl. Melocactinae Doweld 2002, incl. Pilosocereinae Doweld 2002.

A clade almost completely confined to eastern and northeastern South America (but *Cereus* extending north-wards to the Caribbean, *Melocactus* and *Pilosocereus* extending north-wards to Central America, the Caribbean and Mexico, *Pilosocereus* also extending to the southeastern USA (Florida), and *Pilosocereus*, *Praecereus* and *Melocactus* extending southwest-wards to Peru). The vast majority of the diversity of this clade is made up by genera confined to northeastern Brazil. This subtribe is notable for the occurrence of cephalia in several genera—lateral in *Coleocephalocereus, Espostoopsis*, *Facheiroa* and *Micranthocereus* s.l., terminal in *Discocactus* and *Melocactus*, and ring-like in *Arrojadoa* and *Stephanocereus*.

Our sampling of the subtribe is somewhat sketchy, esp. in comparison with the recent deeply sampled multi-gene study of Romeiro-Brito et al. (2023b, using the Cactaceae591 probe set developed by this team). In general, our backbone does not differ substantially from that of Romeiro-Brito et al. (2023b), which is highly remarkable, since their probe set Cactaceae591 (459 loci) shares merely 3 loci with our Angiosperms353 probe set (318 loci)—a very strong indication that the uncertainties and insufficient support values of many nodes are not artefacts but the result of rapid diversification events. In the following discussion, we intersperse our results with the data of Romeiro-Brito et al. (2023b) to give a full picture of present knowledge. It should be noted that most of the clades derived from analyses of the Cactaceae591 data show limited support (LPP < 0.7), and decisions to accept/synonymize genera are difficult to justify on this weak base.

The Cereinae consist of a basal unresolved grade within which we recover a well-supported, unambiguously circumscribed and species-rich informal clade:

- Cladistically basal unresolved grade of early-diverging genera: *Included genera*:
  - *Brasilicereus* (?incl. *Bragaia* [not sampled])
  - *Cereus* (incl. *Mirabella* [not sampled], ?incl. *Serrulatocereus* [not sampled], incl. *Subpilocereus*)
  - *Cipocereus* (not sampled, excl. *Floribunda,* see Informal Clade below)
  - *Facheiroa* (incl. *Zehntnerella* [not sampled])
  - *Leocereus*
  - *Praecereus*
  - *Stetsonia*

This phylogenetically basal grade conforms to the *Cereus* clade, the *Facheiroa* clade plus *Stetsonia* and *Praecereus* of Romeiro-Brito et al. (2023b).

The position of the small genera *Praecereus* and *Stetsonia* as early nodes of the grade is congruent with their flower architecture—naked axils of the perianth scales is the predominant condition in the subtribe. The exact topology is still uncertain, however. Contrary to our data, Franco et al. (2017), Romeiro-Brito et al. (2022) and Romeiro-Brito et al. (2023b) found *Stetsonia* as sister to the remaining genera of the subtribe. *Praecereus* forms a clade with two species of *Cereus* in the phylogeny of Romeiro-Brito et al. (2016, their Data Supplement S1), while Franco et al. (2017) and Romeiro-Brito et al. (2022) found it as sister to all species of *Cereus* and *Cipocereus.* In contrast, Fantinati et al. (2021) show *Praecereus* deeply embedded in *Cereus*. The relationships amongst major clades in *Cereus* are still largely unresolved. The two species from the Caribbean studied so far form a monophyletic branch in the data of Romeiro-Brito et al. (2023b), but not in the study of Franco et al. (2017).

The placement of *Cipocereus* (not sampled by us) is also unresolved: Franco et al. (2017) found the genus as sister to the species of the former segregate *Mirabella*, as sister to *Praecereus* and the other clades of *Cereus*. In contrast, Amaral et al. (2021) and Romeiro-Brito et al. (2023b) show *Praecereus* and *Cipocereus* as consecutive sisters of *Cereus*, and Romeiro-Brito et al. (2022) have conflicting evidence (depending on different datasets used) for *Cipocereus* either embedded in *Cereus*, or as sister to *Mirabella.* This later topology was also found by Fantinati et al. (2021). Together, these disparate trees could argue for a wider circumscription of *Cereus*, to include both *Cipocereus* and *Praecereus*.

The close association of *Leocereus* and *Facheiroa* seen in our topology was first visible in the tree of Schlumpberger and Renner (2012), and is mirrored in their shared flower architecture, with pericarpels having numerous closely set scales with often abundant felt in their axils that is unique in the two genera in the subtribe. Historically, both genera were classified in Trichocereinae (as Trichocereeae) by Barthlott and Hunt (1993) and Taylor and Zappi (2004), based on sharing hair-bearing pericarpel areoles with that subtribe. Interestingly, Fantinati et al. (2021) found *Leocereus* as sister of *Brasilicereus* (*Facheiroa* s.s. not analysed, *F. squamosa* misplaced in their tree), while Romeiro-Brito et al. (2022) show *Facheiroa* as sister to *Brasilicereus* (*Leocereus* not analysed). In our data, *Brasilicereus* is not part of the *Facheiroa*-*Leocereus* clade but shown as sister to the remaining genera of Cereinae, though with low support. The denser sampling of Romeiro-Brito et al. (2023b) also included *Bragaia estevesii*, a monotypic genus also sometimes included in the very similar *Brasilicereus*, from which it can hardly be separated on morphological grounds. They resolved a fully supported clade (their *Facheiroa* clade) with *Brasilicereus* s.s. + (*Bragaia* + (*Facheiroa* + *Leocereus*)), and in order to resolve the paraphyletic grade *Brasilicereus* s.l. proposed to include *Bragaia, Brasilicereus* and *Leocereus* in an expanded *Facheiroa*. In view of the pronounced morphological differences and the inconsistent findings about the position of *Brasilicereus* between the Angiosperms353 and Cactaceae591 datasets, we argue that this is premature and favour accepting the three traditionally recognized genera in question for the time being. The close relationship of *Brasilicereus* with its pericarpels having rather few scales with completely naked axils in close association with taxa with numerous pericarpel scales and felted axils is another example of how labile flower characters in Cereeae can be.

The recently segregated *Serrulatocereus* is a nomenclatural conundrum, based on *Cereus serruliflorus* Haworth, which is typified by a Plumier illustration. Depending on the interpretation of this composite illustration, the name is either a synonym of *Harrisia divaricata* (e.g. Anderson 2005) or belongs (as *C. serruliflorus* or *C. ayisyen*, the latter replacing the illegitimate *C. haitiensis* of Franck et al. 2017) to the genus *Cereus* as subgen. *Neohaiticereus* (Areces-Mallea 2018) or subgen. *Arecesocereus* (Wisnev 2018).

- Informal Clade: = Melocactinae Doweld 2002 + Pilosocereinae Doweld 2002: *Included genera*:
  - *Arrojadoa* (incl. *Arrojadoopsis* [not sampled])
  - *Coleocephalocereus* (incl. *Buiningia*, incl. *Mariottia* [not sampled])
  - *Discocactus*
  - *Espostoopsis*
  - *Floribunda*
  - *Lagenosocereus* (not sampled)
  - *Melocactus*
  - *Micranthocereus* (incl. *Austrocephalocereus* [not sampled], incl. *Viridicereus* [not sampled])
  - *Pierrebraunia* (not sampled)
  - *Pilosocereus*
  - *Siccobaccatus* (not sampled)
  - *Stephanocereus*
  - *Xiquexique* (?incl. *Caerulocereus* [not sampled])

The novel placement of the monogeneric *Espostoopsis* from northeastern Brazil (formerly considered as belonging to Trichocereinae (e.g. Taylor and Zappi (2004), as Trichocereeae), due in part to the superficial similarity with *Espostoa*) amongst the earliest diverging branches of this informal clade evident in our data and also found by Romeiro-Brito et al. (2022) and Romeiro-Brito et al. (2023b) is congruent with the formation of a lateral cephalium that is also present in *Facheiroa* p.p., *Micranthocereus*, and *Coleocephalocereus*. Schlumpberger and Renner (2012) found *Espostoopsis* as sister to *Micranthocereus densiflorus*, but their sampling of the Brazilian taxa was sketchy. The exact topology of the early-diverging clades varies amongst the analyses—our data recovered *Micranthocereus* s.s. as sister to all remaining genera of the Informal Clade of Cereinae (= combined *Micranthocereus*, *Melocactus* and *Pilosocereus* clades of Romeiro-Brito et al. 2023b), while Romeiro-Brito et al. (2023b) in their re-analysis of part of the publicly available PAFTOL (Angiosperms353) data recovered *Espostoopsis* as sister of *Micranthocereus* s.s. + the rest. We abstain from naming the subclades containing *Espostoopsis* / *Micranthocereus* (s.s.) / *Melocactus* / *Pilosocereus* formally (in contrast to Romeiro-Brito et al. 2023b), as we argue that formal recognition (and naming) is only warranted when a clade is recovered with high support values and is subtended by a long branch; a criterion not met by the current results.

While the overall topology of the genera of this clade remains largely unresolved in our data (mirroring the results of Romeiro-Brito et al. (2022) in part), indicating a massive burst of diversification of the group in northeastern Brazil, several small well-supported clades emerge:

*Coleocephalocereus* s.l., i.e. including *Buiningia*, is completely supported by our data, and Romeiro-Brito et al. (2023b) confirm this. The same applies to the close relationship between *Discocactus* and *Melocactus*—they share usually unbranched bodies with terminal cephalia and differ merely in their floral syndromes, nocturnal and sphingophilous in *Discocactus*, and diurnal and ornithophilous in *Melocactus*.

The close association between *Stephanocereus* and *Arrojadoa* (also evident in the phylogenies presented by Fantinati et al. (2021) and Romeiro-Brito et al. (2023b)) is similarly mirrored in their cephalium architecture—ring-like cephalia alternating with sterile elongate stem segments are unique for this group (but the same architecture is also present in the Mexican *Cephalocereus apicicephalium* from tribe Phyllocacteae subtribe Echinocereinae), and the genera again differ in floral syndromes (nocturnal and chiropterophilous in *Stephanocereus*, diurnal or crepuscular and ornithophilous in *Arrojada*). The placement of *Floribunda pusilliflora* (commonly treated as species of *Cipocereus*, e.g. Taylor and Zappi 2004) as sister to the *Arrojadoa*–*Stephanocereus* clade was also found by Fantinati et al. (2021) and Romeiro-Brito et al. (2023b). The species-rich sampling of Romeiro-Brito et al. (2023b) identified *Pierrebraunia bahiensis* and *Stephanocereus luetzelburgii* (also segregated as monotypic genus *Lagenosocereus* = *Stephanocereus* subgen*. Lagenopsis*) as further members of the *Arrojadoa–Stephanocereus* clade, together with *Micranthocereus violaciflorus*. This assemblage of taxa is remarkable since *Floribunda*, *Pierrebraunia* and *Lagenosocereus* do not form cephalia, *Micranthocereus violaciflorus* produces a lateral cephalium, and *Stephanocereus* and *Arrojadoa* form intermittent ring cephalia. In a radical approach, Romeiro-Brito et al. (2023b) include all of them in a vastly expanded genus *Arrojoadoa*. Since all the clades (except the position of *M. violaciflorus*) in their analysis are fully supported we argue that recognizing the individual clades as monogeneric genera *Floribunda* + ((*Pierrebraunia* + (*Lagenosocereus* + *Stephanocereus*) + *Arrojadoa* represents the sequence of evolutionary divergences much better. The placements of *Lagenosocereus* and *Pierrebraunia* here were also found by Fantinati et al. (2021). *Micranthocereus violaciflorus* is best treated as unresolved orphan for the time being—the separation as monotypic genus *Viridicereus* (Guiggi 2024) is premature.

The genera *Pilosocereus* s.l. and *Micranthocereus* have recently received considerable attention by several studies concentrating on Brazilian members of Cereinae, while our own sampling of the group is inadequate. Calvente et al. (2017), Lavor et al. (2019), Lavor et al. (2020), and Fantinati et al. (2021) found the recently segregated *Xiquexique* (formerly treated as *Pilosocereus* subgen. *Gounellea*, Taylor and Zappi (2004)) as well-supported clade separate from *Pilosocereus* s.s., and this is supported by the more in-depth studies of Romeiro-Brito et al. (2022) and Romeiro-Brito et al. (2023a). *Pilosocereus* (excl. *Xiquexique*) moreover is only monophyletic when *P. bohlei* is also excluded–Fantinati et al. (2021) found this enigmatic species as sister to all genera of our Clade 1, mirroring the phylogenies found by Calvente et al. (2017) and Lavor et al. (2020), while the data of Romeiro-Brito et al. (2023a) and Romeiro-Brito et al. (2023b) shows *P. bohlei* as sister to the other taxa of *Xiquexique*, though with limited support, and only in some of their separate analyses. Fantinati et al. (2021), Romeiro-Brito et al. (2022) and Romeiro-Brito et al. (2023b) found *Micranthocereus* in its current circumscription (e.g. Taylor and Zappi 2004) as polyphyletic: The type species *M. polyanthus* and a couple of additional species (incl. *Austrocephalocereus*) form the sister clade to *Xiquexique*. Fantinati et al. (2021) found two further species of *Micranthocereus* unplaced in a polytomy with the genera of Clade 1, and *M. aurei-azureus* as sister to *Coleocephalocereus goebelianus*. Romeiro-Brito et al. (2023b) recovered an even more pronounced polyphyly of *Micranthocereus*: *Micranthocereus* s.s. is sister to *Xiquexique*, while the species formerly sometimes segregated as *Siccobaccatus* are shown as sister to *Coleocephalocereus*, and are consequently moved there. While *Siccobaccatus* has a similar gross morphology as *Coleocephalocereus goebelianus*, its stem anatomy is completely different insofar as the tissue dries without collapsing (pers. obs. UE), and we thus suggest to accept the segregate genus *Siccobaccatus* to draw attention to this enigmatic difference. *Micranthocereus auriazureus* and *M. albicephalus* are placed as unresolved sister to the *Arrojoadoa*–*Stephanocereus* clade by Romeiro-Brito et al. (2023b), but with weak support and different topologies in the coalescent-based inference vs. the maximum likelihood tree (their fig. S3), and were treated as incertae sedis in Cereinae by these authors.

We included only the type of *Micranthocereus* in our sampling, and this represents an early-branching segregate in our results, too, but is not associated with our representative of *Xiquexique*, as detailed above.

All the studies by Calvente et al. (2017), Lavor et al. (2019), Lavor et al. (2020), Franco-Estrada et al. (2022) and Romeiro-Brito et al. (2023a) concur that the narrowly endemic *Pilosocereus aureispinus* from central Bahia is sister to all remaining taxa of the genus, and that the extra-Brazilian *Pilosocereus* species form a single clade embedded in *Pilosocereus* s.s., with *P. chrysostele* from northeastern Brazil as sister. This is a clear indication that the Caribbean and North America were colonized once only in the pleistocene (Lavor et al. 2019; Romeiro-Brito et al. 2023a).

**Reicheocactinae** Eggli, Nyffeler & J.M.de Vos, **subtrib**. **nov**.—Type: *Reicheocactus* Backeb.

*Included genus*:

- *Reicheocactus*

*Diagnosis*: Body dwarf, globose to very shortly cylindrical, unsegmented, usually solitary, with tuberous main root, with numerous closely set low ribs dissolved into semiglobose tubercles; spines pectinate, weak and hardly pungent; flowers diurnal, pericarpel scales with abundant wool and hairs in their axils.

The isolated position of *Reicheocactus* from the eastern Andean slopes of central Argentina as sister to all genera of subtribe Trichocereinae was first noted by Schlumpberger and Renner (2012) with good support, and our study corroborates this result, again with good support. Erecting a monogeneric subtribe underlines its isolated position. *R. famatimensis* has suffered from long-standing misidentification with *Echinopsis densispina* (Wessner 1940a, 1940b) and *Eriosyce odieri* (Krainz 1957).

**Trichocereinae** Buxb. 1958

Incl. Borzicactinae Buxb. 1958, incl. Echinopsidinae H.Friedrich & G.D.Rowley 1976, incl. Eriocereinae Doweld 2001, incl. Acanthocalyciinae Lakomski 2003 (nom. inval.).

For subtribe Trichocereinae, we recognize a phylogenetically basal grade and three informal clades—even though they are not overwhelmingly supported each is nevertheless coherent in its composition.

Trichocereinae are a completely South American clade with the exception of *Harrisia* (extending to the Caribbean and southeastern USA [Florida]) and embrace the whole diversity from small globose to tree-like columnar plants—growth form architecture as well as flower morphology (mirroring different pollination syndromes, including transitions, cf. Schlumpberger et al. (2009)) are extremely labile in this subtribe (Schlumpberger and Renner 2012). Several genera of this subtribe have suffered badly in the past from profoundly different classification approaches, involving the recognition of numerous segregates vs. few widely circumscribed genera such as *Cleistocactus* s.l. or *Echinopsis* s.l. (Anderson 2001, 2005; Hunt et al. 2006). Either approach is unsatisfactory, but an acceptable solution for all clades of the subtribe has not yet emerged, despite the deeply sampled analysis of Schlumpberger and Renner (2012). The concept of *Echinopsis* s.l. as advocated by Anderson (2001) and Anderson (2005) (*Acanthocalycium* and *Denmoza* accepted as separate genera) and Hunt et al. (2006) (only *Denmoza* accepted) is almost congruent with the well-supported Clade 1 (see below) in our phylogeny. However, the concept of *Cleistocactus* s.l. as advocated by Anderson (2001), Anderson (2005) and Hunt et al. (2006) is completely untenable and includes elements scattered over our Clade 2 and Clade 3. Overall, the situation of numerous traditionally recognized genera being polyphyletic, coupled with traditionally used diagnostic characters that have evolved repeatedly in parallel, approaches the situation in the North American clade Cactinae.

Most of the topology of *Trichocereinae* is only weakly supported in our data, though a grade as sister to three geographically rather coherent clades (Clades 1—3 in the discussion below) can be distinguished. The weak support for most of the topology in our tree is mirroring the situation found by Schlumpberger and Renner (2012) in their deeply sampled study of the group. However, only part of their general relationships are shared with our phylogeny, and especially the divergent placements of *Harrisia*, *Weberbauerocereus* and the clades making up *Echinopsis* s.l. are notable: In Schlumpberger and Renner (2012), *Weberbauerocereus* is shown as sister to *Vatricania* + *Cleistocactus* s.s., and *Harrisia* + *Leucostele* (not sampled by us) is found as sister to *Echinopsis* s.s. Schlumpberger and Renner (2012) also sampled *Arthrocereus* (not sampled for our study) and found it as sister to the group just discussed. Franck et al. (2013) found no clear placement for this genus, which clustered with very different allies depending on the four different markers they studied. Romeiro-Brito et al. (2022) found *Arthrocereus* as sister to *Harrisia* + all remaining clades of the Trichocereinae, while Romeiro-Brito et al. (2023b) recovered a clade *Arthrocereus* + *Harrisia* as sister to the remaining Trichocereinae, but the topology in either case is again weakly supported and there is conflicting evidence in the results of Romeiro-Brito et al. (2022) between the two different sets of genes analysed. Clearly, more studies are necessary, but both *Harrisia* and *Arthrocereus* are amongst the early-diverging clades of the subtribe, and both are monophyletic in the data of Romeiro-Brito et al. (2023b). The placement of *Leucostele* is similarly unresolved: Schlumpberger and Renner (2012) show it as sister to *Harrisia* in their *Cleistocactus*-*Echinopsis* clade, Franck et al. (2013) show completely disparate placements (sometimes the three analysed species not even form a monophyletic group) depending on the marker analysed, and Romeiro-Brito et al. (2023b) recovered *Leucostele* in their *Echinopsis* clade, as sister to *Soehrensia formosa*. In view of these uncertainties, we tentatively place *Leucostele* amongst the early-diverging lineages in the subtribe.

*Echinopsis* s.l. (excluding *Leucostele* and *Reicheocactus*) is spread over two major clades (“*Echinopsis* clade”, and the clade making up the continuation of their Fig. 1) in the phylogeny of Schlumpberger and Renner (2012). This is in stark contrast to our results, which find all these lineages as part of a well-supported monophyletic Clade 1, which is also evident as *Echinopsis* clade in the backbone derived from Cactaceae591 data by Romeiro-Brito et al. (2023b).

Our recognition of several informal clades in subtribe Trichocereinae should not be overvalued, however. All genera of the subtribe are very closely related, as witnessed by the multitude of artificially produced intergeneric hybrids known today (Rowley (1994) and Table 1 in Eggli and Giorgetta (2013); and several additional combinations have since been published). Most if not all these artificial hybrids are fertile, even in crosses involving genera from different clades, and can be used for further breeding, resulting in complex hybrid parentages—the whole subtribe could conveniently be referred to as the “*Echinopsis* comparium”. In contrast to the ease with which intergeneric hybrids are obtained in cultivation, naturally occurring hybrids are usually rare and restricted to solitary or few individuals amongst thousands of plants of the parent species, and are suspected to be sterile (e.g. Lowry 2000; Eggli and Giorgetta 2013). The best documented exception and thus the presence of hybrid swarms are crosses between species of *Haageocereus* and *Espostoa* (= × *Haagespostoa*) from Clade 3 (Arakaki et al. 2020).

- Grade of early-diverging lineages: *Included genera*:
  - *Arthrocereus* (not sampled; incl. *Chapadocereus* [not sampled])
  - *Harrisia* (incl. *Brasiliharrisia* [not sampled], *Eriocereus* [not sampled], incl. *Estevesia* [not sampled])
  - ?*Leucostele* (not sampled)
  - *Weberbauerocereus*

Biogeographically speaking, this grade is enigmatic—*Harrisia* (incl. *Eriocereus*) has a vastly scattered occurrence in South America (Argentina, Bolivia, Paraguay, disjunctly in northeastern Brazil) and the Caribbean, to reach Florida in the North. *Weberbauerocereus*, on the other hand, is restricted to the western Andean slopes of Peru. For *Harrisia*, Romeiro-Brito et al. (2023b) found the NE Brazilian *H. adscendens* as sister to two taxa from Paraguay, Bolivia and Argentina (no Caribbean species included in the analysis). According to the phylogeny of Franck et al. (2013), the Caribbean species of *Harrisia* form a single clade, sister to the Brazilian *H. adscendens*. The recently described monotypic *Estevesia* is a narrow endemic from central Brazil (Goiás) that shows an overall similarity with *Harrisia* (rather than *Cereus* (as *Mirabella*) and *Pseudoacanthocereus*, as suggested in the protologue), and shares similar seeds, and we tentatively place it as synonym under *Harrisia*.

- Clade 1 (*Echinopsis* s.l., = “*Echinopsis* clade” of Romeiro-Brito et al. 2023b): *Included genera*:
  - *Acanthocalycium*
  - *Denmoza*
  - *Echinopsis* s.l. (incl. *Acantholobivia*, incl. *Chamaecereus*, incl. *Helianthocereus* [not sampled], incl. *Lobivia* [not sampled], incl. *Pseudolobivia* [not sampled], incl. *Soehrensia*, incl. *Trichocereus* s.s.)
  - *Setiechinopsis*

Most of the diversity of this clade is part of a broadly defined genus *Echinopsis.* Except for a very few species, most of them classified in the former segregates *Soehrensia* and *Trichocereus*, this is an entirely East Andean clade. Most of the former segregates were defined by simple combinations of growth form and flower characteristics (time of anthesis, flower architecture and colour, pollination syndrome). It would be defendable to also include *Acanthocalycium*, *Denmoza* and *Setiechinopsis* in an even more broadly defined *Echinopsis*. These three genera made up the ill-supported *Denmoza* clade in the phylogeny of Schlumpber-ger and Renner (2012), with *Setiechinopsis* as sister to *Denmoza* + *Acanthocalycium* (the latter including *Echinopsis leucantha*), which differs from our well-supported relationship of *Acanthocalycium* + *Setiechinopsis* as sister to *Denmoza* plus the remaining *Echinopsis* clades. Our phylogeny is misleadingly simplified as a result of our sampling strategy, and the complex relationship shown between different species groups of *Echinopsis* s.l. by Schlumpberger and Renner (2012) is a clear hint that we do not yet have an understanding of how this Clade 1 diversified.

- Clade 2 (East Andean Clade = “*Cleistocactus* clade” of Romeiro-Brito et al. 2023b): *Included genera*:
  - *Cephalocleistocactus*
  - *Cleistocactus* s.s. (?incl. *Bolivicereus* [not sampled], incl. *Seticleistocactus* [not sampled])
  - *Cremnocereus* (not sampled)
  - *Samaipaticereus*
  - *Vatricana*
  - *Winterocereus* (= *Hildewintera*)
  - *Yungasocereus*

In contrast to the other clades of *Trichocereinae*, this clade is uniform as far as overall growth form and geography is concerned—all its members are columnar and ribbed, and all have an East Andean distribution, centred in the lowlands at the foot of the Andes in Bolivia and *Cleistocactus* extending to northern Argentina. Enigmatically, most of the lineages in Clade 2 are species-poor or monotypic. Most notably, even when *Borzicactus* (see Clade 3) is excluded from *Cleistocactus*, the genus is still shown as polyphyletic: The type species *C. baumannii* is shown as sister to all remaining members of Clade 2, while *Cleistocactus tarijensis*, assumed to be closely related to *C. baumannii*, is shown in a separate clade, as sister of *Cephalocleistocactus*. Romeiro-Brito et al. (2023b) suggest to rationalize the taxonomy of the clade and recognize an expanded *Cleistocactus* s.l. that includes *Samaipaticereus*, *Vatricania* and *Yungasocereus* as synonyms (*Cremnocereus* not covered). We argue that this is premature in view of the uncertainties in the topology of Clade 2, and prefer to maintain the segregates until a deeper sampling of the clades allows a more substantial evaluation.

*Vatricania* was for a long time regarded as synonym of *Espostoa* (e.g. Anderson 2001, 2005; Hunt et al. 2006); it was first found to belong into the immediate relationship of *Cleistocactus* s.s. by Schlumpberger and Renner (2012).

The recently described monotypic *Cremnocereus* from Bolivia, known from two occurrences in a very small area only (Slaba 2022), appears completely distinct but shares some characters with *Cleistocactus* s.s. but also with *Oreocereus*, differing in its flowers with a chiropterophilous syndrome (Lowry and Winberg 2017). Its placement here is completely tentative.

- Clade 3 (West Andean and High Andean Clade, including the Río Marañón valley, Peru, = “*Oreocereus* clade” of Romeiro-Brito et al. 2023b): *Included genera*:
  - *Borzicactus* (incl. *Borzicactella* [not sampled])
  - *Espostoa* (incl. *Pseudoespostoa* [not sampled], incl. *Thrixanthocereus*)
  - *Haageocereus* (incl. *Loxanthocereus* [not sampled], incl. *Peruvocereus* [not sampled])
  - *Lasiocereus*
  - *Matucana* (?incl. *Anhaloniopsis*, incl. *Eomatucana* [not sampled], ?incl. *Perucactus* [not sampled], incl. *Submatucana* [not sampled])
  - *Mila* (not sampled)
  - *Oreocereus* (incl. *Arequipa* [not sampled], incl. *Morawetzia* [not sampled])
  - *Oroya*
  - *Pygmaeocereus*
  - ?*Rauhocereus* (not sampled)

All nodes in the topology of Clade 3 are poorly supported, with the notable exception of the clade formed by *Thrixanthocereus* as sister to *Lasiocereus* + *Espostoa*, which is fully supported. The genus *Lasiocereus* (2 species) is narrowly endemic in northern Peru in the Río Marañón drainage. Ritter (1981) placed *Lasiocereus* in *Trichocereinae*, while Nyffeler and Eggli (2010a) placed it in the paraphyletic grade of early-diverging lineages in their *Rebutiinae, and indeed, Schlumpberger and Renner (2012) found *L. fulvus* as sister to (*Browningia* + *Sulcorebutia*) + (*Rebutia* + *Aylostera*), though with poor support. The novel well-supported placement of *L. rupicola* (the type of the genus) as sister of *Espostoa* s.s. is consistent with its occurrence in northern Peru and its shared general growth form and flower architecture (including hairs in the axils of the perianth scales), but it does not have the cephalium typical for *Espostoa*. Interestingly, a close relationship of *Lasiocereus* with *Thrixanthocereus* (a synonym of *Espostoa*) was already postulated by Ritter (1981) but he also noted similarities in flower traits with *Browningia* (as *Gymnanthocereus*). Some support for both these relationships is presented by Wittner (2018) who found very different seeds for the two species, and those of *L. fulvus* are indeed very similar to those of *Browningia* (*Gymnanthocereus*) *chlorocarpa.* Taken together this is evidence that the two species are not closely related and thus not congeneric.

The monotypic *Rauhocereus* (not sampled by us) is included here on the basis of Schlumpberger and Renner (2012) who found it as sister of *Espostoa lanata*.

Our data do not allow further conclusions concerning the relationships and circumscriptions of the remaining genera of Clade 3. The study of Schlumpberger and Renner (2012) is slightly more deeply sampled and shows *Matucana* and *Borzicactus* both as polyphyletic. A possible polyphyly of *Matucana* is also evident in our data, since *M. madisoniorum* (also separated as monotypic *Anhaloniopsis*) is resolved close to *Oroya*.

## Conclusion

The current study based on newly generated phylogenomic data using the Angiosperms353 set of nuclear loci recovered phylogenetic relations that are mostly congruent with other phylogenomic studies of the highly diverse Cactaceae. Because we could include almost 90% of all genera of the last generic checklist (Korotkova et al. 2021), plus many segregates of polyphyletic genus complexes, we were able to fully reevaluate suprageneric Cactaceae classification, though we note that fully resolving generic circumscription requires denser taxon sampling. Importantly, despite the very large amount of sequencing data, some parts of the tree remain poorly resolved, where a general pattern of species-poor grades followed by large clades is recovered (Fig. 2). This suggests that the low overall sequence divergence in Cactaceae is due to a series of nested, rapid radiations, and thus reflects their diversification history, rather than a methodological bias. In our classification, this process is reflected by formally naming two paraphyletic grades alongside 27 monophyletic higher taxa. The classification includes a detailed taxon-by-taxon justification and a complete generic synonymy, jointly representing a major leap forward in the understanding of the evolution and systematics of this remarkable plant family.

## Information on Electronic Supplementary Material

**Online Resource 1.** Accession and data availability information (including ENA run accession number, identification, taxonomic notes, and voucher information)..

**Online Resource 2.** Locus assembly success under the HybPiper pipeline for each sample (including inclusion decision), summary statistics on the locus properties in phylogenetic datasets QC, QC-P and its subsets QC-P-BS40 and QC-P-BS50, and information on loci and sequences excluded during paralog filtering.

**Online Resource 3.** Locus and accession information for the BUCKy dataset.

**Online Resource 4.** Alignments and RAxML gene trees for all included loci and all phylogenetic datasets.

**Online Resource 5.** ASTRAL tree based on the QC data set.

**Online Resource 6.** ASTRAL tree based on the QC-P data set.

**Online Resource 7.** ASTRAL tree based on the QC-P-BS40 data set.

**Online Resource 8.** ASTRAL tree based on the QC-P-BS50 data set.

## Supplementary Information

Below is the link to the electronic supplementary material.

Online Resource 1: Containing accession and data availability information (including ENA run accession number, identification, taxonomic notes, and voucher information). (PDF 62 KB)

Online Resource 2: Containing locus assembly success under the HybPiper pipeline for each sample (including inclusion decision), summary statistics on the locus properties in phylogenetic datasets QC, QC-P and its subsets QC-P-BS40 and QC-P-BS50, and information on loci and sequences excluded during paralog filtering (XLSX 510 KB)

Online Resource 3: Containing locus and accession information for the BUCKy dataset (PDF 46 KB)

Online Resource 4: Containing alignments and RAxML gene trees for all included loci and all phylogenetic datasets (ZIP 10372 KB)

Online Resource 5: Containing the ASTRAL tree based on the QC data set (PDF 495 KB)

Online Resource 6: Containing the ASTRAL tree based on the QC-P data set (PDF 485 KB)

Online Resource 7: Containing the ASTRAL tree based on the QC-P-BS40 data set (PDF 498 KB)

Online Resource 8: Containing the ASTRAL tree based on the QC-P-BS50 data set (PDF 489 KB)

## Data Availability

Raw genetic data (reads) are publicly available under persistent identifiers as stated in Online Resource 1. Analysed genetic data (alignments per locus) and phylogenetic data (gene and species trees) are available as Online Resource 4.
