# Supplementary material for: Phylogenomics and classification of Cactaceae based on hundreds of nuclear genes
Source: Plant Syst Evol. 2025 Aug 11;311(5):28. doi: 10.1007/s00606-025-01948-z (PMC12339657; doi:10.1007/s00606-025-01948-z)
Supplement: Supplementary file 1 — Online Resource 1: Containing accession and data availability information (including ENA run accession number, identification, taxonomic notes, and voucher information). (PDF 62 KB) [file 606_2025_1948_MOESM1_ESM.pdf]

Electronic Supplementary Material belonging to: Phylogenomics and classification of Cactaceae based on hundreds of nuclear genes; Plant Systematics and Evolution;
Jurriaan M. de Vos, Urs Eggli, Reto Nyffeler, Isabel Larridon, Catherine McGinnie, Niroshini Epitawalage, Olivier Maurin, Felix Forest and William J. Baker;
Corresponding author Jurriaan M. de Vos, University of Basel, email jurriaan.devos@unibas.ch.

Sample ID	Scientific name (prefered)	Scientific name (per aggregate)	Generic segregate	ENA scientific_name (filed-as)	ENA run acc.	Voucher	Name in tree
P04598	Acanthocalycium spiniflora	Echinopsis spiniflora	Acanthocalycium	Echinopsis spiniflora	ERR7618203	ZSS 10−0439−0	Acanthocalycium (Echinopsis) spiniflora P04598
P04547	Acanthocereus tetragonus	Acanthocereus tetragonus		Acanthocereus tetragonus	ERR12712746	ZSS 89−2219−b	Acanthocereus tetragonus P04547
P04572	Acharagma roseanum	Acharagma roseanum		Acharagma roseanum	ERR7618190	K 1998−3217	Acharagma roseanum P04572
P04661	Airampoa soehrensii	Airampoa soehrensii		Tunilla orurensis	ERR7618242	K 1979−2803	Tunilla soehrensii P04661
P09319	Anacampseros kurtzii	Anacampseros kurtzii	Grahamia−pp	Anacampseros kurtzii	ERR7619857	ZSS 10−0046−0	Anacampseros−Grahamia−pp kurtzii P09319
P09321	Anacampseros vulcanensis	Anacampseros vulcanensis	Grahamia−pp	Anacampseros vulcanensis	ERR7619858	ZSS 90−4035−0	Anacampseros−Grahamia−pp vulcanensis P09321
P09209	Aporocactus flagelliformis	Disocactus flagelliformis	Aporocactus	Disocactus flagelliformis	ERR7619811	ZSS 91−2542−0	Aporocactus (Disocactus) flagelliformis P09209
P04573	Ariocarpus retusus	Ariocarpus retusus		Ariocarpus retusus	ERR5033443	ZSS 87−1838−0	Ariocarpus retusus P04573
P04574	Armatocereus laetus	Armatocereus laetus		Armatocereus laetus	ERR7618191	ZSS 77−1937−0	Armatocereus laetus P04574
P04577	Arrojadoa rhodantha	Arrojadoa rhodantha		Arrojadoa rhodantha	ERR7618192	ZSS 11−0486−0	Arrojadoa rhodantha P04577
P04578	Astrophytum myriostigma	Astrophytum myriostigma		Astrophytum myriostigma	ERR7618193	ZSS 89−2897−d	Astrophytum myriostigma P04578
P09175	Austrocactus spiniflorus	Austrocactus spiniflorus		Austrocactus philippii	ERR7619795	ZSS 93−1061−b	Austrocactus spiniflorus P09175
P08084	Austrocylindropuntia exaltata	Austrocylindropuntia exaltata	ss	Austrocylindropuntia subulata	ERR7619441	ZSS 99−7308−0	Austrocylindropuntia−ss exaltata P08084
P09285	Austrocylindropuntia lagopus	Austrocylindropuntia lagopus	Punotia	Punotia lagopus	ERR7619844	ZSS HuSo−049−2	Austrocylindropuntia−Punotia lagopus P09285
P09179	Aylostera einsteinii	Rebutia einsteinii	Aylostera	Aylostera einsteinii	ERR7619796	ZSS 16−0289−0	Aylostera (Rebutia) einsteinii P09179
P09183	Aylostera fiebrigii	Rebutia fiebrigii	Aylostera	Rebutia fiebrigii	ERR7619798	ZSS 92−2623−0	Aylostera (Rebutia) fiebrigii P09183
P04582	Aztekium ritteri	Aztekium ritteri		Aztekium ritteri	ERR7618194	ZSS 86−2607−c	Aztekium ritteri P04582
P09251	Backebergia militaris	Pachycereus militaris	Backebergia	Mitrocereus militaris	ERR7619830	ZSS 84−3732−a	Backebergia (Pachycereus) militaris P09251
P04583	Bergerocactus emoryi	Bergerocactus emoryi		Bergerocactus emoryi	ERR7618195	ZSS 91−3016−0	Bergerocactus emoryi P04583
P08439	Blossfeldia liliputana	Blossfeldia liliputana		Blossfeldia liliputana	ERR7619498	BR 19921010−23	Blossfeldia liliputana P08439
P08417	Borzicactus ventimigliae	Borzicactus ventimigliae		Borzicactus sepium	ERR7619442	ZSS 89−1518−0	Borzicactus ventimigliae P08417
P04584	Brasilicereus phaeacanthus	Brasilicereus phaeacanthus		Brasilicereus phaeacanthus	ERR5034691	ZSS 91−1616−b	Brasilicereus phaeacanthus P04584
P04585	Brasiliopuntia brasiliensis	Brasiliopuntia brasiliensis		Brasiliopuntia brasiliensis	ERR7618196	ZSS 88−2991−0	Brasiliopuntia brasiliensis P04585
P08419	Browningia candelaris	Browningia candelaris		Browningia candelaris	ERR7619443	ZSS 99−8520−b	Browningia candelaris P08419
P09185	Calymmanthium substerile	Calymmanthium substerile		Calymmanthium substerile	ERR7619799	ZSS bngi−1540−0−8−2014	Calymmanthium substerile P09185
SRR5036296	Carnegiea gigantea	Carnegiea gigantea		Carnegiea gigantea	SRR5036296	SAMN04868325	Carnegiea gigantea SRR5036296 ncbi
P04587	Castellanosia caineana	Castellanosia caineana		Castellanosia caineana	ERR7618197	ZSS 10−1364−0	Castellanosia caineana P04587
P04553	Cephalocereus polylophus	Cephalocereus polylophus	Neobuxbaumia	Cephalocereus polylophus	ERR7618177	ZSS 94−2643−a	Cephalocereus−Neobuxbaumia polylophus P04553
P09187	Cephalocereus senilis	Cephalocereus senilis		Cephalocereus senilis	ERR7619800	ZSS 15−0095−0	Cephalocereus senilis P09187
P09191	Cereus fricii	Cereus fricii	Subpilocereus	Cereus fricii	ERR7619802	ZSS 93−1839−0	Cereus−Subpilocereus fricii P09191
P05024	Cereus hexagonus	Cereus hexagonus	ss	Cereus hexagonus	ERR7618269	K 2009−1967	Cereus−ss hexagonus P05024
P09189	Cereus jamacaru	Cereus jamacaru	ss	Cereus jamacaru	ERR7619801	ZSS 86−5065−0	Cereus−ss jamacaru P09189
P09197	Cleistocactus baumannii	Cleistocactus baumannii	ss	Cleistocactus baumannii	ERR7619805	ZSS 99−9185−a	Cleistocactus−ss baumannii P09197
P04590	Cleistocactus chrysocephalus	Cleistocactus chrysocephalus	Cephalocleistocactus	Cleistocactus chrysocephalus	ERR7618198	ZSS 85−1468−1b	Cleistocactus−Cephalocleistocactus chrysocephalus P04590
P04591	Cleistocactus tarijensis	Cleistocactus tarijensis	ss	Cleistocactus hyalacanthus	ERR7618199	K 1999−3627	Cleistocactus−ss tarijensis P04591

P04560	Coleocephalocereus aureus	Coleocephalocereus aureus	Buiningia	Coleocephalocereus aureus	ERR7618181	ZSS 82−2541−b	Coleocephalocereus−Buiningia aureus P04560
P09203	Coleocephalocereus fluminensis	Coleocephalocereus fluminensis	ss	Coleocephalocereus fluminensis	ERR7619808	ZSS 88−1544−b	Coleocephalocereus−ss fluminensis P09203
P08881	Copiapoa gigantea	Copiapoa gigantea		Copiapoa gigantea	ERR7619579	GENT Larridon−7	Copiapoa gigantea P08881
P05026	Corryocactus brevistylus	Corryocactus brevistylus	ss	Corryocactus brevistylus	ERR7618271	K 2001−3819	Corryocactus−ss brevistylus P05026
P09205	Corryocactus melanotrichus	Corryocactus melanotrichus	ss	Corryocactus melanotrichus	ERR7619809	ZSS 94−2568−e	Corryocactus−ss melanotrichus P09205
P05025	Corryocactus squarrosus	Corryocactus squarrosus	Erdisia	Corryocactus squarrosus	ERR7618270	K 2007−2086	Corryocactus−Erdisia squarrosus P05025
P08421	Coryphantha macromeris	Coryphantha macromeris	Lepidocoryphantha	Coryphantha macromeris	ERR7619444	ZSS 81−1507−b	Coryphantha−Lepidocoryphantha macromeris P08421
P08422	Coryphantha sulcata	Coryphantha sulcata	ss	Coryphantha sulcata	ERR7619445	ZSS 79−2490/0	Coryphantha−ss sulcata P08422
P04551	Cumulopuntia boliviana	Cumulopuntia boliviana	ss	Cumulopuntia boliviana	ERR7618176	ZSS 99−5113−0	Cumulopuntia−ss boliviana P04551
P09207	Cumulopuntia sphaerica	Cumulopuntia sphaerica	Sphaeropuntia	Cumulopuntia sphaerica	ERR7619810	ZSS 94−1455−0	Cumulopuntia−Sphaeropuntia sphaerica P09207
P04561	Cylindropuntia imbricata	Cylindropuntia imbricata		Cylindropuntia imbricata	ERR7618182	ZSS 99−6271−0	Cylindropuntia imbricata P04561
P04594	Denmoza rhodacantha	Denmoza rhodacantha		Denmoza rhodacantha	ERR7618201	ZSS 10−1709−0	Denmoza rhodacantha P04594
P09287	Discocactus zehntneri	Discocactus zehntneri		Discocactus zehntneri	ERR7619845	ZSS 10−1400−0	Discocactus zehntneri P09287
P08423	Disocactus biformis	Disocactus biformis	ss	Disocactus biformis	ERR7619446	ZSS 99−6114−0	Disocactus−ss biformis P08423
P04596	Echinocactus platyacanthus	Echinocactus platyacanthus	ss	Echinocactus platyacanthus	ERR5033444	K 1990−3076	Echinocactus−ss platyacanthus P04596
P09249	Echinocereus pensilis	Echinocereus pensilis	Morangaya	Eriosyce senilis	ERR7619829	ZSS 78−2635−0	Echinocereus−Morangaya pensilis P09249
P04597	Echinocereus viridiflorus	Echinocereus viridiflorus	ss	Echinocereus viridiflorus	ERR7618202	ZSS 10−0022−0	Echinocereus−ss viridiflorus P04597
P09219	Echinopsis bruchii	Echinopsis bruchii	Soehrensia	Echinopsis bruchii	ERR7619814	ZSS 77−1213−a	Echinopsis−Soehrensia bruchii P09219
P08062	Echinopsis chamaecereus	Echinopsis chamaecereus	Chamaecereus	Echinopsis chamaecereus	ERR7619447	ZSS 85−1839−2	Echinopsis−Chamaecereus chamaecereus P08062
P08063	Echinopsis eyriesii	Echinopsis eyriesii	ss	Echinopsis oxygona	ERR7619448	ZSS 98−1499−a1	Echinopsis−ss eyriesii P08063
P04600	Echinopsis macrogona	Echinopsis macrogona	Trichocereus	Trichocereus macrogonus	ERR7618205	ZSS 14−0002−0	Echinopsis−Trichocereus macrogona P04600
P09215	Echinopsis tegeleriana	Echinopsis tegeleriana	Acantholobivia	Echinopsis tegeleriana	ERR7619812	ZSS 93−2403−0	Echinopsis−Acantholobivia tegeleriana P09215
P04601	Epiphyllum phyllanthus	Epiphyllum phyllanthus		Epiphyllum phyllanthus	ERR7618206	ZSS 88−3000−0	Epiphyllum phyllanthus P04601
P05169	Eriosyce aurata	Eriosyce aurata	ss	Eriosyce aurata	ERR7618324	GENT Larridon−355	Eriosyce−ss aurata P05169
P09221	Eriosyce islayensis	Eriosyce islayensis	Islaya	Eriosyce islayensis	ERR7619815	ZSS 10−0013−0	Eriosyce−Islaya islayensis P09221
P04604	Eriosyce strausiana	Eriosyce strausiana	Pyrrhocactus	Eriosyce strausiana	ERR7618208	ZSS 96−1135−0	Eriosyce−Pyrrhocactus strausiana P04604
P04603	Eriosyce subgibbosa	Eriosyce subgibbosa	Neoporteria	Eriosyce subgibbosa	ERR7618207	ZSS 91−1894−0	Eriosyce−Neoporteria subgibbosa P04603
P04605	Escontria chiotilla	Escontria chiotilla		Escontria chiotilla	ERR7618209	ZSS 93−2194−a	Escontria chiotilla P04605
P09225	Espostoa blossfeldiorum	Espostoa blossfeldiorum	Thrixanthocereus	Espostoa blossfeldiorum	ERR7619817	ZSS 99−5825−a	Espostoa−Thrixanthocereus blossfeldiorum P09225
P09223	Espostoa lanata	Espostoa lanata	ss	Espostoa lanata	ERR7619816	ZSS 11−0609−a	Espostoa−ss lanata P09223
P09227	Espostoopsis dybowskii	Espostoopsis dybowskii		Espostoopsis dybowskii	ERR7619818	ZSS 90−4640−0	Espostoopsis dybowskii P09227
P08436	Eulychnia acida	Eulychnia acida		Eulychnia acida	ERR7619459	GENT 19780081	Eulychnia acida P08436
P04562	Facheiroa ulei	Facheiroa ulei		Facheiroa ulei	ERR7618183	ZSS 10−1088−0	Facheiroa ulei P04562
P09231	Ferocactus haematacanthus	Ferocactus haematacanthus		Ferocactus haematacanthus	ERR7619820	ZSS 16−0309−0	Ferocactus haematacanthus P09231
P09229	Ferocactus wislizeni	Ferocactus wislizeni		Ferocactus wislizeni	ERR7619819	ZSS 10−2474−0	Ferocactus wislizeni P09229
P09195	Floribunda pusilliflorus	Cipocereus pusilliflorus	Floribunda	Cipocereus pusilliflorus	ERR7619804	ZSS 80−3255−0	Floribunda (Cipocereus) pusilliflorus P09195
P09233	Frailea chiquitana	Frailea chiquitana		Frailea chiquitana	ERR7619821	ZSS 99−9359−0	Frailea chiquitana P09233
P04608	Geohintonia mexicana	Geohintonia mexicana		Geohintonia mexicana	ERR7618210	ZSS 99−1237−0	Geohintonia mexicana P04608
P04609	Glandulicactus uncinatus	Sclerocactus uncinatus	Glandulicactus	Ferocactus uncinatus	ERR7618211	ZSS 16−0434−0	Glandulicactus (Sclerocactus) uncinatus P04609
P09235	Grusonia bradtiana	Grusonia bradtiana	ss	Grusonia bradtiana	ERR7619822	ZSS 17−0162−0	Grusonia−ss bradtiana P09235
P04610	Grusonia clavata	Grusonia clavata	Corynopuntia	Grusonia clavata	ERR7618212	ZSS 99−4235−0	Grusonia−Corynopuntia clavata P04610
P04611	Grusonia marenae	Grusonia marenae	Marenopuntia	Grusonia marenae	ERR7618213	ZSS 99−8432−a	Grusonia−Marenopuntia marenae P04611

P04613	Gymnocalycium gibbosum	Gymnocalycium gibbosum		Gymnocalycium gibbosum	ERR5033445	K 1980−3955	Gymnocalycium gibbosum P04613
P08060	Haageocereus pseudomelanostele	Haageocereus pseudomelanostele		Haageocereus pseudomelanostele	ERR7619449	ZSS 88−1991−0	Haageocereus pseudomelanostele P08060
P05035	Harrisia tortuosa	Harrisia tortuosa	Eriocereus	Harrisia tortuosa	ERR7618272	K 1985−2774	Harrisia−Eriocereus tortuosa P05035
P09239	Hatiora salicornioides	Hatiora salicornioides	ss	Hatiora salicornioides	ERR7619824	ZSS 95−2343−1	Hatiora−ss salicornioides P09239
P09289	Hylocereus guatemalensis	Hylocereus guatemalensis		Selenicereus guatemalensis	ERR7619846	ZSS 92−3014−0	Hylocereus guatemalensis P09289
SRR8327214	Hylocereus undatus	Hylocereus undatus		Hylocereus undatus	SRR8327214	SAMN10583669	Hylocereus undatus SRR8327214 ncbi
P09263	Isolatocereus dumortieri	Stenocereus dumortieri	Isolatocereus	Stenocereus dumortieri	ERR7619836	ZSS 95−1403−1	Isolatocereus (Stenocereus) dumortieri P09263
P09241	Lasiocereus rupicola	Lasiocereus rupicola		Lasiocereus rupicola	ERR7619825	ZSS 10−1469−0	Lasiocereus rupicola P09241
P09303	Lemaireocereus hollianus	Pachycereus hollianus	Lemaireocereus	Lemaireocereus hollianus	ERR7619853	ZSS 90−2327−0	Lemaireocereus (Pachycereus) hollianus P09303
P04617	Leocereus bahiensis	Leocereus bahiensis		Leocereus bahiensis	ERR7618214	ZSS 88−3184−d	Leocereus bahiensis P04617
P04563	Lepismium cruciforme	Lepismium cruciforme		Lepismium cruciforme	ERR7618184	ZSS 78−1419−0	Lepismium cruciforme P04563
P09257	Lepismium miyagawae	Pfeiffera miyagawae	Lepismium	Pfeiffera miyagawae	ERR7619833	ZSS 85−2177−0	Lepismium (Pfeiffera) miyagawae P09257
P04593	Leptocereus nudiflorus	Leptocereus nudiflorus	Dendrocereus	Dendrocereus nudiflorus	ERR7618200	ZSS 10−1937−c	Leptocereus−Dendrocereus nudiflorus P04593
P09243	Leptocereus quadricostatus	Leptocereus quadricostatus		Leptocereus quadricostatus	ERR7619826	ZSS 93−1853−0	Leptocereus quadricostatus P09243
P04618	Leuchtenbergia principis	Leuchtenbergia principis		Leuchtenbergia principis	ERR7618215	ZSS 97−1443−0	Leuchtenbergia principis P04618
P04619	Leuenbergeria quisqueyana	Pereskia quisqueyana	Leuenbergeria	Leuenbergeria quisqueyana	ERR7618216	ZSS 15−0167−0	Leuenbergeria (Pereskia) quisqueyana P04619
SRR5137211	Lophocereus schottii	Pachycereus schottii	Lophocereus	Pachycereus schottii	SRR5137211	SAMN06196470	Lophocereus (Pachycereus) schottii SRR5137211 ncbi
P04620	Lophophora williamsii	Lophophora williamsii		Lophophora williamsii	ERR7618217	K 1985−345	Lophophora williamsii P04620
P09247	Maihuenia poeppigii	Maihuenia poeppigii		Maihuenia poeppigii	ERR7619828	ZSS 99−3081−0	Maihuenia poeppigii P09247
SRR7905849	Maihuenia poeppigii	Maihuenia poeppigii		Maihuenia poeppigii	SRR7905849	SAMN10132135	Maihuenia poeppigii SRR7905849 ncbi
P04564	Maihueniopsis glomerata	Maihueniopsis glomerata		Maihueniopsis glomerata	ERR7618185	ZSS 10−0434−0	Maihueniopsis glomerata P04564
P04565	Mammillaria mammillaris	Mammillaria mammillaris	ss	Mammillaria mammillaris	ERR7618186	ZSS 82−2337−b	Mammillaria−ss mammillaris P04565
P04623	Mammilloydia candida	Mammillaria candida	Mammilloydia	Mammillaria candida	ERR7618218	ZSS 16−0339−0	Mammilloydia (Mammillaria) candida P04623
P09245	Marginatocereus marginatus	Pachycereus marginatus	Marginatocereus	Lophocereus marginatus	ERR7619827	ZSS 85−3304−0	Marginatocereus (Pachycereus) marginatus P09245
P09299	Marshallocereus aragonii	Stenocereus aragonii	Marshallocereus	Marshallocereus aragonii	ERR7619851	ZSS 90−4235−2	Marshallocereus (Stenocereus) aragonii P09299
P04552	Matucana haynei	Matucana haynei	ss	Matucana haynei	ERR5033441	ZSS 79−4129−0	Matucana−ss haynei P04552
P04624	Matucana madisoniorum	Matucana madisoniorum	Anhaloniopsis	Matucana madisoniorum	ERR7618219	ZSS 94−1437−a	Matucana−Anhaloniopsis madisoniorum P04624
P09291	Melocactus oreas	Melocactus oreas		Melocactus oreas	ERR7619847	ZSS 10−1071−e	Melocactus oreas P09291
P04625	Micranthocereus polyanthus	Micranthocereus polyanthus	ss	Micranthocereus polyanthus	ERR7618220	ZSS 10−1079−c	Micranthocereus−ss polyanthus P04625
P09237	Micropuntia pulchella	Grusonia pulchella	Micropuntia	Grusonia pulchella	ERR7619823	ZSS 10−1681−0	Micropuntia (Grusonia) pulchella P09237
P04626	Myrtillocactus geometrizans	Myrtillocactus geometrizans		Myrtillocactus geometrizans	ERR7618221	ZSS 94−1172−0	Myrtillocactus geometrizans P04626
P04627	Neolloydia conoidea	Neolloydia conoidea		Neolloydia conoidea	ERR7618222	K 1998−366	Neolloydia conoidea P04627
P04629	Neowerdermannia vorwerkii	Neowerdermannia vorwerkii		Neowerdermannia vorwerkii	ERR7618223	ZSS 99−8506−a	Neowerdermannia vorwerkii P04629
P05042	Nyctocereus serpentinus	Peniocereus serpentinus	Nyctocereus	Nyctocereus serpentinus	ERR7618274	K 1986−1051	Nyctocereus (Peniocereus) serpentinus P05042
P04566	Opuntia ficus-indica	Opuntia ficus-indica	ss	Opuntia ficus-indica	ERR7618187	ZSS 13−0042−b	Opuntia−ss ficus−indica P04566
P04554	Oreocereus pseudofossulatus	Oreocereus pseudofossulatus		Oreocereus pseudofossulatus	ERR7618178	ZSS 99−6008−0	Oreocereus pseudofossulatus P04554
P04631	Oroya peruviana	Oroya peruviana		Oroya peruviana	ERR7618224	ZSS 92−2847−0	Oroya peruviana P04631
P04567	Parodia graessneri	Parodia graessneri	Acanthocephala, Brasilicactus	Parodia haselbergii	ERR7618188	ZSS 16−0279	Parodia−AcanthocephalaBrasilicactus graessneri P04567
P04635	Parodia microsperma	Parodia microsperma	ss	Parodia microsperma	ERR5033447	ZSS 99−7076−0	Parodia−ss microsperma P04635

P04634	Parodia ottonis	Parodia ottonis	Notocactus	Parodia ottonis	ERR7618226	ZSS 96-2017-a	Parodia-Notocactus ottonis P04634
P04633	Parodia schumanniana	Parodia schumanniana	Eriocephala, Eriocactus	Parodia schumanniana	ERR7618225	ZSS 90-3155-0	Parodia-EriocephalaEriocactus schumanniana P04633
P04568	Parodia sellowii	Parodia sellowii	Wigginsia	Parodia erinacea	ERR7618189	ZSS 89-3232-a	Parodia-Wigginsia sellowii P04568
P04636	Pelecyphora aselliformis	Pelecyphora aselliformis		Pelecyphora aselliformis	ERR7618227	ZSS 78-2677-0	Pelecyphora aselliformis P04636
SRR7905861	Peniocereus cuixmalensis	Peniocereus cuixmalensis	ss	Peniocereus cuixmalensis	SRR7905861	SAMN10132140	Peniocereus-ss cuixmalensis SRR7905861 ncbi
SRR5137212	Pereskia humboldtii	Pereskia humboldtii	ss	Pereskia humboldtii	SRR5137212	SAMN06196472	Pereskia-ss humboldtii SRR5137212 ncbi
P04637	Pereskia stenantha	Pereskia stenantha	ss	Rhodocactus stenanthus	ERR5034692	ZSS 86-4200-0	Pereskia-ss stenantha P04637
P09253	Pereskiopsis porteri	Pereskiopsis porteri		Pereskiopsis porteri	ERR7619831	ZSS 92-1623-a	Pereskiopsis porteri P09253
P09255	Pfeiffera ianothele	Pfeiffera ianothele	ss	Pfeiffera ianthothele	ERR7619832	ZSS Hun-594	Pfeiffera-ss ianothele P09255
P04638	Pilosocereus leucocephalus	Pilosocereus leucocephalus	ss	Pilosocereus leucocephalus	ERR7618228	ZSS 95-1447-0	Pilosocereus-ss leucocephalus P04638
P04639	Polaskia chichipe	Polaskia chichipe		Polaskia chichipe	ERR7618229	ZSS 80-5101-0	Polaskia chichipe P04639
P13501	Praecereus euchlorus	Praecereus euchlorus		Praecereus euchlorus	ERR7620900	ZSS 89-2295-0	Praecereus euchlorus P13501
P04555	Pseudorhipsalis horichii	Pseudorhipsalis horichii		Pseudorhipsalis acuminata	ERR7618179	ZSS 85-3295-0	Pseudorhipsalis horichii P04555
P04571	Pterocactus tuberosus	Pterocactus tuberosus		Pterocactus tuberosus	ERR5033442	ZSS 96-1174-a	Pterocactus tuberosus P04571
P09193	Rapicactus subterraneus	Turbinicarpus subterraneus	Rapicactus	Rapicactus mandragora	ERR7619803	ZSS 16-0235-0	Rapicactus (Turbinicarpus) subterraneus P09193
P09181	Rebutia haagei	Rebutia haagei	Digitorebutia	Aylostera pygmaea	ERR7619797	ZSS 99-6546-0	Rebutia-Digitorebutia haagei P09181
P04642	Rebutia minuscula	Rebutia minuscula	ss	Rebutia minuscula	ERR7618231	ZSS 99-6563-c	Rebutia-ss minuscula P04642
P04599	Reicheocactus famatimensis	Echinopsis famatimensis	Reicheocactus	Echinopsis famatimensis	ERR7618204	ZSS 92-1806-a	Reicheocactus (Echinopsis) famatimensis P04599
P04644	Rhipsalidopsis rosea	Hatiora rosea	Rhipsalidopsis	Schlumbergera rosea	ERR5033448	ZSS 92-3016-0	Rhipsalidopsis (Hatiora) rosea P04644
P09259	Rhipsalis baccifera	Rhipsalis baccifera		unidentified	ERR7619834	ZSS 98-1270-0	Rhipsalis baccifera P09259
P07498	Rhipsalis teres	Rhipsalis teres		Rhipsalis teres	ERR7619464	GENT 20011223	Rhipsalis teres P07498
SRR7905862	Salmonopuntia salmiana	Salmonopuntia salmiana		Salmonopuntia salmiana	SRR7905862	SAMN10132143	Salmonopuntia salmiana SRR7905862 ncbi
P04646	Samaipaticereus corroanus	Samaipaticereus corroanus		Samaipaticereus corroanus	ERR7618232	ZSS 90-3741-0	Samaipaticereus corroanus P04646
P09297	Schlumbergera russeliana	Schlumbergera russeliana		Schlumbergera russelliana	ERR7619850	ZSS 96-1386-0	Schlumbergera russeliana P09297
P09261	Sclerocactus scheeri	Sclerocactus scheeri	Ancistrocactus	Sclerocactus scheeri	ERR7619835	ZSS 14-0126-0	Sclerocactus-Ancistrocactus scheeri P09261
P04647	Sclerocactus spinosior	Sclerocactus spinosior	ss	Sclerocactus spinosior	ERR7618233	ZSS 99-4240-0	Sclerocactus-ss spinosior P04647
P04556	Sclerocactus whipplei	Sclerocactus whipplei	ss	Sclerocactus whipplei	ERR7618180	ZSS 99-4217-0	Sclerocactus-ss whipplei P04556
P04648	Selenicereus grandiflorus	Selenicereus grandiflorus	ss	Selenicereus grandiflorus	ERR7618234	ZSS 99-8198-0	Selenicereus-ss grandiflorus P04648
P09265	Selenicereus testudo	Selenicereus testudo	Deamia	Deamia testudo	ERR7619837	ZSS 10-0050-0	Selenicereus-Deamia testudo P09265
P09217	Setiechinopsis mirabilis	Echinopsis mirabilis	Setiechinopsis	Echinopsis mirabilis	ERR7619813	ZSS HUN-403	Setiechinopsis (Echinopsis) mirabilis P09217
P04649	Stenocactus obvallatus	Stenocactus obvallatus		Stenocactus obvallatus	ERR7618235	K 1985-342	Stenocactus obvallatus P04649
P09301	Stenocereus standleyi	Stenocereus standleyi	Ritterocereus	Stenocereus standleyi	ERR7619852	ZSS 89-3696-0	Stenocereus-Ritterocereus standleyi P09301
SRR5137213	Stenocereus thurberi	Stenocereus thurberi		Stenocereus thurberi	SRR5137213	SAMN06196471	Stenocereus thurberi SRR5137213 ncbi
P04652	Stephanocereus leucostele	Stephanocereus leucostele		Stephanocereus leucostele	ERR7618236	ZSS 99-7350-0	Stephanocereus leucostele P04652
P04653	Stetsonia coryne	Stetsonia coryne		Stetsonia coryne	ERR7618237	ZSS 82-3648-d	Stetsonia coryne P04653
P04654	Strombocactus disciformis	Strombocactus disciformis		Strombocactus disciformis	ERR7618238	K 1999-211	Strombocactus disciformis P04654
P04640	Strophocactus brasiliensis	Strophocactus brasiliensis	Pseudoacanthocereus	Pseudoacanthocereus brasiliensis	ERR7618230	ZSS 88-2838-a	Strophocactus-Pseudoacanthocereus brasiliensis P04640
P04657	Tacinga funalis	Tacinga funalis		Tacinga funalis	ERR7618239	ZSS 86-4377-0	Tacinga funalis P04657
P09315	Talinaria coahuilensis	Talinaria coahuilensis		Anacampseros coahuilensis	ERR7619856	ZSS 90-1259-b	Talinaria coahuilensis P09315
P09313	Talinopsis frutescens	Talinopsis frutescens		Talinopsis frutescens	ERR12916368	ZSS 92-1739-a	Talinopsis frutescens P09313
P09311	Talinum triangulare	Talinum triangulare		Talinum fruticosum	ERR7619855	ZSS 18-0128-0	Talinum triangulare P09311
P09267	Tephrocactus articulatus	Tephrocactus articulatus	ss	Tephrocactus articulatus	ERR7619838	ZSS 86-2064-0	Tephrocactus-ss articulatus P09267

P09269	Tephrocactus verschaffeltii	Tephrocactus verschaffeltii	Banfiopuntia	Tephrocactus verschaffeltii	ERR7619839	ZSS 10−1882−0	Tephrocactus−Banfiopuntia verschaffeltii P09269
P04658	Thelocactus freudenbergeri	Thelocactus freudenbergeri	ss	spermatophyte root	ERR7618240	K 1999−683	Thelocactus−ss freudenbergeri P04658
P04660	Thelocactus hexaedrophorus	Thelocactus hexaedrophorus	ss	Thelocactus hexaedrophorus	ERR7618241	ZSS 87−1765−0	Thelocactus−ss hexaedrophorus P04660
P09271	Turbinicarpus horripilus	Turbinicarpus horripilus	Gymnocactus	Kadenicarpus horripilus	ERR7619840	ZSS TA−2299	Turbinicarpus−Gymnocactus horripilus P09271
P04662	Turbinicarpus schmiedickeanus	Turbinicarpus schmiedickeanus	ss	Turbinicarpus schmiedickeanus	ERR7618243	K 1999−702	Turbinicarpus−ss schmiedickeanus P04662
P04664	Vatricania guentheri	Vatricania guentheri		Espostoa guentheri	ERR7618244	ZSS 94−2200−0	Vatricania guentheri P04664
P09295	Weberbauerocereus weberbaueri	Weberbauerocereus weberbaueri		Neoraimondia arequipensis	ERR7619849	ZSS 94−2289−0	Weberbauerocereus weberbaueri P09295
P04665	Weberocereus frohningiorum	Weberocereus frohningiorum	−unplaced−	Haageocereus chilensis	ERR7618245	ZSS 11−0755−0	Weberocereus−−unplaced− frohningiorum P04665
P05047	Weberocereus tonduzii	Weberocereus tonduzii	Werckleocereus	Weberocereus tonduzii	ERR7618276	K 2007−1368	Weberocereus−Werckleocereus tonduzii P05047
P04668	Weingartia fidana	Weingartia fidana	ss	Rebutia fidaiana	ERR7618248	ZSS 99−9330−0	Weingartia−ss fidana P04668
P04666	Weingartia neocumingii	Weingartia neocumingii	ss	Rebutia neocumingii	ERR7618246	ZSS 85−2958−0	Weingartia−ss neocumingii P04666
P09279	Weingartia pulquinensis	Weingartia pulquinensis	Gymnorebutia	Rebutia neocumingii subsp. pulquinensis	ERR12916367	ZSS 85−2925−b	Weingartia−Gymnorebutia pulquinensis P09279
P04667	Weingartia steinbachii	Weingartia steinbachii	Sulcorebutia	Rebutia steinbachii	ERR7618247	ZSS 91−2484−0	Weingartia−Sulcorebutia steinbachii P04667
P09199	Winterocereus aureispinus	Cleistocactus winteri	Hildewintera, Winterocereus	Cleistocactus winteri	ERR7619806	ZSS 80−3277−0	Winterocereus aureispinus (Cleistocactus winteri) P09199
P05043	Xiquexique gounellei	Pilosocereus gounellei	Xiquexique	Xiquexique gounellei	ERR7618275	K 2002−3078	Xiquexique (Pilosocereus) gounellei P05043
P09281	Yavia cryptocarpa	Yavia cryptocarpa		Yavia cryptocarpa	ERR7619842	ZSS 99−7311−0	Yavia cryptocarpa P09281
P09283	Yungasocereus inquisiviensis	Yungasocereus inquisiviensis		Yungasocereus inquisivensis	ERR7619843	ZSS 96−2070−0	Yungasocereus inquisiviensis P09283
