# Supplementary material for: Phylogenomics and classification of Cactaceae based on hundreds of nuclear genes
Source: Plant Syst Evol. 2025 Aug 11;311(5):28. doi: 10.1007/s00606-025-01948-z (PMC12339657; doi:10.1007/s00606-025-01948-z)
Supplement: Supplementary file 3 — Online Resource 3: Containing locus and accession information for the BUCKy dataset (PDF 46 KB) [file 606_2025_1948_MOESM3_ESM.pdf]

Electronic Supplementary Material belonging to: Phylogenomics and classification of Cactaceae based on hundreds of nuclear genes; Plant Systematics and Evolution;
Jurriaan M. de Vos, Urs Eggli, Reto Nyffeler, Isabel Larridon, Catherine McGinnie, Niroshini Epitawalage, Olivier Maurin, Felix Forest and William J. Baker;
Corresponding author Jurriaan M. de Vos, University of Basel, email jurriaan.devos@unibas.ch.

Locus number	Locus name	Included in the BUCKy analysis[1]
1	4932	yes
2	4992	yes
3	5163	yes
4	5257	yes
5	5273	yes
6	5326	yes
7	5333	yes
8	5398	yes
9	5426	yes
10	5449	yes
11	5614	yes
12	5639	yes
13	5664	yes
14	5716	yes
15	5816	yes
16	5910	yes
17	5921	yes
18	5960	yes
19	6051	yes
20	6119	yes
21	6176	yes
22	6295	yes
23	6459	yes
24	6487	yes
25	6500	yes
26	6652	yes
27	6717	yes
28	6961	yes

1. The following accessions were selected to represent all higher taxa: Anacampserotaceae P9321, P9319, P9315; Leuenbergerioideae P4619; Pereskioideae−ss SRR5137212, P4637; Maihuenioideae SRR7905849; Blossfeldioideae P8439; Cactoideae−Copiapoeae P8881; Cactoideae−Cacteae P9261; Cactoideae−Phyllocacteae P9255; Cactoideae−Lymanbensonieae P9185; Cactoideae−Fraileeae P9233; Cactoideae−Rhipsalideae P4563; Cactoideae−Cereeae P9183; Cactoideae−Notocacteae P5169; Opuntioideae−Cylindropuntieae P9253; Opuntioideae−Pterocacteae P4571; Opuntioideae−Opuntieae P4585. See Online Resource 1 for corresponding voucher information and Online Resource 4 for MrBayes .t files as input.
