# Supplementary material for: Phylogenomics and classification of Cactaceae based on hundreds of nuclear genes
Source: Plant Syst Evol. 2025 Aug 11;311(5):28. doi: 10.1007/s00606-025-01948-z (PMC12339657; doi:10.1007/s00606-025-01948-z)
Supplement: Supplementary file 7 — Online Resource 7: Containing the ASTRAL tree based on the QC-P-BS40 data set (PDF 498 KB) [file 606_2025_1948_MOESM7_ESM.pdf]

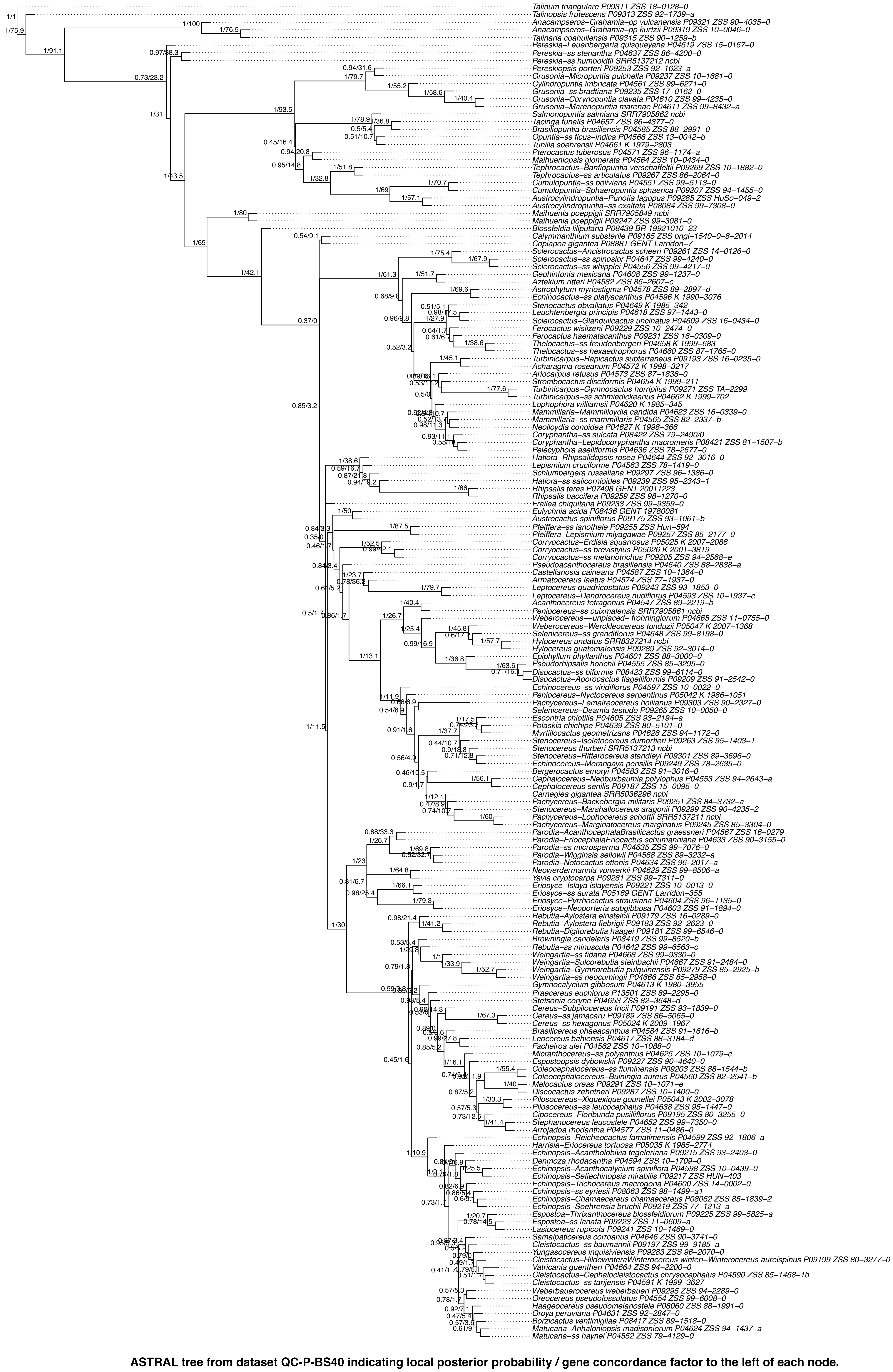

ASTRAL tree from dataset QC-P-BS40 indicating local posterior probability / gene concordance factor to the left of each node.

Electronic Supplementary Material belonging to: Phylogenomics and classification of Cactaceae based on hundreds of nuclear genes

Plant Systematics and Evolution

Jurriaan M. de Vos, Urs Eggli, Reto Nyffeler, Isabel Larridon, Catherine McGinnie, Niroshini Epitawalage, Olivier Maurin, Felix Forest and William J. Baker
Corresponding author Jurriaan M. de Vos, University of Basel, email jurriaan.devos@unibas.ch.
